# Design of a palette of SNAP-tag mimics of fluorescent proteins and their use as cell reporters

**DOI:** 10.1038/s41421-023-00546-y

**Published:** 2023-06-13

**Authors:** Dasheng Zhang, Zhengda Chen, Zengmin Du, Bingkun Bao, Ni Su, Xianjun Chen, Yihui Ge, Qiuning Lin, Lipeng Yang, Yujie Hua, Shuo Wang, Xin Hua, Fangting Zuo, Ningfeng Li, Renmei Liu, Li Jiang, Chunyan Bao, Yuzheng Zhao, Joseph Loscalzo, Yi Yang, Linyong Zhu

**Affiliations:** 1 Optogenetics & Synthetic Biology Interdisciplinary Research Center, State Key Laboratory of Bioreactor Engineering, East China University of Science and Technology, Shanghai, China; 2 Shanghai Frontiers Science Center of Optogenetic Techniques for Cell Metabolism, School of Pharmacy, East China University of Science and Technology, Shanghai, China; 3 School of Biomedical Engineering, Shanghai Jiao Tong University, Shanghai, China; 4 Department of Medicine, Brigham and Women’s Hospital, Harvard Medical School, Boston, MA USA

**Keywords:** Biological techniques, Cellular imaging

## Abstract

Naturally occurring fluorescent proteins (FPs) are the most widely used tools for tracking cellular proteins and sensing cellular events. Here, we chemically evolved the self-labeling SNAP-tag into a palette of SNAP-tag mimics of fluorescent proteins (SmFPs) that possess bright, rapidly inducible fluorescence ranging from cyan to infrared. SmFPs are integral chemical-genetic entities based on the same fluorogenic principle as FPs, i.e., induction of fluorescence of non-emitting molecular rotors by conformational locking. We demonstrate the usefulness of these SmFPs in real-time tracking of protein expression, degradation, binding interactions, trafficking, and assembly, and show that these optimally designed SmFPs outperform FPs like GFP in many important ways. We further show that the fluorescence of circularly permuted SmFPs is sensitive to the conformational changes of their fusion partners, and that these fusion partners can be used for the development of single SmFP-based genetically encoded calcium sensors for live cell imaging.

## Introduction

Naturally occurring fluorescent proteins (FPs) are the most widely used tools for live cell molecular imaging^1,2^, offering easy ways to track gene expression, protein degradation, compartment identification, and specific cellular structures. FPs are also actively used to generate genetically encoded sensors (GESs) that manifest dynamic changes in fluorescence intensity or hue in response to ions, metabolites, protein conformation, and protein–protein interactions. After translation and folding, FPs auto-catalytically oxidize and cyclize their chromophore-forming tripeptide to generate fluorescence^3–5^ at emission wavelengths inherent to the amino acid sequences and tertiary structures of the FPs that are difficult to manipulate in cells on demand. Chromophore formation may take minutes to hours and requires oxygen^5^, preventing real-time monitoring of rapid protein synthesis^5^ and applications under anaerobic conditions^3^. Furthermore, FPs emitting in the far red or infrared region, which usually requires larger D-π-A conjugated structures, are difficult to engineer as only four out of twenty naturally occurring amino acids contain aromatic residues (Phe, Tyr, Trp, and His)^6^. Despite two decades of extensive discovery and protein engineering to optimize the fluorescent range and application of FPs, many of these issues remained unresolved.

The GFP chromophore 4-hydroxybenzylidene imidazolinone (HBI) is only highly fluorescent when covalently bound within the naturally folded β-barrel structure of GFP, but not in solution^7^. Intriguingly, fluorescence of HBI analogs can be activated by RNA mimics (aptamers) of GFP (RMFPs)^6,8,9^ and a recently reported fluorogenic β-barrel termed as mFAP designed de novo via computational approaches^10^. Non-fluorescent endogenous ligands such as bilirubin and biliverdin show strong fluorescence upon binding with UnaG, a naturally occurred FP isolated from eel^11^, or near-IR FPs derived from bacteriophytochromes^12–14^, respectively. A few non-fluorescent exogenous fluorogens display fluorescence activation upon binding fluorogen-activating proteins engineered from single-chain antibodies or photoactive yellow protein^15–17^. The chromophores of these FPs, fluorogenic proteins or RNAs all belong to the family of molecules termed molecular rotors, which are non-fluorescent in solution owing to rapid intramolecular motion and energy dissipation of the excited state, but fluoresce when these motions are constrained upon protein or RNA binding. Although these RMFPs or mFAP show limited brightness in cells and need considerable improvement for wide biological applications^6,8–10^, they demonstrate the feasibility of engineering FP-like entities. Using entirely new fluorophore functionalities, we recently developed and chemically evolved Peppers, a series of fluorescent RNAs (FRs) that showed much improved cellular fluorescence brightness, fluorescence turn-on ratio (defined as the ratio of the fluorescence intensity of protein- or RNA-fluorophore complex to that of fluorophore alone), and a broad spectral range compared to currently available RMFPs, and demonstrated their advantages for live cell studies^9^. Inspired by these unique molecular properties, we herein exploited the possibility of creating SNAP-tag mimics of fluorescent proteins (SmFPs) by chemically evolving existing self-labeling proteins with new fluorophore functionalities, combining synthetic chemistry with genetic engineering.

## Results

### Construction of SmFP

We first investigated whether the intramolecular motions of HBI-like chromophores could be suppressed, and their fluorescence could be activated, when they are coupled to different self-labeling proteins^18^. To this end, we attached an HBI-like chromophore, 4-(N-hydroxyethyl-N-methyl-amino)-benzylidene imidazolinone (ABI), to specific covalent labeling ligands: *O*^*6*^-benzylguanine (BG) for SNAP_f_, a variant of SNAP-tag derived from *O*^*6*^-alkylguanine-DNA-alkyltransferase with faster labeling kinetics^19,20^; chloroalkane for Halo-tag, a modified haloalkane dehalogenase^21^; and second-generation trimethoprim (TMP) for eDHFR, *Escherichia coli*-derived dihydrofolate reductase^22^. All of these ABI derivatives correctly labeled their corresponding protein fusion partners as shown by SDS polyacrylamide gel electrophoresis (Supplementary Fig. S1a, b). Significant fluorescence enhancement was observed only for the ABI–SNAP_f_ complex, but not for their ABI-Halo-tag or the ABI–TMP complex (Supplementary Fig. S1c–e), suggesting that among these self-labeling proteins, only the binding site of SNAP_f_ is capable of restricting intramolecular motion of ABI. ABI-BG was able to highlight SNAP_f_-expressing cells under non-washing conditions (Supplementary Fig. S1f); however, the quantum yield of ABI-SNAP_f_ (*φ* = 0.0067) is two orders of magnitude lower than that of FPs, seriously limiting its use for protein labeling.

**Fig. 1:**
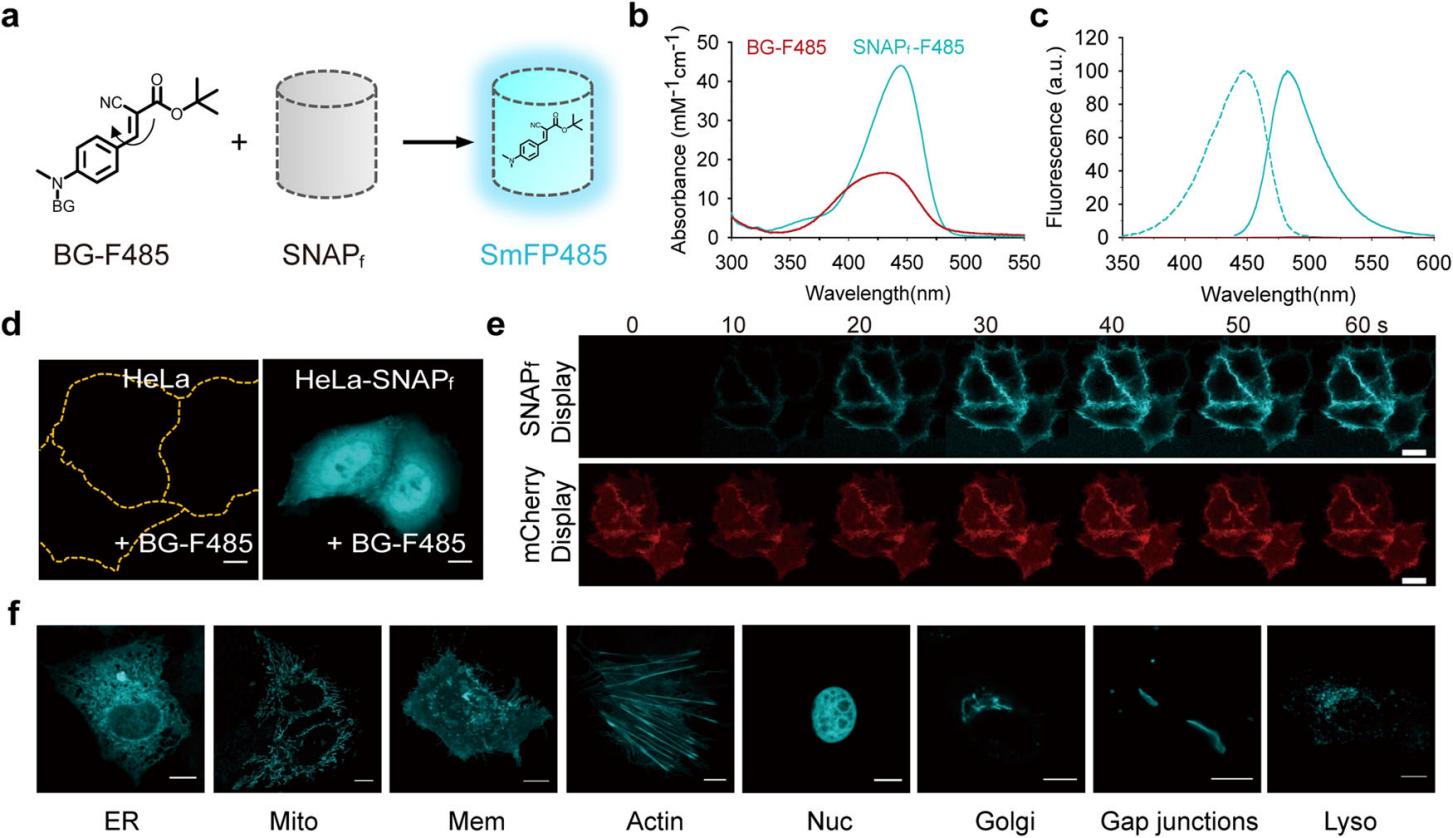
Construction of a cyan SmFP. **a** Schematic representation of a cyan SmFP (F485-SNAP_f_ complex, SmFP485). The synthetic, non-fluorescent BG-F485 fluorophore became fluorescent once it formed a covalent complex with SNAP_f_ protein. **b** Absorption spectra of 5 μM BG-F485 (red) or SmFP485 (cyan). **c** Excitation (dashed) and emission (solid) spectra of 5 μM BG-F485 (red) or SmFP485 (cyan). **d** Fluorescent images of SmFP485-expressing cells. HeLa cells transfected with or without pcDNA3.1-SNAP_f_ vector, and imaged after incubation with 2 μM BG-F485. Scale bars, 10 μm. **e** Images of SmFP485 fluorescence generation in sequential frames in cells expressing plasma membrane-localized SNAP_f_. HeLa cells co-transfected with pDisplay-SNAP_f_ and pDisplay-mCherry were imaged after incubation with 2 μM BG-F485. Scale bars, 20 μm. **f** Fluorogenic labeling of subcellular targeted SNAP_f_ fusions recorded without washing. Scale bars, 10 μm.

DCN, 4-(dialkylamino)-benzylidene)-malononitrile, is a classical small-molecule molecular rotor, which emits strong fluorescence upon constraining intramolecular motion^23,24^. The conjugate of DCN and BG, BG-DCN (Supplementary Fig. S1g), showed no detectable fluorescent emission in aqueous buffer (Supplementary Fig. S1h). Addition of BG-DCN to SNAP_f_ protein, however, resulted in immediate emission of cyan fluorescence at 490 nm, manifesting a marked increase in fluorescence intensity compared to free BG-DCN of 54-fold (Supplementary Fig. S1h). We next sought to enhance the fluorescence intensity of the BG-DCN-labeled SNAP_f_ by modifying the DCN dye moiety (Supplementary Fig. S1i). The most promising derivative, BG-F485, in which one cyano-group of the DCN dye was replaced with a *t*-butyl ester, showed a specific fluorescence intensity increase of over 350-fold upon reaction with SNAP_f_ (Fig. 1a; Supplementary Fig. S1i, j and Table S1). Kinetic studies showed that the second-order rate constant (k_2_) for the reaction was 17,000 ± 1000 M^−1^ s^−1^ (Supplementary Fig. S1k, l), which is comparable to those reported with other SNAP_f_ labels^19,25,26^. The F485-SNAP_f_ complex displayed a well-defined absorption profile with a single peak at 443 nm (*ε*_440_ = 44, 000 M^−1^ cm^−1^) (Fig. 1b; Supplementary Table S1). Binding not only markedly enhanced but also red-shifted the absorption of BG-F485 by 12 nm, suggesting strong interaction between the chromophore and the protein. Compared to previously developed cyan fluorescent proteins, the cellular brightness of SmFP485 was 159%, 75%, and 64% that of ECFP, mCerulean3, and mTurquoise2, respectively (Supplementary Fig. S1m, n), and significantly lower photobleaching rate (Supplementary Fig. S1o).

Unlike many conditional fluorophores that can also be activated nonspecifically, i.e., by lipids and abundant cellular proteins with comparatively nonspecific hydrophobic binding sites^27^, BG-F485 remained non-fluorescent upon mixing with bovine serum albumin (Supplementary Fig. S1j), a well-known hydrophobic molecule binding protein that activates the fluorescence of various solvatochromic dyes. The compound was almost undetectable fluorescent upon incubation with control cells, whereas its cyan fluorescence was visualized in cells expressing SNAP_f_ targeted to various subcellular compartments (Fig. 1d, f). Upon addition of 2 μM BG-F485, the SNAP_f_ fusion was readily visualized in live cells within 30 s (Fig. 1e; Supplementary Fig. S1p–r).

### Real-time tracing of protein expression, degradation, and interaction using SmFP

**Fig. 2:**
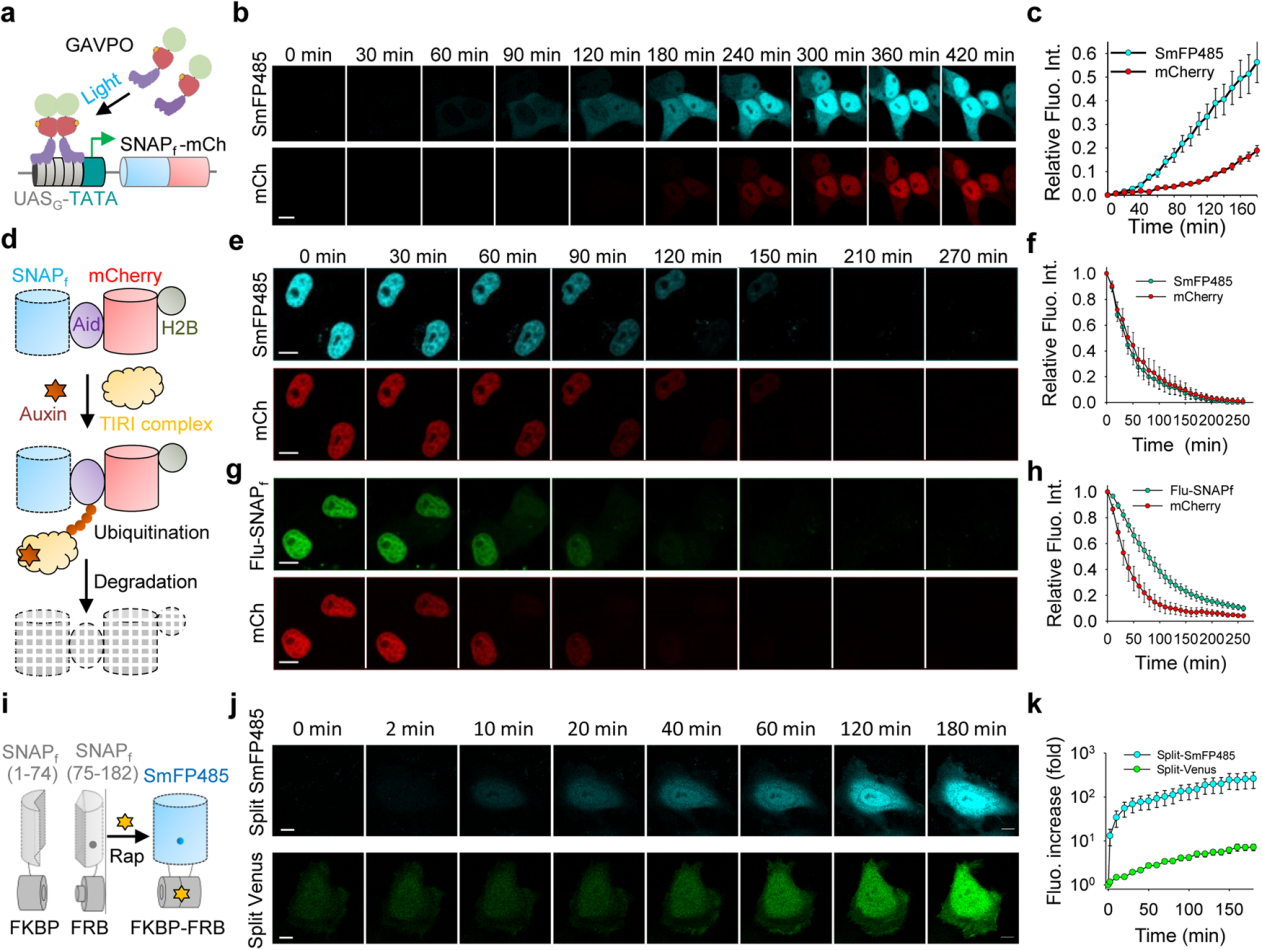
Real-time tracing of protein expression, degradation, and protein–protein interaction using SmFP485. **a** Schematic representation of light-induced SmFP expression based on the LightOn system. Upon blue light exposure, GAVPO binds to the UAS_G_ sequence and activates the transcription of the SNAP_f_-mCherry fusion gene. **b** Consecutive live cell imaging of SmFP485-mCherry fusion protein expression induced by light. Scale bar, 10 μm. **c** Quantitative analysis of SmFP485 and mCherry fluorescence during light-induced protein expression. Data were normalized to the 7 h time point sample. Error bars are standard deviations, *n* = 10 cells. **d** Schematic representation of nuclear-targeted SNAP_f_-mCherry fusion protein degradation under the control of the AID system. Auxin binding to TIR1 promotes the interaction between TIR1 and AID within the SNAP_f_-Aid-mCherry-histone fusion protein, resulting in polyubiquitination of the AID degron and degradation by the proteasome. **e**–**h** Consecutive imaging of SNAP_f_-Aid-mCherry-histone degradation in cells treated with 500 μM auxin. SNAP_f_ protein was labeled with BG-F485 (**e**) or BG-Fluorescein (**g**) to generate SmFP485 and Fluorescein-SNAP_f_ (Flu-SNAP_f_), respectively. Scale bars, 10 μm. Quantitative analysis of SmFP485 and mCherry fluorescence (**f**) or fluorescein and mCherry fluorescence (**h**) during auxin-induced protein degradation. Data were normalized to the 0 min time point. Error bars are standard deviations, *n* = 10 cells. **i** Schematic representation of bimolecular fluorescence complementation (BiFC) using SmFP485 fragments. The N-terminal end (1–74 amino acids) and C-terminal end (75–182 amino acids) of SNAP_f_ were fused to FRB and FKBP, respectively. Rapamycin-induced protein interaction and the generation of SmFP485 fluorescence. **j** Consecutive imaging of rapamycin-induced fluorescence of the SmFP485 fragment or the Venus fragment. Scale bars, 10 μm. **k** Quantitative analysis of SmFP485 and Venus fluorescence signal after rapamycin-induced interaction of FRB and FKBP. Error bars are standard deviations, *n* = 20 cells.

The rapid labeling of SNAP_f_ by BG-F485 allows reporting of protein synthesis in real time. By contrast, it may take hours for GFP-like FPs to mature and emit fluorescence. During cell-free expression of a fusion between SNAP_f_ and mCherry, we could observe fluorescence of the F485-SNAP_f_ complex within 2 min after the initiation of protein synthesis, whereas mCherry fluorescence only began to appear after 40 min (Supplementary Fig. S2a). In live cells, F485-SNAP_f_ fluorescence was readily observed compared to mCherry fluorescence (Fig. 2a–c; Supplementary Fig. S2b) when the fusion protein was induced by light under the control of the GAVPO light-sensitive transcription activator^28^. These data are consistent with the rapid labeling of SNAP_f_ and the comparatively slow maturation process of GFP-like FPs.

We also found that the fluorescence of the F485-SNAP_f_ complex diminished under denaturing conditions, such as treatment with heat or GdnHCl, suggesting that its fluorescence was highly dependent on the integrity of the protein’s tertiary structure (Supplementary Fig. S2c, d), similar to that of GFPs. We next used the auxin-inducible degron (AID) degradation system^29^ to assess the degradation kinetics of the nuclear-targeted SNAP_f_-mCherry-histone fusion protein in the cells. During protein degradation triggered by indole-3-acetic acid (IAA) treatment, the nuclear fluorescence of F485-SNAP_f_ decreased rapidly with a half-life of ≈ 40 min, together with the decrease of mCherry fluorescence (Fig. 2d–f; Supplementary Video S1). The fluorescence of SNAP_f_ labeled with fluorescein dye, however, not only decreased with a marked lag time compared to that of mCherry, but was also significantly distributed into the cytosol, likely because the fluorescein dye remained fluorescent even after the protein was degraded (Fig. 2g, h; Supplementary Video S2). These data suggest that the F485-SNAP_f_ complex behaved similarly to GFP-like proteins in protein degradation assays, i.e., their fluorescence diminished immediately upon degradation, which is a significant benefit of fluorescent proteins over traditional fluorescently labeled proteins.

Overall, the self-labeling of SNAP_f_ is highly specific, rapid, and intensely fluorogenic, enabling real-time tracing of protein expression and degradation in live cells under no-washing conditions. The fluorescence properties and their dependence on the protein’s integrity of the F485-SNAP_f_ complex resemble that of FPs, but with a much faster “maturation rate”. We, therefore, termed the self-labeled F485-SNAP_f_ complex as SmFP485, i.e., SmFP emitting at 485 nm.

Further studies showed that SmFP485 could be used similarly to GFP and other FPs in bimolecular fluorescence complementation assays (BiFC) to study protein–protein interactions^30^. To this end, we expressed fragmented SmFP485 separated at amino acids 74–75, and fused the two halves (SmFP485_N_ and SmFP485_C_) to the rapamycin binding domain of mTOR (FRB) and FK506 binding protein (FKBP), respectively (Fig. 2i). HeLa cells co-expressing SmFP485_N_-FRB and FKBP-SmFP485_C_ were essentially non-fluorescent (Supplementary Fig. S2e, f). Upon addition of rapamycin, the induced interaction of FRB and FKBP rapidly enhanced fluorescence in the cells. A BiFC contrast of 69-fold was achieved as early as 0.5 h after addition of rapamycin (Fig. 2j, k; Supplementary Fig. S2e, f), eventually up to 700-fold by FACS analysis (Supplementary Fig. S2h). In comparison, cells co-expressing Venus_N_-FRB and FKBP-Venus_C_ showed significant background fluorescence originating from nonspecific reporter fragment complementation (Supplementary Fig. S2g), and the rapamycin-induced interaction of FRB and FKBP induced slow fluorescence enhancement, with BiFC contrast of 2-fold at 0.5 h (Fig. 2j, k) and eventually of 11-fold after 24 h (Supplementary Fig. S2i). Although SmFP-based BiFC assay exhibited faster kinetics than FP-based BiFC assay, it still took more than 0.5 h for producing sufficient BiFC signal (Fig. 2j, k). Considering the fast kinetics for rapamycin-induced FKBP–FRB interaction^31^ (an association constant (Ka) of 1.92 × 10^6 ^M^−1^ s^−1^) and fast labeling of BG-F485 with SNAP_f_, the key factor limiting the kinetics of SmFP-based BiFC assay is probably the reconstitution of the split SmFP. It is well known that the major drawbacks of the BiFC system based on GFP-like proteins are 1) the ability of the two FP fragments to reassemble in the absence of a bona fide protein–protein interaction, and 2) the slow maturation of fluorescence, with neither limitation being satisfactorily resolved despite several years of technical modifications^32,33^. Thus, with more than one magnitude higher BiFC contrast and faster kinetics, split SmFP485 compares favorably to currently available reporter fragment pairs based on GFP-like FPs.

### Atomic structure of SmFP reveals mechanism of fluorescence activation

**Fig. 3:**
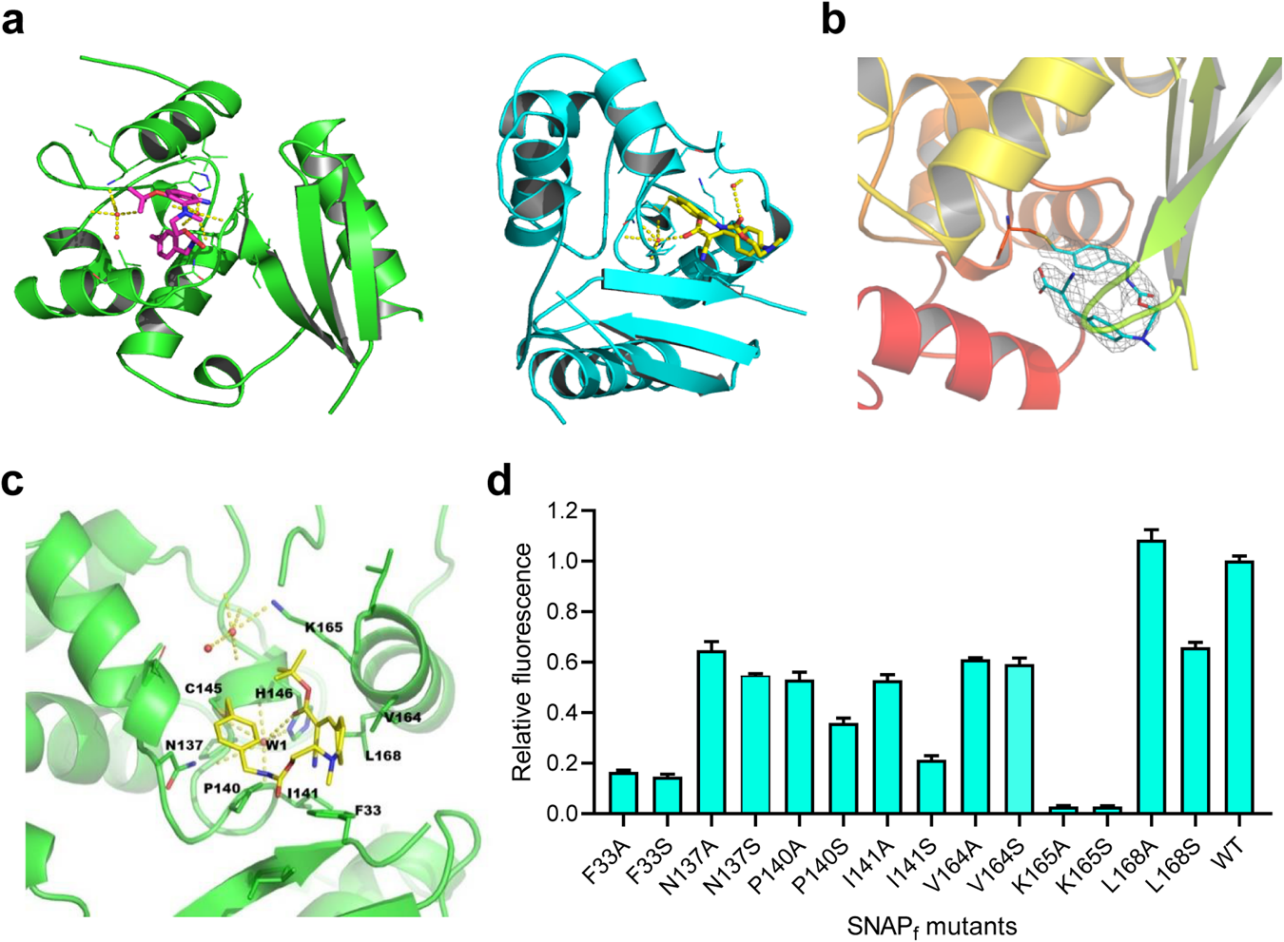
Crystal structure of SmFP485 reveals the mechanism of fluorescence activation. **a** Overall structure of SmFP485 (subunit A, green; subunit B, cyan). **b** Final refined model of the bound F485 is superimposed on the 2Fo-Fc electron density map contoured at 2.0*σ*. **c** Detailed view of the interactions of F485 with side chains of the binding pocket. Broken yellow lines, hydrogen bonds; orange spheres, water molecules. **d** Effect on fluorescence of mutations in the binding pocket. Error bars are standard deviations, *n* = 3.

SmFP485 was developed from human *O*^*6*^-alkylguanine-DNA alkyltransferase (AGT), which exhibits a two-domain α/β fold. Although the structures of both the Apo and benzoylated AGT or SNAP-tag are available^19^, it is not readily clear how the F485 dye in SmFP485 adopts its fluorescent conformation in the protein. To address this issue, we next solved the crystal structure of SmFP485 at 2.09 Å resolution (Fig. 3a; Supplementary Fig. S3a). Both SmFP485 monomers in the asymmetric unit were aligned with the template molecular structure (PDB ID: 3L00) (Supplementary Fig. S3b). Residues in the loop (G157–G161) covering the active site, especially Y158, flip away to accommodate the bulkier ligand (Fig. 3b). Mass spectroscopy demonstrated that the F485 dye is, indeed, contained in the SmFP485 crystal (Supplementary Fig. S3c). F485 dye covalently binds to C145 via the benzyl group, and then folds into a unique “U” shape with the fluorophore mostly buried in the small binding pocket. F485 dye forms hydrophobic interactions with V164, L168, I141, and P140, and its carbonyl group forms a hydrogen bond network with one water molecule and the backbone of C145, P144, P140, and H146. The phenyl group of F485 dye engages in a π–π interaction with residue F33 (Fig. 3c). By these interactions, the F485 dye is strongly locked in the binding pocket. Resembling the chromophore–protein interaction in natural FPs, such a highly restricted, unique conformation of the F485 fluorophore apparently suppresses the intramolecular motions of its cyanovinyl group, and, thus, supports its fluorescence.

We next investigated the contributions of the residues to the fluorescence activation of SmFP485. Substitution of F33, N137, P140, I141, V164, K165, and L168 with alanine or serine residues significantly decreased the fluorescence of SmFP485, but without affecting the covalent bond (Fig. 3d; Supplementary Fig. S3d). Notably, substitution of K165 with alanine or serine led to almost complete loss of fluorescence (Fig. 3d), probably because it prevented the formation of covalent bond between SNAP_f_ and fluorophore (Supplementary Fig. S3d). Intriguingly, substitution of L168 with serine slightly decreased the fluorescence, but substitution of L168 with alanine led to 10% increase in the fluorescence of SmFP485 (Fig. 3d; Supplementary Fig. S3d). These data suggest that the amino acids around the binding pocket are critical for stabilizing the conformation of the F485 fluorophore and for fluorescence activation, consistent with the crystal structure studies.

### Developmental chemistry of a palette of SmFPs

**Fig. 4:**
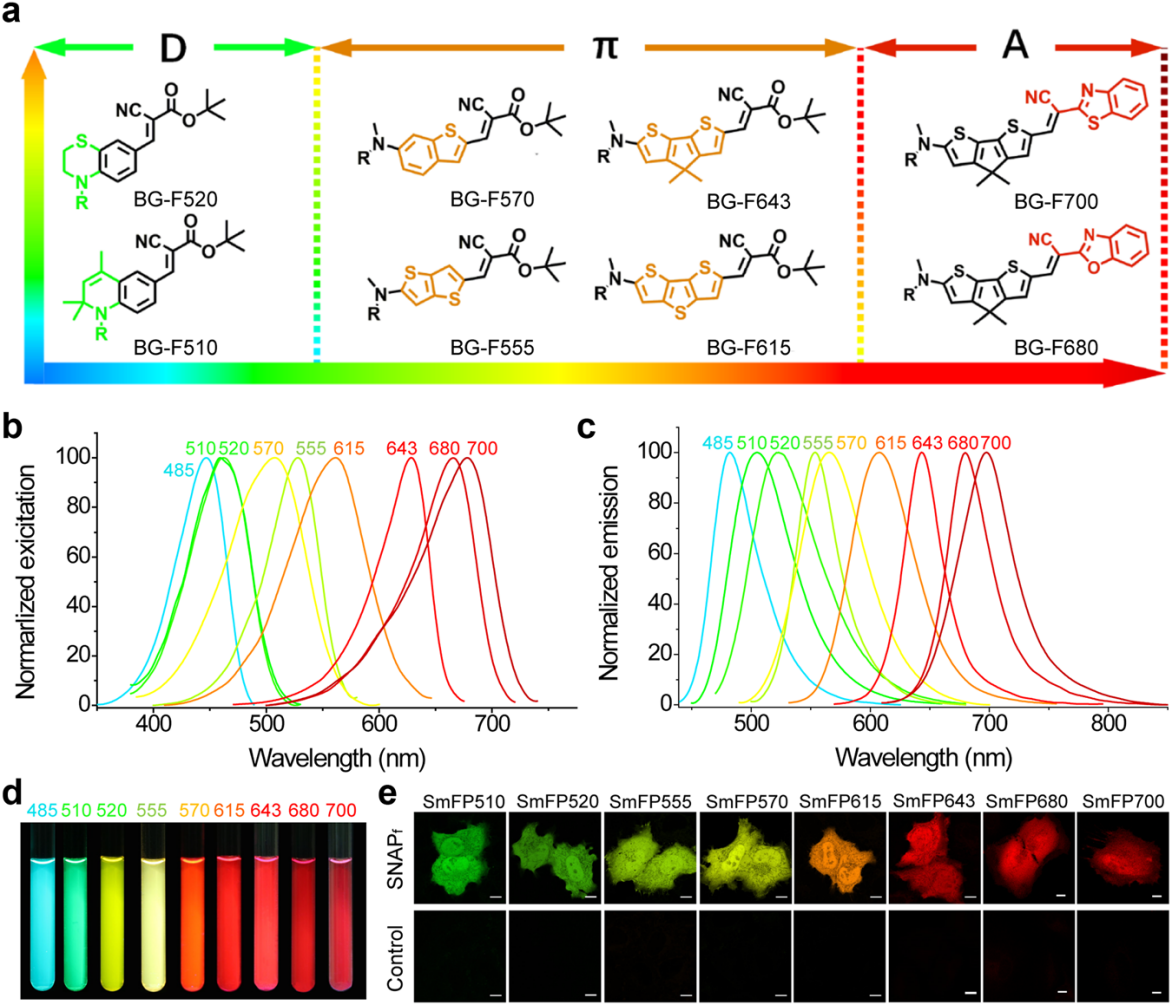
Developmental chemistry of a palette of SmFPs. **a** Chemical structures of BG-fluorophores with 510–700 nm emission. **b**, **c** Excitation spectra (**b**) and emission spectra (**c**) of the palette of SmFPs. **d** SmFPs (10 μM) were illuminated with ultra-violet light (365 nm) and photographed. **e** Live cell imaging of cells expressing SmFPs. Cells transfected with the empty vector were used as controls. Scale bars, 10 μm.

In the unique “U” conformation of the F485 fluorophore in SmFP485, the aromatic group of F485 is linked to the S-benzyl group by a flexible linker exposed to the solvent; we, therefore, reasoned that the bulkier F485 analogs might also be able to fit into the binding pocket of SmFP and retain their fluorogenic properties. To further optimize the spectral properties of SmFP, we synthesized a series of derivatives of BG-F485 according to the general principles of photochemistry and dye design (Fig. 4a). We first replaced the phenylamine moiety of BG-F485 with an unsaturated cycloalkyl group, a rigid and electron-donating enhanced group (BG-F510); or a saturated cycloalkyl group with an additional thiol group as an electron-donating group (BG-F520). Alternatively, π-conjugation could be extended to various condensed aromatic rings such as benzothiophene, bithiophene, and dithenothiophene (BG-F570, BG-F555, and BG-F615) (Fig. 4a). Furthermore, the sulfur in the middle thiophene ring of BG-F615 was replaced with a gem-dimethyl carbon (BG-F643) to lower the energy level of the excited state. Finally, the *t*-butyl ester in F643 was replaced by a 2-benzoxazole or a 2-benzothiazole to strengthen the electron-accepting capacity (BG-F680 or BG-F700). All of these compounds are highly fluorogenic in the locked conformation, i.e., they do not fluoresce in the free form, whereas SNAP_f_ labeled with these dyes emitted intense fluorescence with colors ranging from green to infrared (Fig. 4b–d; Supplementary Table S1). These data suggest that this series of dyes might adopt similar conformations in the binding pocket, with their fluorescence activation following a similar mechanism to SmFP485. Thus, we generated a palette of SmFPs, ranging from the visible to infrared spectrum, and denoted them according to their emission maxima (Supplementary Table S1). Further studies demonstrated that these SmFPs are capable of specifically and robustly labeling live cells under no-washing conditions (Fig. 4e; Supplementary Fig. S4a), without any significant cytotoxicity to the cells as shown by cell proliferation and viability assays (data not shown).

This series of SmFPs showed extinction coefficients ranging from 35,000 M^−1^ cm^−1^ to 78,000 M^−1^ cm^−1^, and quantum yields ranging from 0.35 to 0.76, similar to GFP-like FPs (Supplementary Table S1). These SmFPs showed much higher cellular brightness and signal-to-background ratios favorably compared to previously developed fluorogenic ligands for SNAP-tag^34–37^ (Supplementary Fig. S5). Notably, the SmFPs emitting red to infrared fluorescence have significantly enhanced quantum yields and fluorescence intensity compared to the best GFP-like far-infrared FPs or bacteriophytochrome-derived near-infrared FPs currently available. In HeLa cells, we observed that SmFP615 is 1.2-fold more intensely fluorescent than mCherry (Supplementary Fig. S4b), SmFP643 is 3.7-fold more intensely fluorescent than mKate2 (Supplementary Fig. S4c), and SmFP680/SmFP700 are 23.6-fold to 7.9-fold more intensely fluorescent than iRFP682/iRFP720, respectively (Supplementary Fig. S4d, e). Further studies showed that the PEGylated BG-F680 and BG-F700 also could be used for in vivo image of xenograft tumors expressing SNAP_f_, with much improved brightness and signal-to-background ratios compared to a near-infrared silicon-rhodamine probe developed previously^37^ when used at a longer wavelength of excitation and emission (Supplementary Fig. S6), which makes SmFP680 and SmFP700 more preferable for deep-tissue imaging.

### Real-time monitoring of protein assembly and trafficking via SmFP multi-color pulse-chase studies

**Fig. 5:**
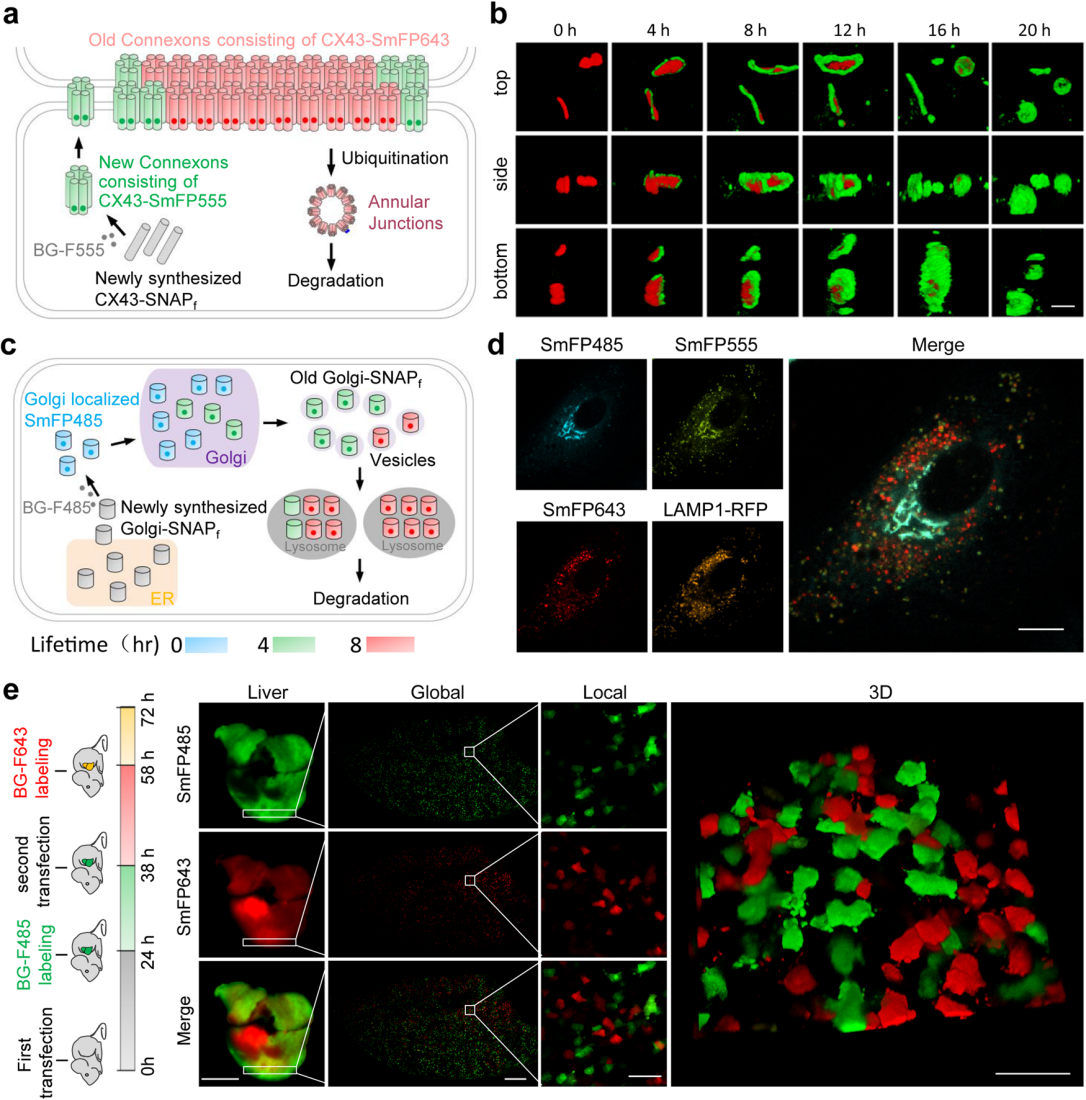
Real-time monitoring of protein expression, assembly, and trafficking using SmFP multi-color pulse-chase. **a** Schematic representation of CX43 synthesis and gap junction assembly. **b** Time-lapse imaging of newly synthesized CX43 protein incorporated in gap junctions. HeLa cells expressing CX43-SNAP_f_ were first labeled with 1 μM BG-555 for 30 min, washed twice with fresh medium to remove the unbound fluorophores, then labeled with 1 μM BG-F485 and consecutively imaged. Scale bar, 5 μm. **c** Schematic representation of Golgi-SNAP_f_ intracellular trafficking. **d** Multi-color pulse-chase images of Golgi-SNAP_f_ trafficking. HeLa cells co-transfected with plasmids coding for Golgi-SNAP_f_ and Lamp1-RFP were sequentially incubated with BG dyes each for 4 h; i.e., Golgi-SNAP_f_ of 0–4 h, 4–8 h, or > 8 h lifetime was labeled with BG-F485, BG-F555, or BG-F643, respectively. Scale bar, 10 μm. **e** Multi-color in vivo pulse-chase of SNAP_f_ synthesis in mouse livers. SNAP_f_ expressing plasmid was transfected into mouse liver at 0 h and 48 h by a hydrodynamic procedure. The mice received tail vein injection of 40 nmol BG-643 at 38 h and 20 nmol BG-485 at 58 h for SNAP_f_ labeling. Fluorescence imaging of mouse liver samples was carried out at 72 h. Scale bars for the liver, global, local, and 3D images were 5000 μm, 1000 μm, 100 μm, and 100 μm, respectively.

One of the distinctive features of protein self-labeling technologies is that they are suitable for pulse-chase labeling, which is a useful strategy for studying protein assembly and trafficking in live cells with subcellular resolution^38^. The pulse-chase experiments using traditional fluorophores, however, require washing steps during the course of the chase to remove the unbound probes; in contrast, SmFPs do not require a washout, thereby enabling one to monitor the assembly of newly synthesized protein in real time. For this reason, we studied gap junction dynamics using SNAP_f_ fused to the C-terminal end of connexin43 (CX43), which is the most abundant and widely expressed connexin and a protein that turns over rapidly^39^. Cells expressing CX43-SNAP_f_ were incubated with BG-F555 for 30 min, which labeled all the preexisting CX43-SNAP_f_ within the cell. The culture medium was next replaced with fresh medium containing BG-F485, and gap junction assembly was followed by confocal fluorescence microscopy (Fig. 5a). Three-dimensional reconstructions showed that while only red fluorescence of pre-existing gap junctions around cell-cell contacts can be observed at time zero, green fluorescence signals indicate newly synthesized CX43-SNAP_f_ protein associated with the periphery of the preexisting gap junction plaques (Fig. 5b). These green rings are noted to surround circular red core zones in a time-dependent manner. After 20 h of a chase, the majority of labeling in gap junctions consisted of de novo synthesized CX43 during the course of the chase (Fig. 5b; Supplementary Video S3). Compared to a biarsenical derivative of fluorescein^39^, SmFPs can be used to monitor protein assembly in real time over long periods of observation owing to the extremely low cytotoxicity and limited fluorescence photobleaching.

We next fused a widely used *trans*-Golgi targeting signal peptide derived from galactosyltransferase^40^ to SNAP_f_ (Golgi-SNAP_f_) and pulse-chased its trafficking in live cells by sequentially labeling with BG-F643, BG-F555, and BG-F485 for 4 h for each dye (Fig. 5c). Apparently, Golgi-SNAP_f_ expressed at different times showed distinct localizations in the snap-shot images (Fig. 5d; Supplementary Fig. S7a). The most recently synthesized Golgi-SNAP_f_ protein labeled as cyan was concentrated in large, intensely fluorescent flakes or tube-like structures, which likely represent Golgi stacks, together with thin, reticular structures distributed throughout the cytoplasm; whereas small vesicles appeared for older, more mature Golgi-SNAP_f_ proteins (Fig. 5d; Supplementary Fig. S7a, b). These vesicles co-localized well with the lysosome marker Lamp1-RFP fusion (Fig. 5d; Supplementary Fig. S7a and Video S4). When cells were pre-labeled with BG-F555 and then time-lapse images obtained in the presence of BG-F485, we observed that *trans*-Golgi stacks of a small population of cells can evidently be separated into two groups, consisting of older vs newly synthesized Golgi-SNAP_f_, respectively (Supplementary Fig. S7c). Thus, the lifespan of Golgi could be revealed by the SmFP multi-color pulse-chase, which rapidly traffics from the ER to Golgi stacks after being synthesized, then forms *trans*-Golgi vesicles, and finally is degraded in the lysosome. In similar live cell chase experiments using SmFPs, we found that although histone H2B was largely excluded from the nucleolus, the de novo synthesized H2B was mainly localized in the nucleolus (Supplementary Fig. S7d–f), and that fragmented mitochondria with spheroid-shaped morphology contained significantly less de novo synthesized protein than mitochondria with reticulated morphology in cells treated with mitochondrial (electron-transport) inhibitors (Supplementary Fig. S7g).

As a proof-of-principle, we further demonstrated the utility of SmFPs for in vivo chase studies of protein expression. To this end, a SNAP_f_-expressing plasmid was transfected into the livers of mice using a hydrodynamic procedure. Robust co-localization of SmFP485 and SmFP643 fluorescence in livers or single hepatocytes was observed when live mice were labeled with BG-F485 and BG-F643 simultaneously (Supplementary Fig. S7h), with little background fluorescence in the control livers transfected with empty plasmid (Supplementary Fig. S7i). For mice transfected sequentially with the SNAP_f_ gene at 0 h and 38 h, SNAP_f_ protein expressed as a result of the first transfection or the second transfection was labeled with BG-F485 at 24 h or BG-F643 at 58 h, respectively. We observed clearly separated distributions of SmFP485 and SmFP643 fluorescence in the whole livers or individual hepatocytes (Fig. 5e), while little SmFP643 fluorescence appeared in the livers receiving single transfection of SNAP_f_ gene (Supplementary Fig. S7j). Surprisingly, most hepatocytes displayed only one fluorescence color, either SmFP485 or SmFP643, without colocalization. Therefore, it seems that these cells developed ‘resistance’ to a second DNA transfection. These data demonstrate that the SmFPs provide a robust and discriminating way to follow protein dynamics in live animals.

### Genetically encoded calcium sensor based on SmFPs

**Fig. 6:**
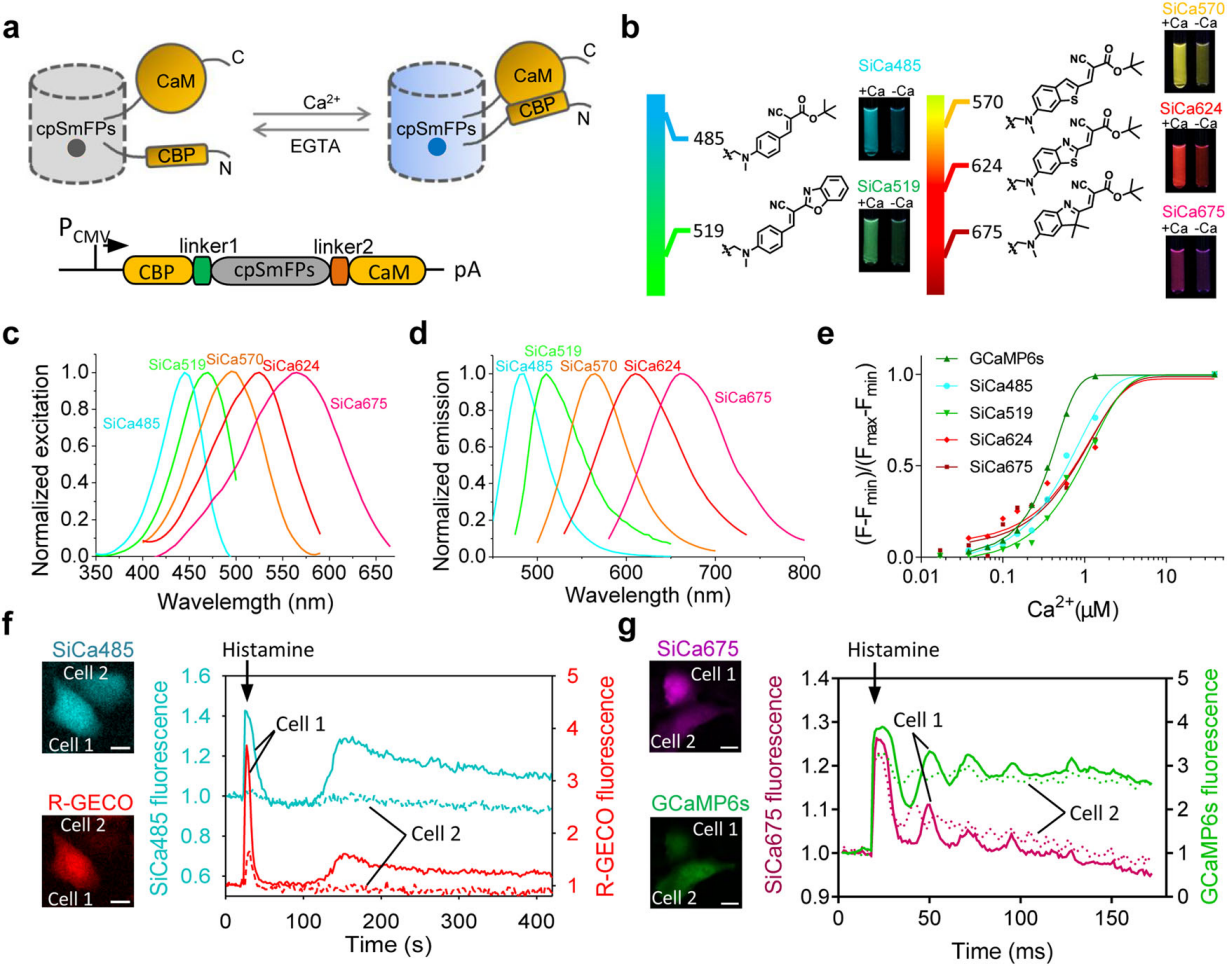
Genetically encoded calcium sensors based on single SmFP. **a** Schematic diagram of the synthetic indicators for Ca^2+^ based on circularly permuted SmFPs. The synthetic indicators consist of the calmodulin binding peptide (CBP), cpSNAP_f_, and calmodulin (CaM). **b** Chemical structures of fluorophores for the synthetic calcium indicators. Fluorescence of the synthetic calcium indicators in the Ca^2+^-free and Ca^2+^-bound states was shown. **c**, **d** Excitation spectra (**c**) and emission spectrum (**d**) of the synthetic calcium indicators. **e** Ca^2+^ titration of the synthetic calcium indicators at pH 7.4. Protein concentrations were 100 nM. Error bars are standard deviations, *n* = 3. **f**, **g** SiCa485 (**f**) and SiCa675 (**g**) reported Ca^2+^ dynamics in single HeLa cells treated with histamine. Scale bars, 10 μm.

In the widely used green GCaMP or maximal wavelength-shifted GECO series GES for calcium^41,42^, the circularly permuted (cp) FP is inserted between the M13 peptide and calmodulin (CaM). Calcium binding results in a change of the protein conformation and local environment around the chromophore of cpFP, strongly enhancing its fluorescence. We found that SmFP485 was also tolerant to circular permutation when the original N- and C-termini were linked through a flexible linker, rendering L34 and V44 new N- and C-termini, respectively. When cpFP in the GCaMP sensor was replaced with cp-SmFP485, the resulting fusion protein was also reversibly responsive to calcium binding (Fig. 6a). After creation and selection of a series of truncated variants and/or site-directed mutants, we identified the M13-cpSNAP_f_-CaM (P47/L34, F33W, W6F) variant as manifesting the most dramatic increase in fluorescence upon binding Ca^2+^ (Fig. 6b–e; Supplementary Fig. S8a, b), demonstrating a steep Ca^2+^ titration curve consistent with the known cooperativity in calcium binding to calmodulin’s four binding sites. We, therefore, termed this variant, SiCa485 (synthetic indicator of calcium). Further studies showed that SiCa485 has an association rate coefficient (*K*_on_) of 2.178 ± 0.193 × 10^6^ and a dissociation rate coefficient (*K*_off_) of 1.219 ± 0.108 s^−1^ (Supplementary Fig. S8g and Table S2). By adjusting the electron acceptor capability of F485, we obtained a green indicator of calcium termed SiCa519 (Fig. 6b–e; Supplementary Fig. S8c). We also constructed orange to far-red synthetic indicators for calcium based on cp-SmFP570 (P123/L120) using a similar procedure (Fig. 6b–e; Supplementary Fig. S8a, d–f, and Table S2). Both SiCa485 and SiCa675 exhibited excellent photo stability in live cells (Supplementary Fig. S8h, i). Fluorescence imaging of HeLa cells expressing SiCa485 or SiCa675 revealed that histamine-stimulated Ca^2+^ oscillations were associated with increases in the red fluorescence of R-GECO or green fluorescence of GCamP6s, respectively (Fig. 6f, g; Supplementary Videos S5, S6). In particular, SiCa485 could also be used to detect the spontaneous Ca^2+^ oscillations in dissociated neurons (Supplementary Fig. S9). These data suggest that similar to FPs, cpSmFPs also have fluorophores that are sensitive to their local environments, and that it can be used to develop a range of single FP-based sensors to report cellular events, by combinational evolution of the protein and fluorophore.

## Discussion

The SmFPs reported here are similar to FPs from jellyfish or coral in several ways: they are genetically encoded, contain a covalently bound fluorophore, and are intensely fluorescent and highly fluorogenic in response to protein folding and conformational adaptation. By contrast to FPs, however, SmFPs have fast rates of fluorescence generation, allow color manipulation and dynamic protein monitoring by simply switching ligands, providing powerful tools and information that has not been available with traditional pulse-chase methodologies in protein expression, protein–protein interaction, and turnover studies. GFP-like FPs gain their chromophore via an autocatalytic reaction of their chromophore-forming tripeptide. Although clearly successful in their application, genetic engineering to extend the wavelength range of GFP-like FPs has been challenging and time-consuming owing to the limited diversity of natural amino acids and the unpredictable effects of specific mutations of amino acids comprising the FPs’ chromophore, as well as their limited excitation maxima below 610 nm^43^. In contrast, the color of SmFPs’ fluorescence can be fine-tuned rationally and readily from cyan to infrared with current understanding of photochemistry principles largely by manipulating electron donating/accepting capacity of the donor/acceptor, respectively, and the π-conjugation structure within the synthetic fluorophores^44,45^. In the long wavelength region (emission over 640 nm), SmFPs show extraordinarily intense fluorescence with a quantum yield of up to 0.76; this high quantum yield has not been previously demonstrated in the recently developed FPs (Supplementary Table S3)^1^. This unique structural feature would make up for the deficiency of fluorescent proteins in the red fluorescence region. Furthermore, cpSmFPs’ fluorescence is sensitive to the conformational changes of their fusion partners, which is useful for the development of single FP-based GES dynamically responding to various stimuli. During the past decades, cpFPs were successfully used for the development of high-performance GES of large dynamic range. Most of these sensors have emissions in the green range, which was only recently expanded to the blue or red region^42^, and are sensitive to pH variation under physiological conditions. By contrast, SmFPs have different protein folding and fluorescence emission properties extending their fluorescence spectra to the infrared. These unique properties may be exploited for developing new classes of GES with an expanded palette and ‘plug and play’ properties.

The SmFPs presented here are derivatives of SNAP-tag, a self-labeling protein widely used in many systems for a range of applications. Recently, many efforts have been devoted to develop fluorogenic ligands of self-labeling proteins, in order to maximize signal-to-background ratio and alleviate the need of time-consuming washing procedures to remove unbound fluorescent dyes, based on the principle of solvatochromic effects or the mechanism of fluorescence resonance energy transfer destruction^27,46–49^. For example, the strong and stable near-infrared fluorescence of silicon rhodamine (SiR) can be successfully activated after binding to various protein targets. However, these dyes have limited spectral variety or fluorogenicity^50–53^, and may be activated non-specifically under the complex cellular environment similar to other conditional fluorophores^8,27^. Few other works also demonstrated the concept of using a molecular rotor chromophore to covalently bind SNAP-tag for fluorescence labeling^54,55^. However, they suffered from limited fluorescence enhancement and spectral variety (Supplementary Table S5). In contrast, SmFPs possess the same fluorogenic principle as natural FPs, i.e., induction of fluorescence of non-emitting molecular rotors by conformational locking in the binding protein pocket, which enables wide spectral coverage, much better specificity and higher fluorogenicity. The fluorogen-activating proteins^15,16^ and the FAST label also have molecular rotor chromophores of adequate fluorogenicity and low background signals. However, the labeling is non-covalent, precluding chasing of protein dynamics in cells or in vivo^10,17,56^.

Lavis group recently synthesized the “Janelia Fluor” (JF) series dyes and developed a palette of HaloTag-based fluorogenic labels with substantial increases in brightness and photostability^57–62^. Although SmFPs and HaloTag-JF conjugates are both fluorogenic labels, both the self-labeling protein tags and fluorogenic dyes are conceptually different. For the self-labeling protein tags, HaloTag can rapidly and reliably conjugate to probes functionalized with a chloroalkane linker with rapid labeling kinetics and high stability. However, HaloTag is rather large in size (33 kDa)^21^, and fusion of such a large domain may perturb the native function of the protein of interest (POI)^63^. In comparison, SNAP-tag can specifically bind to an *O*^*6*^-benzylguanine equipped ligand with slower kinetics than HaloTag, but the much smaller size offers less overall perturbation of the dynamics and function of the POI. For the fluorogenic dyes, the SmFP dyes belong to the family of molecular rotor, similar to the chromophores of FPs. These molecular rotor type dyes are non-fluorescent in solution owing to rapid intramolecular motion and energy dissipation of the excited state, but fluoresce when these motions are constrained upon protein binding. In comparison, JF dyes exploit the polarity sensitivity of equilibrium between an open, zwitterionic and closed lactone form for rhodamine-based dyes, which allows fluorescence production when the dyes are conjugated to a self-labeling protein tag. Benefit from the high brightness of rhodamine dye and its analogs, HaloTag-JF conjugates outperform SmFPs in the red and far-red spectral regions in term of brightness. However, it is hard to develop rhodamine-based dyes with short wavelength, e.g., cyan and even blue, which significantly limits the spectral range of HaloTag-JF conjugates. In comparison, SmFPs are developed based on the molecular rotor type dyes, which allows to easily expand the spectra according to the general principles of photochemistry and dye design, thus resulting in a palette of SmFPs ranging from the cyan to infrared spectrum (Supplementary Table S5). Taken together, SmFPs and HaloTag-JF conjugates are conceptually different and each has its own unique advantageous properties, which allows combinational usage for multi-color imaging both in live cells and in vivo.

We strongly believe that the advantageous properties of SmFP are attributed to the dendritic molecular rotor fluorophores with rigiid nitrile electron acceptor, which are capable of being locked firmly in the tertiary structure of the protein complex. There is no coincidence that similar dendritic fluorophore was useful for the successful development of highly bright and stable FRs, which significantly outperformed existing fluorescent RNA labeling technologies^9^. In the future, the fluorophores and amino acid sequence of SmFPs may be co-developed to improve further the membrane permeability of the ligand, as well as the fluorescence intensity, stability, ratio of fluorescence signal to background, and red-shifting of florescence. We envision that the chemical-genetic approach to the development of SmFPs described here could be applied to different structured protein domains and molecular rotors, as well, which may extend the evolution of this highly useful technology even further.

## Materials and methods

### Cell culture, transfections, and generation of stable cell lines

HeLa, HEK293, and 293T/17 cells were cultured in DMEM medium, containing 10% FBS in a humidified 5% CO_2_ incubator at 37 °C. Cells were split every 2 d or at confluence. To generate stable cell lines expressing SNAP_f_, cells were infected with appropriate lentivirus at 30%–40% confluence. 48 h after infection, cells were exposed to selective medium containing 1 μg/mL puromycin (Life Technologies), which led to substantial death of non-infected cells over 7–10 d. After amplification of the infected cell population under selective conditions for 4–6 d, the cells were frozen in 10% DMSO and stored at −80 °C.

Unless indicated, transient transfection of HeLa cells was performed using Lipofectamine™ 3000 reagent (Life Technologies) according to the manufacturer’s recommendations: 1 µg of DNA, 2 μL P3000 and 2 μL of Lipofectamine 3000 reagent were each mixed with 50 μL of OptiMEM (Life Technologies). The solutions were incubated for 5 min at room temperature, then mixed and incubated for 15 min at room temperature. The DNA-Lipofectamine complex was added to a 35-mm 4-Chamber glass bottom dishes, with no. 1 cover glasses (In Vitro Scientific), containing HeLa cells at 50%–70% confluence. The cells were incubated for 24 h before imaging. Transient transfection of 293T/17 was performed using FugeneHD reagent (Promega) according to the manufacturer’s recommendations: 0.8 µg of DNA and 2.4 μL of FuGENE® HD Transfection Reagent were mixed and incubated for 15 min at room temperature. The mixture was then added to 35-mm 4-Chamber glass bottom dishes with no. 1 cover glasses (In Vitro Scientific) with 293T/17 cells at 30%–40% confluence.

### DNA cloning

Genes encoding SNAP_f_ tag was cloned into pCDNA3.1/Hygro^(+)^ by *Hind*III/*Xho*I sites to obtain cytosolic expression of SNAP_f_. Mitochondrial-localized SNAP_f_ was constructed by fusing a duplicated mitochondrial targeting sequence derived from the subunit-VIII precursor of human cytochrome c oxidase (Cox-VIII) to the N-terminal end of SNAP_f_. Actin localized SNAP_f_ was obtained by fusing the 17-amino-acid Lifeact sequence to the N-terminal end of SNAP_f_ with an 11-amino-acid linker (LESGGSGGSGS). *Crimson* gene in pEF.MYC, ER-E2-Crimson (Addgene) was replaced by SNAP_f_ using *Sal*I/*Not*I sites to obtain a construct expressing endoplasmic reticulum-localized SNAP_f_. *Turquoise2* gene in pmTurquoise2-Goli (Addgene) was replaced by SNAP_f_ using *Age*I/*Not*I sites to obtain a construct expressing Golgi apparatus-localized SNAP_f_. Lysosome-localized SNAP_f_ was obtained by replacing the *mRFP* gene in pLAMP1-mRFP (Addgene) at *Age*I/*Xba*I sites. The *SNAP*_*f*_ gene was cloned into pDisplay to obtain cell surface-localized SNAP_f_ using *Sal*I/*Bgl*II sites. For nuclear localization, the gene encoding SNAP_f_-H2B was obtained using overlapping PCR and inserted into *Nhe*I/*Hind*III sites of pPAmCherry2-C1 (Addgene) to obtain pSNAP_f_-H2B. *ECFP* gene fragment was inserted into *Nhe*I/*Bgl*II sites to replace SNAP_f_ in pSNAP_f_-H2B to generate pECFP-H2B. The sequence encoding SNAP_f_-mCherry fusion protein was generated using overlapping PCR and inserted into pCDNA3.1/Hygro^(+)^ and pU5(CMV)-Gluc to obtain pCDNA3.1-SNAP_f_-mCherry and pU5(CMV1)-SNAP_f_-mCherry using *Hind*III/*Xho*I sites and *Hind*III/*Bam*HI sites, respectively. The *TIRI* gene was commercially synthesized and inserted into pCDNA3.1/Hygro^(+)^ at *Hind*III/*Xho*I sites. To obtain a construct expressing chimeric fusion protein SNAP_f_-IAA-mCherry-H2B, the *IAA* gene was commercially synthesized and fused to the *mCherry* gene to obtain the IAA-mCherry fusion protein; IAA-mCherry was then inserted into pSNAP_f_-H2B to obtain pSNAP_f_-IAA-mCherry-H2B using the Hieff Clone^TM^ One Step Cloning Kit. *CX43* cDNA was a gift from the Han lab and was fused to SNAP_f_ using overlapping PCR and inserted into pCDNA3.1/Hygro^(+)^ by *Acc*651/*Xho*I sites. To compare the brightness of SmFP485 and cyan FPs in cells, genes encoding SNAP_f_ tag, ECFP, mCerulean3 and mTurquoise2 were cloned into pCDNA3.1/Hygro^(+)^ at *Acc*651 and *Xho*I sites, respectively. To compare fluorescence in live cells, genes encoding SNAP_f_ tag, mCherry, mKate2, iRFP682, and iRFP720 were cloned into pLVX-IRES-ZsGreen at *Eco*RI and *Bam*HI sites, respectively. To monitor protein-protein interaction using spilt-SNAP_f_, we fused 1–74 amino acids of SNAP_f_ to FRB and inserted into pCDNA3.1/Hygro^(+)^ at *Nhe*I/*Xho*I sites; we fused 75–182 amino acids of SNAP_f_ to FKBP and inserted into pCDNA3.1/Hygro^(+)^ at *Hind*III/*BsrG*I sites. To construct the bacterial expression plasmids, genes encoding SNAP_f_, Halo, eDHFR, or ECFP were inserted into pCDFDuet1 vector using *Bam*HI/*Hind*III sites. SNAP_f_ mutations were performed by site-directed mutagenesis according to the MutanBEST protocol (Takara). The DNA fragments encoding CBP-CaM fusion proteins were generated using overlapping PCR and inserted into pCDDFDuet1 at *Bam*HI/*Afl*II sites. Different cpSNAP_f_ truncations were RCR-amplified and inserted into M13-CaM fusions to generate M13-cpSNAP_f_-CaM expression plasmids. Plasmids expressing M13-cpSNAP_f_-CaM variants containing different mutations in cpSNAP_f_ were performed by site-directed mutagenesis according to the MutanBEST protocol (Takara). The genes encoding synthetic indicators for Ca^2+^ were cloned into pCDNA3.1/Hygro^(+)^ at *Nhe*I/*Hind*III sites for measuring Ca^2+^ dynamics in mammalian cells. To compare the photostability of SiCas with FP-based calcium sensors, the genes encoding H2B-SiCa485, H2B-SiCa675, H2B-GCaMP6s, H2B-R-GECO1 and H2B-NIR-GECO1 was obtained using overlapping PCR and inserted into *Nhe*I/*Not*I sites of pEGFP-C1 to obtain pH2B-SiCa485, pH2B-SiCa675, pH2B-GCaMP6s, pH2B-R-GECO, and pH2B-NIR-GECO, using the Hieff Clone^TM^ One Step Cloning Kit.

### In vitro characterization of SNAP_f_

The bacterial expression plasmids of SNAP_f_, Halo-tag, eDHFR, ECFP, and SNAP_f_ mutants were transformed into *E. coli* BL21(DE3) and grown overnight in LB medium containing 50 μg/mL streptomycin. The cultures were diluted 100-fold, and grown at 37 °C to an OD_600_ of 0.4–0.6. Protein expression was induced by 1 mM IPTG for 16 h at 18 °C. Cells were harvested by centrifugation and lysed by sonication. Proteins were purified using an NTA column (GE Healthcare). After washing with two column volumes of wash buffer containing 50 mM imidazole, the proteins were eluted from the resin using Buffer B (30 mM sodium phosphate, 500 mM sodium chloride, and 300 mM imidazole, pH 7.4). The protein preparations were then desalted and exchanged into PBS buffer for in vitro characterization.

Protein concentrations were measured by a BCA protein assay kit (BBI). The excitation and emission spectra of different SmFPs were recorded by incubating 5 μM fluorophore with 5 μM SNAP_f_ using a Cary spectrofluorometer. The relative fluorescence quantum yields of the SmFPs were obtained by comparing the area under the emission spectrum of the sample with fluorescein (which was in 0.1 M NaOH solution with a quantum yield of 0.88 when excited at 460 nm^64^), rhodamine 6G (which was in water solution with a quantum yield of 0.95 when excited at 488 nm^65^), or Cy5 (which was in PBS solution with a quantum yield of 0.27 when excited at 620 nm^66^). A Hitachi U-2000 spectrophotometer was used to determine the absorbance of the SmFPs. Extinction coefficients were calculated according to a Beer–Lambert–Bouguer equation. In order to assess the effect of denaturant on SmFPs, SmFPs were diluted in solutions of GdnHCl with concentrations from 0 M to 6 M; the fluorescence was determined using a Synergy 2 Multi-plate reader (BioTek).

To further calculate the reaction constant k_2_, we applied the pseudo-first-order rate law by setting [fluorophore] ≫ [protein]. We measured the fluorescence of the reaction mixture in which the concentrations of fluorophore varied from 0.5 μM to 4 μM, and the concentration of SNAP_f_ protein was 100 nM. Thereafter, the fluorescence intensity of original data was converted to labeling fraction by the following equation: [Labeled fraction] = (*F*_t_ – *F*_0_)/(*F*_max_ – *F*_0_), where *F*_t_, *F*_0_, and *F*_max_ represent the observed, initial, and maximum fluorescence intensities respectively. By fitting the equation, [Labeled fraction] = 1 – exp(–k_obs_ t), in the plots of labeling fraction vs time, the rate constant (*k*_obs_) of the pseudo-first-order rate equation was obtained and the reaction constant of protein labeling was determined from the slope according to the equation, *k*_2_ = *k*_obs_ [fluorophore].

### Imaging

Cells were plated with antibiotic-free DMEM medium in 35-mm 4-Chamber glass bottom dishes with no. 1 cover glasses (In Vitro Scientific) one day before imaging. Imaging of Ca^2+^ was carried out using a Plan Apo VC 20×, 0.75 numerical aperture (NA) objective and a Prime 95b sCMOS camera on a Ti2 inverted microscope system (Nikon). Other fluorescent imaging for living cells or liver sections was performed using a Leica SP8 confocal laser scanning microscope equipped with HCXPL APO 63.0×/1.47 oil objective and gadolinium hybrid (HyD) detectors.

For studying the synthesis of SmFPs, pU5(CMV1)-SNAP_f_-mCherry and pGAVPO were transfected into HEK293T/17 cells using FugeneHD (Promega) according to the manufacturer’s protocol. Cells were incubated with 2 μM BG-F485 and illuminated with blue light 24 h after transfection. Consecutive imaging of SmFP485 and mCherry fluorescence was recorded. For studying the degradation of SmFP485, HeLa cells were co-transfected with pcDNA-TIR1 and pSNAP_f_-IAA17-mCherry-H2B using Lipofectamine 3000 (Invitrogen). Cells were incubated with 2 µM BG-F485 or 10 µM BG-Fluorescein for 30 min. AID-mediated degradation was performed by adding 500 µM IAA, and consecutive imaging of SmFP485, fluorescein and mCherry fluorescence was recorded. To analyze SmFP485 fragment-based BiFC, HeLa cells were co-transfected with pSNAP_f_ (1–74)-FRB and pSNAP_f_ (75–182)-FKBP. Cells were incubated with 2 µM BG-F485 for 30 min 36 h after transfection. 100 nM rapamycin was then added to induce the interaction of FKBP and FRB. HeLa cells co-transfected with pVenus(1–154)-FRB and pVenus (155–240)-FKBP were used as the controls. For analysis of SmFP485 fragment-based BiFC by FACS, the cells were treated with 100 nM rapamycin for 12 h, and were then incubated with 2 µM BG-F485 for 30 min. The cells were digested and suspended with PBS containing 4% FBS. The fluorescence was analyzed by a Beckman Cytoflex S using a 405/10 nm excitation filter and a 525/40 nm emission filter for SNAP_f_-F485, and a 488/8 nm excitation filter and a 525/40 nm emission filter for Venus.

To compare the fluorescence intensity of SmFP485 and cyan FPs in cells, plasmid encoding SNAP_f_, ECFP, mCerulean3 or mTurquoise2 was transiently transfected into HeLa cells. 48 h after transfection, the cells were imaged using a Leica SP8 confocal microscope with a 405 nm excitation and a 410–500 nm emission. The fluorescence intensities of SmFP485 and cyan FPs were normalized to spectra of SmFP485 and cyan FPs, respectively. To determine the photostability of SmFP485 and cyan FPs, continuous imaging was performed using the same imaging parameters. The curves were normalized to spectral difference of the proteins.

To compare the fluorescence intensity of SmFPs in cells, we transiently transfected live HeLa cells with SNAP_f_-IRES-ZsGreen, mCherry-IRES-ZsGreen, mKate2-IRES-ZsGreen, iRFP682-IRES-ZsGreen, or iRFP720-IRES-ZsGreen. 48 h after transfection, cells were imaged using 561 nm excitation and 570–700 nm emission for mCherry and SmFP615, 600 nm excitation and 610–750 nm emission for mKate2 and SmFP643, 663 nm excitation and 670–770 nm emission for iRFP682 and SmFP680, and 670 nm excitation and 675–790 emission for iRFP720 and SmFP700. For iRFP682 and iRFP720, the cells were incubated with 25 μM BV for 2 h before imaging. Images were processed and analyzed by LAS X software. The fluorescence intensities of SmFPs or FPs were firstly corrected for the spectral differences per FP or SmFP variant, for example, the FPs fluorescence intensities were divided with the relative absorbance at excitation wavelengths; and then were normalized to ZsGreen fluorescence in order to account for variations in transfection efficiency among cells.

For comparison of SmFPs with previously developed fluorogenic ligands for SNAP-tag, HEK293T cells were transiently transfected with plasmid expressing SNAP_f_-tag. Forty-eight hours after transfection, the cells were labeled with different ligands and imaged with a Leica SP8 confocal laser scanning microscope using a 458 nm excitation for CCVJ/SBD/SmFP485/SmFP510 and SmFP520, a 525 nm excitation for SmFP555, a 510 nm excitation for SmFP570, a 555 nm excitation for MaP555, a 625 nm excitation for SmFP643, a 665 nm excitation for SmFP680, a 670 nm excitation for SmFP700 and a 640 nm excitation for SiR, respectively.

For imaging of calcium oscillations, HeLa cells were seeded on PDL-coated 35 mm 4-chamber glass bottom dishes (In Vitro Scientific). 6 h later, 0.2 μg R-GECO (Addgene: 32444) plasmid and 0.4 μg SiCa485 plasmid, or 0.2 μg GCaMP6s plasmid and 0.4 μg SiCa675 plasmid were co-transfected into the HeLa cells using HT. The cells were incubated for 24 h at 37 °C in a CO_2_ incubator. For co-imaging of R-GECO and SiCa485, or GCaMP6s (Addgene: 40753) and SiCa675, cells were incubated with 1 μM BG-F485 or BG-F675 for 60 min. Then, the cells were incubated in HBSS buffer (containing 10 mM HEPES) for imaging. The images were acquired every 3 s for a duration of 10 min, with exposure times ranging between 50 ms and 600 ms (with 2 × 2 binning). Approximately 20 s after the start of the experiment, histamine was added to the chamber with a final concentration of 5 μM. Images were acquired using a Plan Apo VC 20×/0.75 numerical aperture (NA) objective and a Prime 95b sCMOS camera on a Ti2 inverted microscope system (Nikon). For the filters, SiCa485 was imaged with an excitation of 440/10 nm, an emission of 482/30 nm and a 450LP dichroic mirror; R-GECO was imaged with an excitation of 570/40 nm, an emission of 645/70 nm and a 600LP dichroic mirror; SiCa675 was imaged with an excitation of 580/30 nm, an emission of 650LP and a 605LP dichroic mirror; GCaMP6s was imaged with an excitation of 470/40 nm, an emission of 525/50 nm and a 495LP dichroic mirror.

To determine the *K*_*on*_ and *K*_*off*_ rate coefficients for the association and disassociation of calcium to these indicators, the hexahistidine tag-containing SiCa485, SiCa675 or GCaMP6s protein was immobilized onto the Ni-NTA agarose. The disassociation kinetics was measured by recording the fluorescence of the agarose immediately after a EGTA-containing buffer was added to chelate the free calcium in the solution. The curve was fitted to the formula of exponential decay (*y* = *y*_*0*_ + *a* *·* *e*^*–bx*^), where *y* represents the indicator–calcium complex over time, *x* is time, *y*_*0*_ represents the nonspecific binding, *a* is the maximum binding at equilibrium, and *b* is the rate constant. The association rate coefficient (*K*_*on*_) was calculated using the equation *K*_*d*_ = *K*_*off*_*/K*_*on*_.

To determine the photostability of the calcium indicators, HEK293T cells were transiently transfected with plasmid expressing H2B-SiCa485, H2B-GCaMP6s, H2B-SiCa675, H2B-R-GECO or H2B-NIR-GECO. 48 h after transfection, the cells were incubated with 2 µM BG-F485 and 1 µM BG-F675 for SiCa485 and SiCa675, respectively. Continuous imaging of the cells was performed using a Leica SP8 confocal laser scanning microscope with a 470 nm excitation for SiCa485 and GCaMP6s, a 570 nm excitation for SiCa675, R-GECO and NIR-GECO. The curves were normalized to spectrum of each indicator.

For imaging of spontaneous calcium oscillations in dissociated neurons, neurons were dissected and dissociated in papain. The dissociated neurons were plated on PDL-coated 35-mm 4-chamber glass bottom dishes (In Vitro Scientific) for 7 d. The dissociated neurons were transfected with pAAV-RSET-SiCa485 plasmid using lipsome2000 and labeled with 1 μM BG-F485 48 h after transfection. The neurons were then incubated in HBSS buffer (containing 10 mM HEPES) and consecutive imaging of SiCa485 fluorescence was performed using a Plan Apo VC 60×/1.20 numerical aperture (NA) objective and a Prime 95b sCMOS camera on a Ti2 inverted microscope system (Nikon), with an excitation of 440/10 nm and an emission of 482/30 nm and a 450LP dichroic mirror for SiCa485 fluorescence.

### Real-time imaging of protein trafficking with SNAP probe

HeLa cells expressing SNAP_f_-CX43 were seeded in glass bottom 35 mm dish at 90%–100% confluence. 15 h after transfection, cells were incubated with 1 μM BG-F555 in DMEM medium at 37 °C for 30 min. The cells were then washed three times with fresh medium and incubated in DMEM containing 1 μM BG-F485 and 10% FBS. The fluorescence of SmFP485 and SmFP555 was imaged using a Leica SP8 laser scanning confocal microscope equipped with HCXPL APO 63.0×/1.47 oil objective, with a series of confocal slices taken at 0.6 μm intervals, using appropriate filter sets, every 30 min under 37 °C and a CO_2_ atmosphere. The half-life of CX43 was calculated by fitting an exponential function to the fraction of pulse label remaining over time.

HeLa cells expressing Golgi-SNAP_f_, Golgi-mTuquoise2, and LAMP1-RFP were seeded in glass bottom 35-mm dishes at 90%–100% confluence. 24 h after transfection, cells were incubated with 1 μM BG-F555 for 30 min and washed to remove unbound fluorophore. The cells were then incubated with 1 μM BG-F485, and fluorescence of SmFP485 and SmFP555 was imaged for 24 h using a Leica SP8 laser scanning confocal microscope equipped with HCXPL APO 63.0×/1.47 oil objective. To image transient protein trafficking, the cells were first incubated with 1 μM BG-F643 for 0.5 h and washed to remove unbound fluorophore; the cells were then incubated with 1 μM BG-F555 for 0.5 h and washed to remove unbound fluorophore; the cells were lastly incubated with 1 μM BG-F485 and imaged.

HeLa cells expressing SNAP_f_-histone were seeded in glass bottom 35-mm dishes at 90%–100% confluence. 24 h after transfection, cells were incubated with 1 μM BG-F555 for 30 min and washed to remove unbound fluorophore. The cells were then incubated with 1 μM BG-F485, and fluorescence of SmFP485 and SmFP555 was imaged.

HeLa cells expressing mitochondrial-localized SNAP_f_ were seeded in glass bottom 35-mm dishes at 90%–100% confluence. 24 h after transfection, cells were incubated with 1 μM BG-F555 for 1 h and washed to remove unbound fluorophore. The cells were then incubated with 1 μM BG-F485 for 12 h before the fluorescence of SmFP485 and SmFP555 was imaged.

### Chasing of protein expression and in vivo imaging of xenograft tumors in mice

All procedures involving animals were approved by the Institutional Animal Care and Use Committee of Shanghai and were conducted in accordance with the National Research Council Guide for Care and Use of Laboratory Animals. For chasing of protein expression, four-week-old male Chinese Kunming (KM) mice (SLRC Laboratory Animals) of ~20 g body weight were used for in vivo pulse-chase labeling of SNAP_f_ synthesis. 40 µg of pCDNA3.1-SNAP_f_ plasmid was transferred into mice using a hydrodynamic procedure. The mice sequentially received intravenous injections of 40 nmol of BG-F485 and 20 nmol of BG-F643 at 24 h and 58 h, respectively. Mice receiving a single injection of SNAP_f_ plasmid and labeled with BG-F485 and BG-643 simultaneously or sequentially were used as the controls. The livers were dissected from the sacrificed mice using standard surgical procedures. Multispectral images were acquired with an excitation filter of 440/10 nm and an emission filter of 480/50 nm for SmFP485, and an excitation filter of 620/10 nm and an emission filter of 670/50 nm for SmFP643. The SmFP485 and SmFP643 fluorescence was resolved from background fluorescence by CareStream Multispectral program. The livers were then sectioned into 300 μm slices using a Leica VT1200S Vibrating Blade Microtome, and were imaged using a Leica SP8 confocal laser scanning microscope.

For in vivo imaging of xenograft tumors, 4-week-old male ICR mice (Jiesijie Laboratory Animal Co., Ltd) of ~20 g body weight was used for the study. U87-SNAP_f_-expressing cells (1 × 10^7^) suspended in 0.1 mL serum-free RPMI1640 with an equal volume of BD Matrigel Matrix (BD Biosciences, 356237) were inoculated subcutaneously into the mice. When tumor size reached 0.5–1.0 cm in diameter, mice were given intravenous tail vein injection with 400 nmol of the PEG-BG-F700, PEG-BG-F680 or SiR-SNAP suspended in PBS in a total volume of 0.2 mL, and then anesthetized with sodium pentobarbital intraperitoneally (100 mg/kg body weight). Images of SmFP680 and SmFP700 fluorescence were taken by a Kodak Multispectral FX imaging system (Carestream Molecular Imaging) with an excitation of 680/20 nm and an emission of 750/50 nm, or an excitation of 630/20 nm and an emission of 700/50 nm. The SmFP680, SmFP700 and SNAP_f_-SiR fluorescence was resolved from background fluorescence by CareStream Multispectral program. The tumors were then sectioned into 200 μm slices using a Leica VT1200S Vibrating Blade Microtome, and fluorescence images of the cryosections were acquired using a Plan Apo VC 20×/0.75 numerical aperture (NA) objective and a Prime 95b sCMOS camera on a Ti2 inverted microscope system (Nikon), using an Alexa-700 filter (ex: 685/30 nm, em: 730/30 nm).

### Monitoring of protein synthesis in near real time

DNA fragments containing T7 promoter and genes encoding SNAP_f_-mCherry, UnaG, sfGFP, mVenus, mKO2, mTuquoise2, or TagBFP were used as templates to be transcribed into mRNAs using Thermo Scientific Transcript Aid T7 High Yield Transcription Kit. 15 pmol mRNA was expressed in vitro in the cell-free PURE system in the presence of 10 µM BG-F485 for SNAP_f_ or 10 µM biliverdin for UnaG at 37 °C. The fluorescence intensities of the mixture containing SmFP485-mCherry, UnaG, sfGFP, mVenus, mKO2, mTuquoise2, or TagBFP were measured using a 450/10 nm excitation filter and a 485/20 nm emission filter for SmFP485 and mTuquoise2; a 590/20 nm excitation filter and a 645/40 nm emission filter for mCherry; a 485/20 nm excitation filter and a 528/20 emission filter for UnaG, sfGFP, and mVenus; a 400/10 nm excitation filter and a 450/10 nm emission filter for TagBFP; and a 540/25 nm excitation filter and a 590/35 nm emission filter for mKO2 using a Synergy 2 multi-mode microplate reader (BioTek).

### Crystallization, X-ray data collection, structure determination, and refinement

6×His-TEV-SNAP_f_ recombinant protein was first purified using a NTA column (Chelating Sepharose Fast Flow /GE/Cat#17-0575-02). The eluted 6×His-TEV-SNAP_f_ protein was cleaved by the TEV enzyme. The mixture was then purified using a second NTA column (Chelating Sepharose Fast Flow /GE/Cat#17-0575-02) to remove uncleaved protein. The collected protein was dialyzed in 20 mM PBS, 300 mM NaCl, pH 8.0. The purified SNAP_f_ was incubated with a 5-fold excess of BG-F485 for 1 h at 37 °C and then concentrated. The SNAP_f_-BG-F185 was further purified using a gel filtration column (HiLoad 16/60 Superdex75 pre-grade Fast Flow/GE) and concentrated to a final concentration of 17 mg/mL.

Sitting drop and hanging drop methods were used to screen crystallization conditions. A 0.3 μL protein volume and a 0.3 μL buffer volume were mixed. The crystal of SNAP_f_-F485 appeared in the condition of 0.2 M MgCl_2_, 0.1 M Tris-HCl, pH 8.5, 30% (w/v) PEG4000, and grew at 18 °C in the sitting drop. A single crystal was transferred to crystallization buffer with extra 15% glycerol; after 1–5 min, it was flash-frozen by liquid nitrogen. X-ray data were collected for 180° at BL17U, SSRF. Data were processed by HKL20001. The SmFP485 crystal belongs to the P2_1_2_1_2_1_ space group with unit cell parameters of *a* = 69.98 Å, *b* = 90.96 Å, *c* = 52.89 Å, *α* = 90°, *β* = 90°, *γ* = 90° (Supplementary Table S4). The initial structure was determined by the molecular replacement method using a Human *O*^*6*^-alkylguanine-DNA alkyltransferase crystal structure (PDB ID: 3L00) as a search model. Structures were refined further by Coot, Refmac5 and Phenix programs.

### Fluorophore synthesis

Detailed information of fluorophore synthesis is shown in Supplementary information.

### Statistical analysis

For comparison of the fluorescence of SmFPs and FPs in living cells, analysis was performed by a two-tailed Student’s *t*-test (Supplementary Fig. S4). All details on sample size, statistical analysis, mean ± s.d., and *P* value for each experiment are provided in the relevant figure legends.

## Supplementary information

Supplementary information

Supplementary Movie 1

Supplementary Movie 2

Supplementary Movie 3

Supplementary Movie 4

Supplementary Movie 5

Supplementary Movie 6
