# Supplementary material for: Design of a palette of SNAP-tag mimics of fluorescent proteins and their use as cell reporters
Source: Cell Discov. 2023 Jun 13;9:56. doi: 10.1038/s41421-023-00546-y (PMC10264356; doi:10.1038/s41421-023-00546-y)
Supplement: Supplementary file 1 — Supplementary information [file 41421_2023_546_MOESM1_ESM.pdf]

Supplementary information

Design of a Palette of SNAP-tag Mimics of Fluorescent Proteins and Their Use as Cell Reporters

Supplementary Figures

Figure S1: Evolution and Characterization of the SNAP-tag Mimics of Fluorescent Protein
Figure S2: Monitoring Protein Expression, Unfolding, Degradation, and Protein-protein Interaction Using SmFP
Figure S3: Atomic (Crystal) Structure of SmFP Reveals Mechanism of Fluorescence Activation
Figure S4: Fluorogenic Labeling of Subcellular Targeted $SNAP_f$ Fusions
Figure S5: Comparison of SmFPs with Previously Developed Fluorogenic Ligands for SNAP-tag
Figure S6: In vivo Imaging of Xenograft Tumors Using SmFPs
Figure S7: Real-time Monitoring of Protein Expressing, Assembly, and Trafficking Using SmFPs-based Multi-color Pulse-chase
Figure S8: Construction of Synthetic Ca^{2+} Sensor
Figure S9: Imaging of Spontaneous Calcium Oscillations in Dissociated Neurons

Supplementary Tables

Table S1: Photophysical properties of SmFPs
Table S2: Photophysical properties of SiCa and FP-based calcium sensors
Table S3: Photophysical properties of FPs or SmFPs with emission over 640 nm
Table S4: SmFP485 data collection and refinement statistics
Table S5: Photophysical properties of fluorogenic probes for different self-labeling tags

Supplementary Videos

Video S1: Degradation of SmFP485 by AID system
Video S2: Degradation of SNAPf-Fluorescein by AID system
Video S3: Realtime imaging of CX43 synthesis and gap junction assembly
Video S4: Time-lapse imaging of Golgi-SNAPf intracellular trafficking
Video S5: Realtime imaging of the dynamic of cellular calcium using SiCa485
Video S6: Realtime imaging of the dynamic of cellular calcium using SiCa675

Supplementary Notes

Fluorophore synthesis

Supplementary References

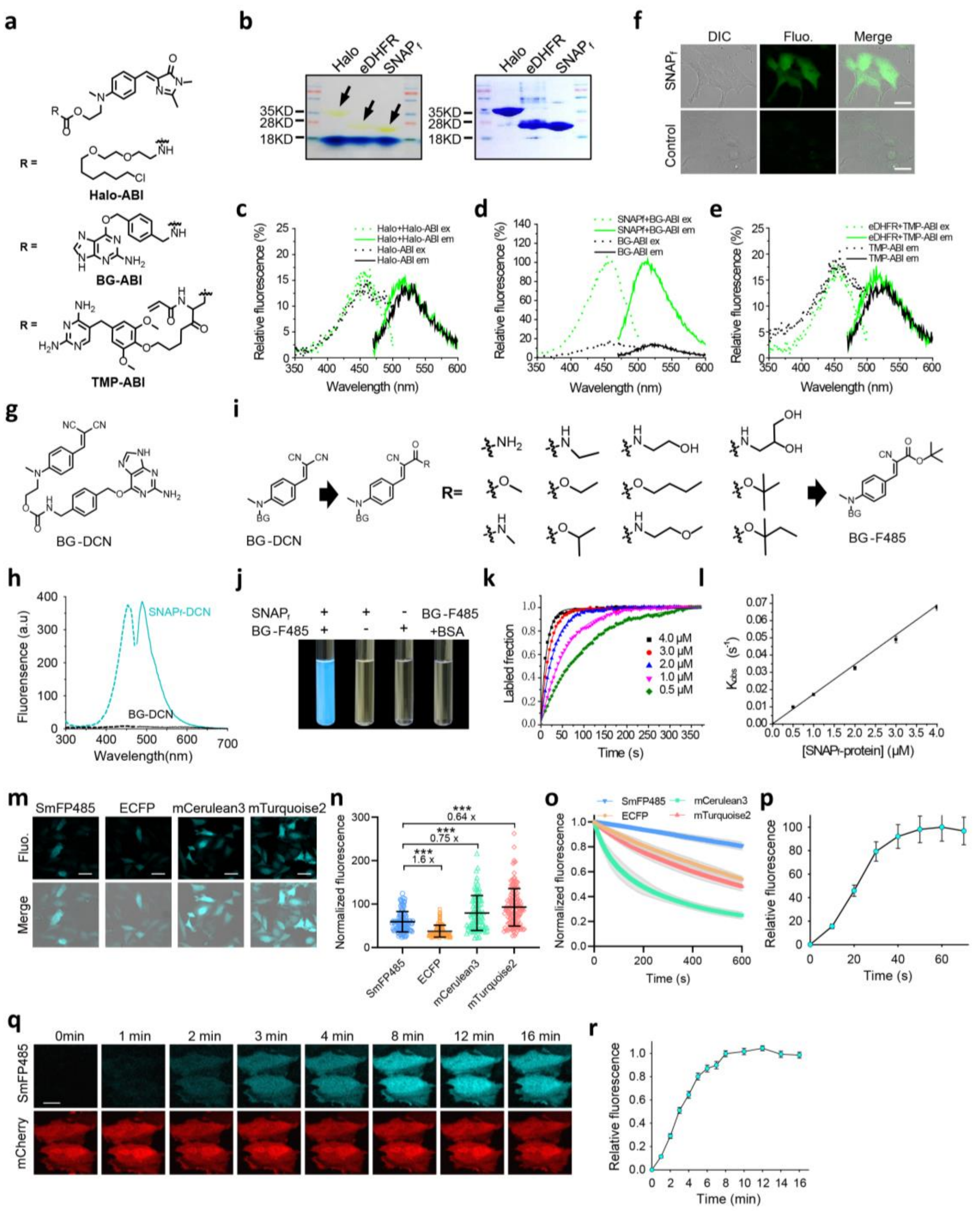

Supplementary Figure S1 Evolution and Characterization of the SNAP-tag Mimics of Fluorescent Protein. (a) Molecular structure of BG-ABI, Halo-ABI, and TMP-ABI. **(b)** SDS PAGE of the covalent complex of ABI-SNAP$_f$, ABI-Halo-tag, and ABI-eDHFR. The yellow color of covalently bound ABI dye is indicated by the arrows. Gel was imaged before (left) and after (right) Coomassie Brilliant Blue staining. **(c)-(e)** Excitation (dashed) and emission (solid) spectra of ABI-SNAP$_f$, ABI-Halo-tag, and ABI-eDHFR covalent complexes. Fluorescence was normalized to ABI-SNAP$_f$ covalent complex. **(f)** Imaging of ABI-SNAP$_f$ fluorescence in live HeLa cells. Scale bars, 20 μm. **(g)** Molecular structure of BG-DCN. **(h)** Excitation (dashed) and

emission (solid) spectra of BG-DCN (black) and DCN-SNAP$_f$ (cyan). **(i)** Molecular structure showing the synthetic evolution of BG-F485 from BG-DCN. **(j)** Fluorescence of BG-F485 is activated by its reaction with SNAP$_f$, but not interaction with BSA. **(k)** Kinetics of SNAP$_f$ protein covalently labeled by BG-F485. Reactions between 100 nM BG-F485 and different concentrations of SNAP$_f$ protein at 37 °C were monitored by the increase of fluorescence over time. Error bars, mean ± SEM. (n = 4). **(l)** Calculated k_{obs} plotted against protein concentration. Error bars, mean ± SEM. (n = 4). **(m)** Comparison of SmFP485 with cyan FPs. HeLa cells transfected with plasmid expressing SNAP$_f$, ECFP, mCerulean3 or mTurquoise2 were imaged using a 405 nm laser excitation and a 410-500 nm emission 48 h after transfection. For imaging of SmFP485, the cells were incubated with 2 μM BG-F485 prior to imaging. Scale bars, 50 μm. **(n)** Quantification of SmFP485, ECFP, mCerulean3 and mTurquoise2 fluorescence in individual cells. The fluorescence was normalized to spectra of SmFP485 and each FP, respectively. Data represent the mean ± s.d. (n = 100 cells). Statistical comparison was performed by two-tailed t-test. ***$P < 0.001$. **(o)** Photostability of SmFP485 and cyan FPs in live cells. HeLa cells (n=20) expressing SNAP$_f$-H2B, ECFP-H2B, mCerulean3-H2B or mTurquoise2-H2B were constitutively imaged using a confocal laser scanning microscope with 405 nm laser. The curves were normalized to spectra difference of the proteins. **(p)** Kinetics of SmFP485 fluorescence generation in cells (n=10) expressing plasma membrane-localized SNAP$_f$. Data related to **Fig. 1e.** **(q)** Consecutive imaging of SmFP485 fluorescence generation in cells expressing SNAP$_f$ incubated with 2 μM BG-F485. Scale bar, 10 μm. **(r)** Kinetics of SmFP485 fluorescence generation in cells (n=10) expressing SNAP$_f$.

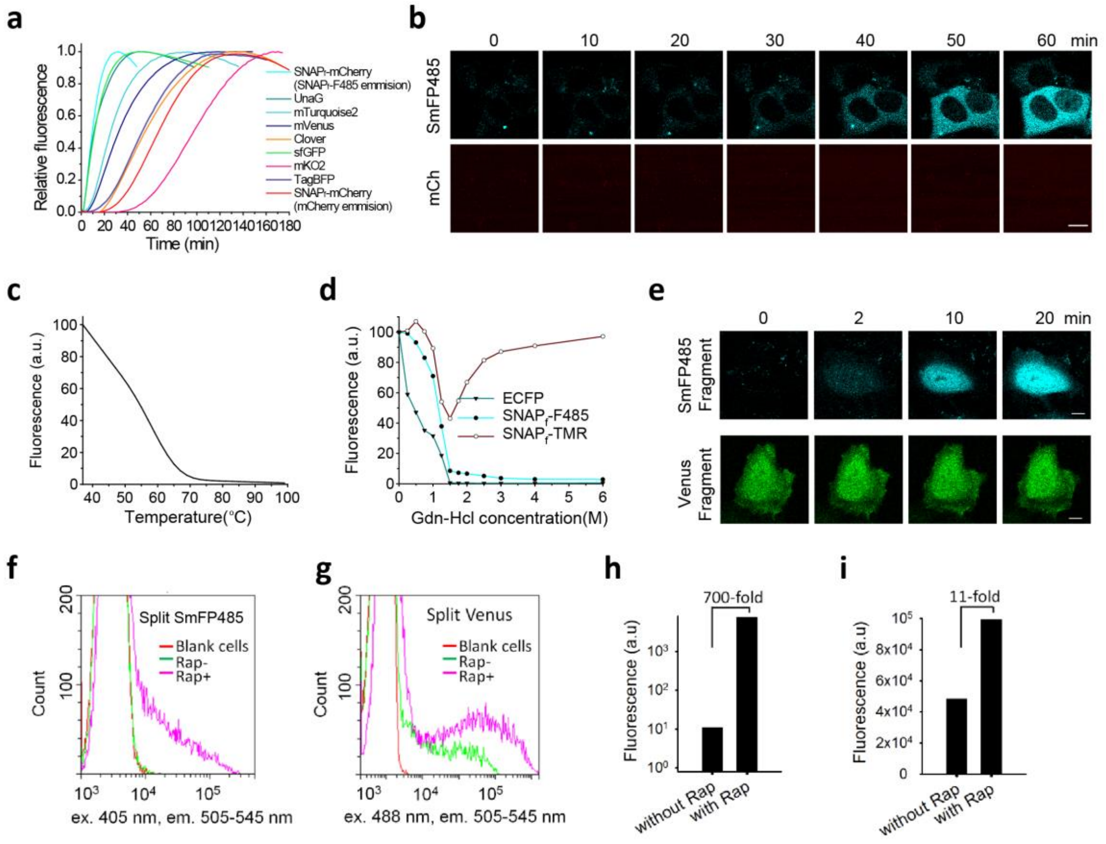

Supplementary Figure S2 Monitoring Protein Expression, Unfolding, Degradation, and Protein-protein Interaction Using SmFP. (a) Fluorescence generation of *in vitro* synthesized SmFP485 and FPs. Fluorescence was monitored immediately after addition of mRNAs of $SNAP_f$ or FPs to the *in vitro* protein expression reaction mixture. **(b)** Brightened (4x) images from **Fig. 2b**. Scale bar, 10 µm. **(c)** SmFP485 fluorescence in response to temperature. **(d)** Fluorescence of SmFP485, ECFP, and $SNAP_f$ covalently labeled with TMR in response to different concentrations of Gdn-HCl. Error bars in **(c)** and **(d)**, mean ± SEM. (n = 3). **(e)** Brightened (12x) images from **Fig. 2j**. Scale bars, 10 µm. **(f)** and **(g)** FACS analysis of BiFC based on $SNAP_f$-fragment and Venus-fragment. **(h)** and **(i)** BiFC contrast upon rapamycin addition for SmFP485-fragment **(h)** and Venus-fragment **(i)**. BiFC contrast is a conventional notion in the field of split reporters that describes the difference between the real, induced signal, and background fluorescence, originated from a nonspecific split reporter complementation [1]. Quantitative data were derived from **(f)** and **(g)**, respectively.

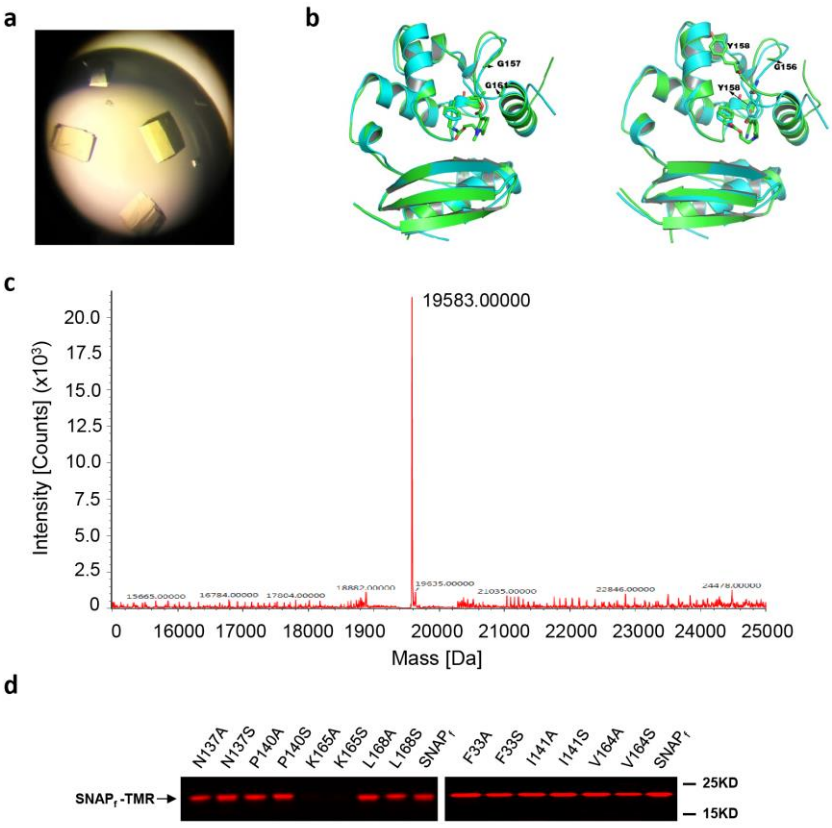

Supplementary Figure S3 Atomic (Crystal) Structure of SmFP Reveals Mechanism of Fluorescence Activation. (a) Crystal of SmFP485. **(b)** Alignment of the structure of SmFP485 (Green, left for subunit 1 and right for subunit 2) and 3L00 (Cyan). Residues in the loop covering the active site are shown. **(c)** LC-MS spectra of SmFP485. A crystal of SmFP485 was selected and dissolved in distilled water, then analyzed by LC-MS. The MW confirmed only a guanine group leaving during the labeling procedure. **(d)** In-gel validation of the capacity for covalent binding of the $SNAP_f$ mutants to BG-TMR. Ten µM protein of $SNAP_f$ mutants was incubated with 50 µM BG-TMR for 1 hr, and was loaded onto SDS PAGE.

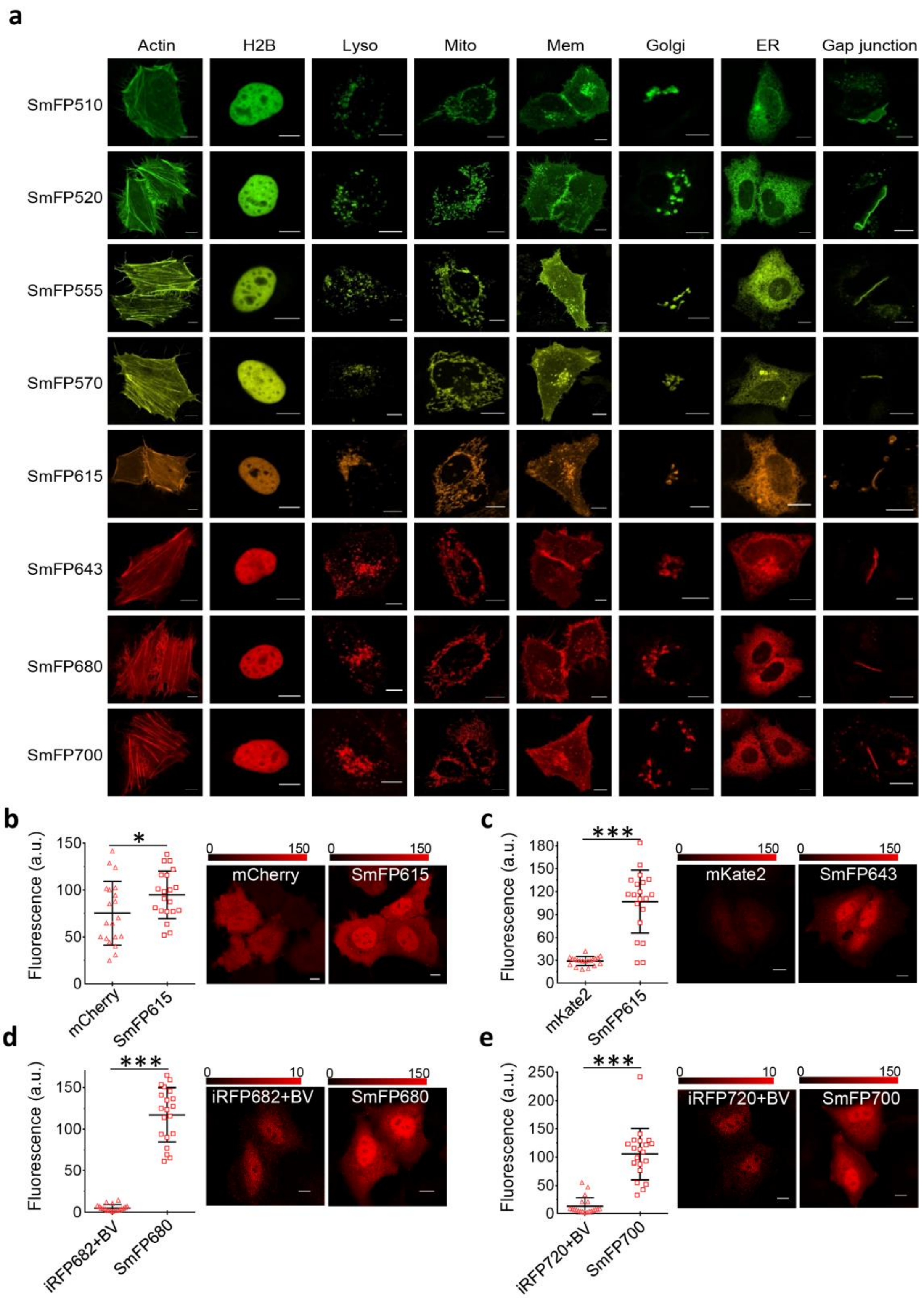

Supplementary Figure S4 Fluorogenic Labeling of Subcellular Targeted SNAP$_f$ Fusions Using SmFPs. (a) Cells expressing different subcellular targeted SNAP$_f$ fusions were labeled with different fluorophores and imaged. Scale bars, 10 µm. **(b)-(e),** Comparison of SmFPs fluorescence with mCherry **(b)**, mKate2 **(c)**, iRFP682 **(d)**, and iRFP720 **(e)** in HeLa cells (n=20).

HeLa cells were transiently transfected with constructs expressing $SNAP_f$-IRES-ZsGreen, mCherry-IRES-ZsGreen, mKate2-IRES-ZsGreen, iRFP682-IRES-ZsGreen, or iRFP720-IRES-ZsGreen. Forty-eight hours after transfection, cells were imaged using 561 nm excitation and 570-700 nm emission for mCherry and SmFP615, 600 nm excitation and 610-750 nm emission for mKate2 and SmFP643, 663 nm excitation and 670-770 nm emission for iRFP682 and SmFP680, and 670 nm excitation and 675-790 emission for iRFP720 and SmFP700. For iRFP682 and iRFP70, the cells were incubated with 25 μM BV for 2 hr before imaging. Fluorescence intensities were firstly corrected for the spectral differences per FP or SmFP variant; and then were normalized to ZsGreen fluorescence in order to account for variations in transfection efficiency among cells. Scale bars, 10 μm. Statistical comparison was performed by two-tailed *t* test, $*P < 0.05$; $***P < 0.001$.

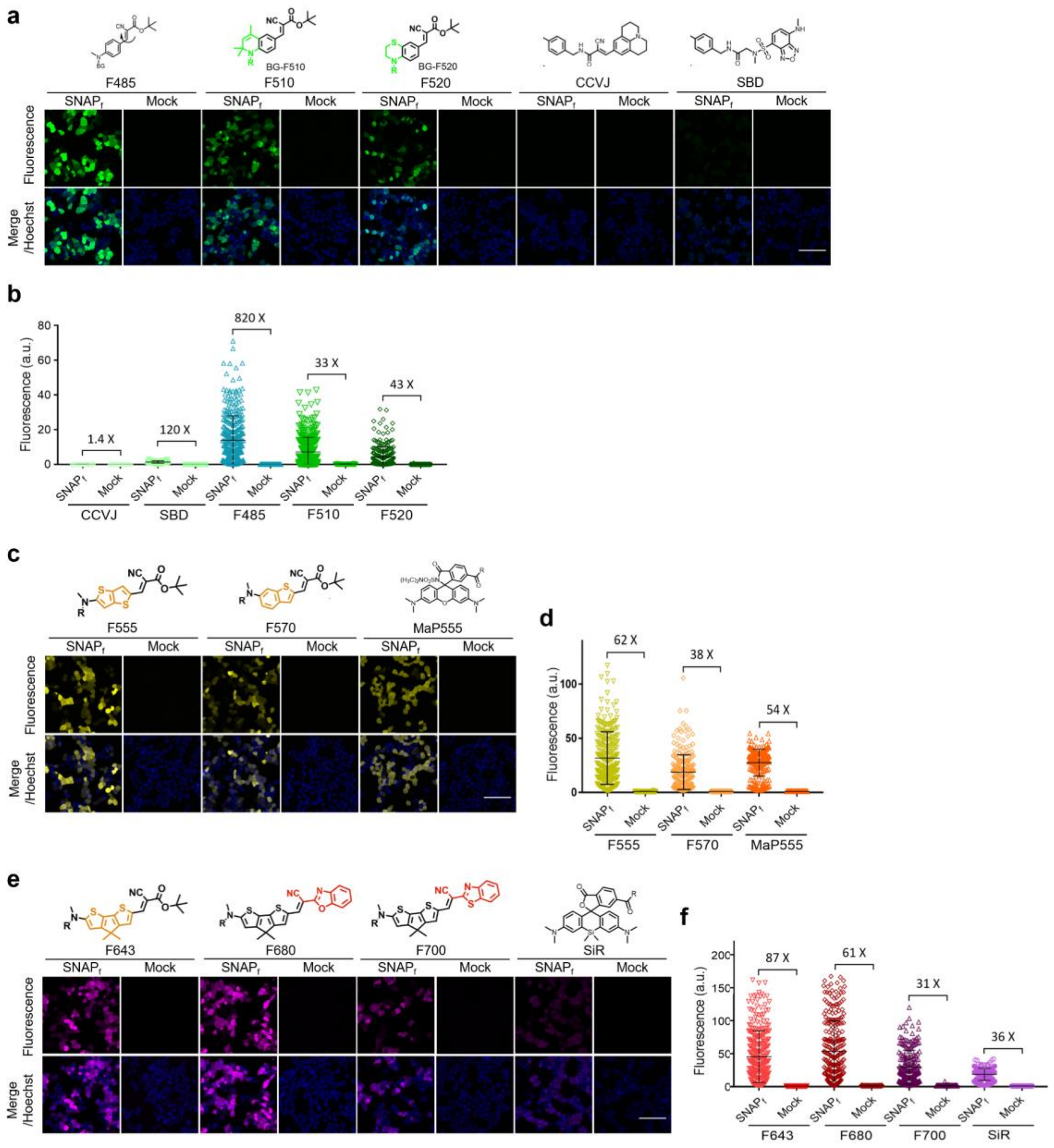

Supplementary Figure S5 Comparison of SmFPs with Previously Developed Fluorogenic Ligands for SNAP-tag. HEK293T cells transfected with plasmid expressing SNAP$_f$ protein were labeled with different ligands 48 hours after transfection. The cells were imaged using a Leica SP8 confocal laser scanning microscope with a 458 nm excitation and a 465-600 nm emission for CCVJ, SBD, SmFP485, SmFP510 and SmFP520 **(a)**, a 525 nm excitation and a 530-650 nm emission for SmFP555, a 510 nm excitation and a 515-650 nm emission for SmFP570, a 555 nm excitation and a 560-650 nm emission for MaP555 **(c)**, a 625 nm excitation and a 630-790

nm emission for SmFP643, a 665 nm excitation and 670-790 nm emission for SmFP680, a 670 nm excitation and a 675-790 nm emission for SmFP700 and a 640 nm excitation and a 645-790 nm emission for SiR **(e)**, respectively. The fluorescence of the cells was quantified **(b, d, f).** HEK293T cells transfected with empty plasmid expressing no $SNAP_f$ protein were used as the controls. Scale bars in **(a)**, **(c)** and **(e)**, 50 µm. Data in **(b)**, **(d)** and **(f)** represent the mean ± s.d. (N ≥ 100 cells)

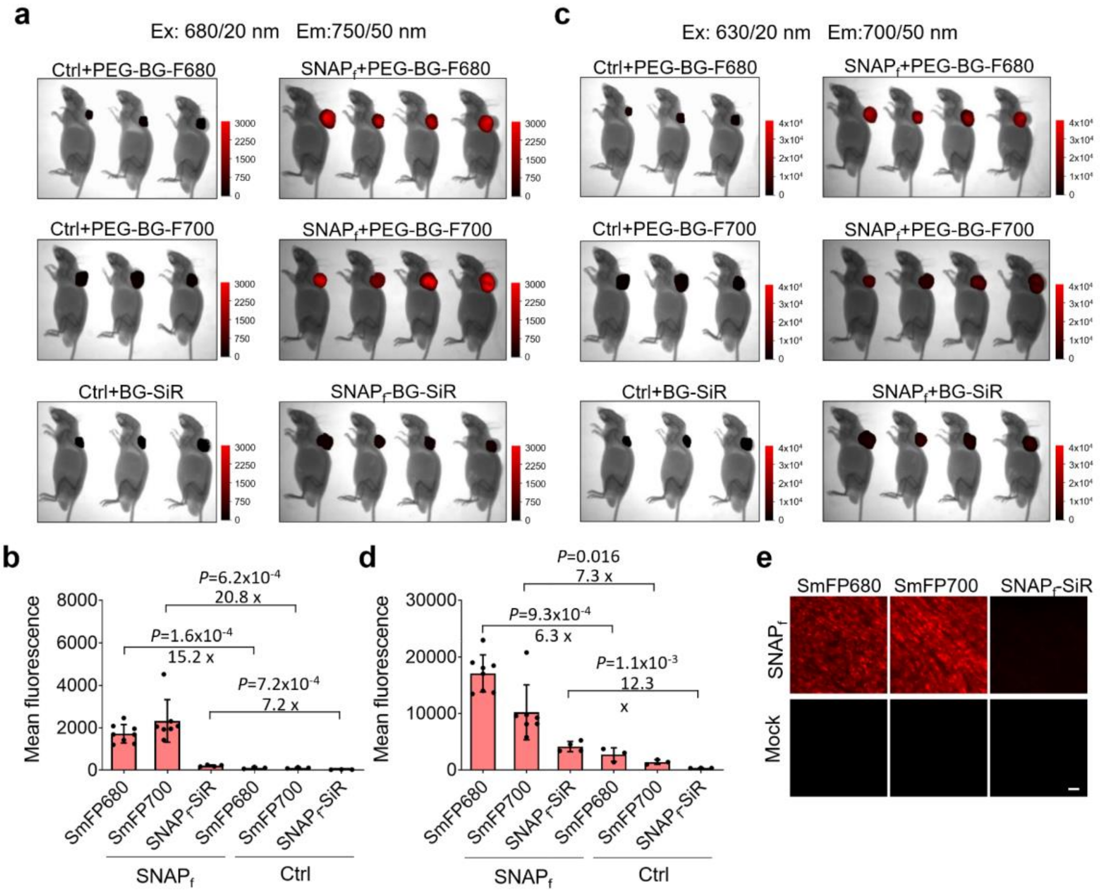

Supplementary Figure S6 In vivo Imaging of Xenograft Tumors Using SmFPs. Xenograft tumors were established using either U87-WT cells or U87-SNAP$_f$ stable cells. In order to increase the hydrophilic of the ligands for *in vivo* labeling, we synthesized PEGylated BG-F680 and BG-F700 that could be bound by SNAP$_f$-tag upon esterlysis by esterase in the blood to remove the PEG groups [2]. 0.4 μmol of the PEG-BG-F700, PEG-BG-F680 or SiR-SNAP was introduced into the mice using intravenous injection. The fluorescence of the tumors was imaged and qualified at 12 h after injection, using an excitation of 680/20 nm and an emission of 750/50 nm **(a** and **b)**, or an excitation of 630/20 nm and an emission of 700/50 nm **(c** and **d)**. Data represent the mean ± S.D. from 3-8 mice. Statistical comparison was carried out by two-tailed *t* test, and the P values were indicated. **(e)** Fluorescence imaging of the tumor sections from the mice in **(a)** using an excitation of 685/30 nm and an emission of 730/30 nm. Scale bar, 100 μm.

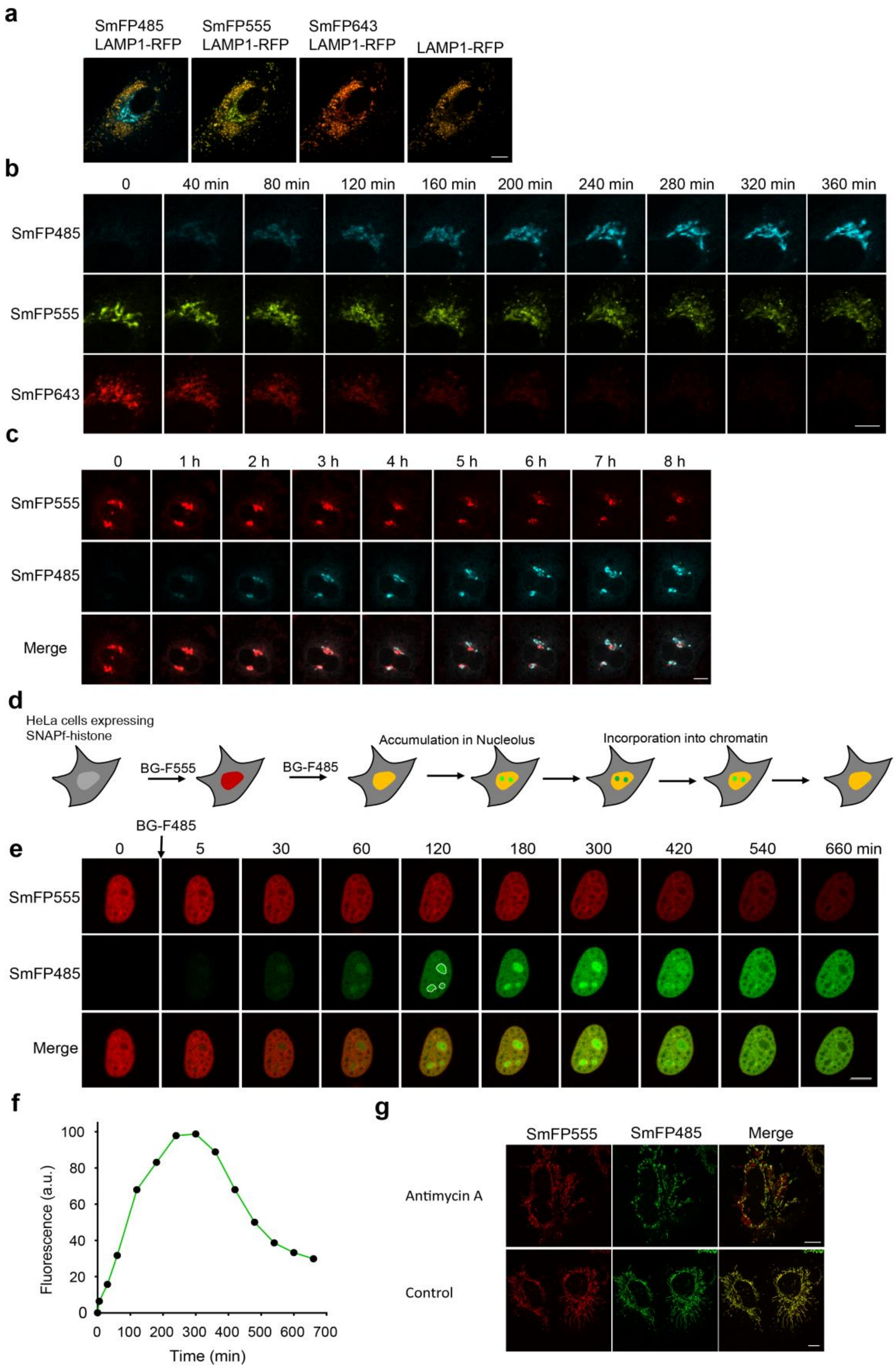

a
SmFP485 LAMP1-RFP
SmFP555 LAMP1-RFP
SmFP643 LAMP1-RFP
LAMP1-RFP
b
0
40 min
80 min
120 min
160 min
200 min
240 min
280 min
320 min
360 min
SmFP485
SmFP555
SmFP643
c
0
1 h
2 h
3 h
4 h
5 h
6 h
7 h
8 h
SmFP555
SmFP485
Merge
d
HeLa cells expressing SNAPf-histone
BG-F555
BG-F485
Accumulation in Nucleolus
Incorporation into chromatin
e
BG-F485
0
5
30
60
120
180
300
420
540
660 min
SmFP555
SmFP485
Merge
f
Fluorescence (a.u.)
Time (min)
0
20
40
60
80
100
0
100
200
300
400
500
600
700
g
SmFP555
SmFP485
Merge
Antimycin A
Control

1

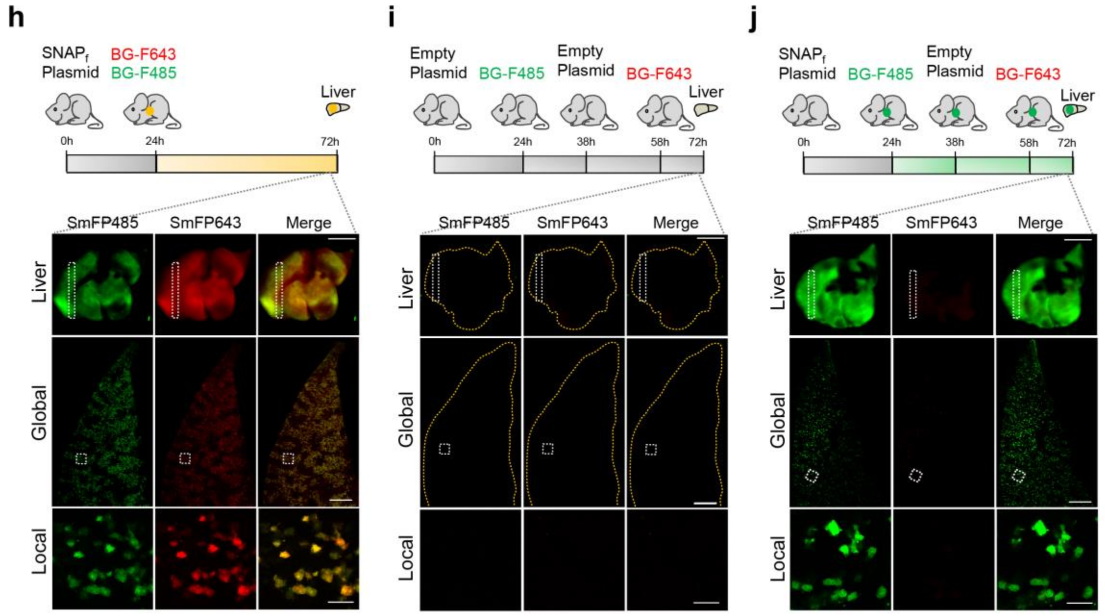

Supplementary Figure S7 Real-time Monitoring of Protein Expressing, Assembly, and Trafficking Using SmFPs-based Multi-color Pulse-chase. (a) Multi-color pulse-chase images of Golgi-SNAP$_f$ trafficking. LAMP1-RFP fluorescence was merged with SmFP485, SmFP555, or SmFP643. Data were from **Fig. 5d**. Scale bar, 10 µm. **(b)** Real-time pulse-chase labeling of Golgi apparatus-localized protein with SmFPs. HeLa cells were co-transfected with pGolgi-SNAP$_f$ and pLamp1-RFP. Twenty-four hours after transfection, cells were labeled with BG-F485 for 30 min. The cells were washed twice with PBS to remove unbound BG-555 and incubated with BG-F555 for 30 min. The cells were next washed twice and labeled with BG-F643, and time-lapse images were performed to monitor the newly synthesized Golgi-SNAP$_f$ and the old Golgi-SNAP$_f$ protein. Scale bar, 10 µm. **(c)** Time-lapse imaging of Golgi-SNAP$_f$ intracellular trafficking. HeLa cells expressing Golgi-SNAP$_f$ were first labeled with BG-F555 for 1 hr, and washed twice with fresh medium. Images were recorded immediately after incubation with BG-F485. Scale bars, 10 µm. **(d)** and **(e)** HeLa cells transfected with SNAP$_f$-histone expressing plasmid were labeled with 1 µM BG-F555 for 30 min 24 hr after transfection. The cells were washed twice with PBS to remove unbound BG-555 and incubated with 1 µM BG-F485. Images were recorded immediately after incubation with BG-F485. Scale bar, 10 µm. **(f)** Quantification of SmFP485 fluorescence of the nucleoli indicated by white circles in **(e)**. Data represent mean fluorescence. **(g)** HeLa cells expressing mitochondrial localized SNAP$_f$ were labeled with 1 µM BG-F555 for 60 min 24 hr after transfection. The cells were washed twice

with PBS to remove unbound BG-555 and incubated with 2 μM antimycin A and 1 μM BG-F485. Cells without treatment of antimycin A were used as the controls. Images were recorded 12 hr after incubation with BG-F485. Scale bars, 10 μm. **(h)-(j)** Imaging of $SNAP_f$ synthesis in mice livers. Fluorescence imaging of livers from mice treated with a single injection of $SNAP_f$ plasmid and simultaneously labeled with BG-F485 and BG-F643 at 24 hr **(h)**, or mice treated without injection of $SNAP_f$ plasmid and sequentially labeled with BG-F485 and BG-F643 at 24 hr and 58 hr, respectively **(i)**, or mice treated with single injection of $SNAP_f$ plasmid and sequentially labeled with BG-F485 and BG-F643 at 24 hr and 58 hr, respectively **(j)**. Scale bars for the liver, global, and local images were 5,000 μm, 1,000 μm, and 100 μm, respectively.

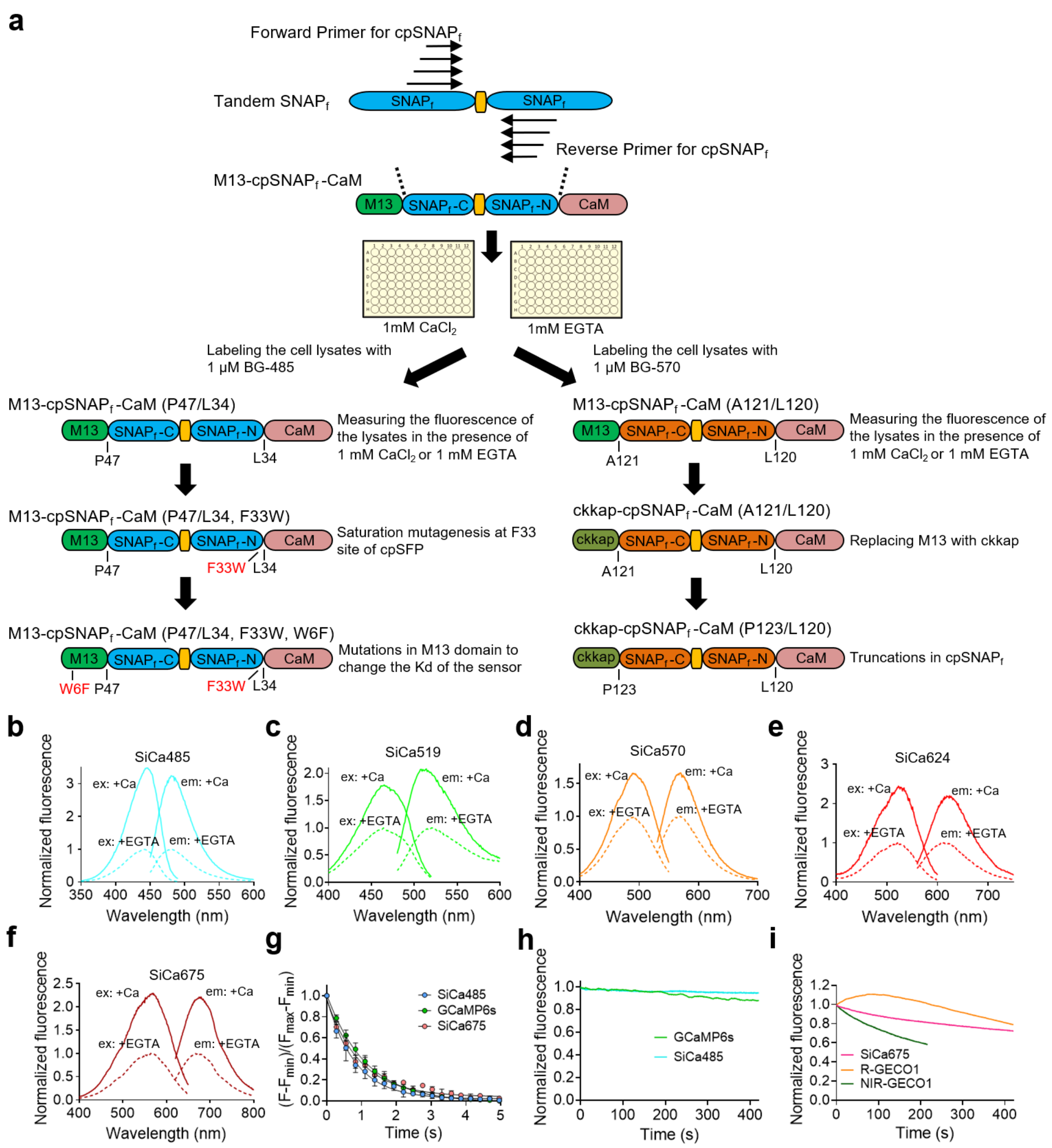

Supplementary Figure S8 Construction of Synthetic Ca^{2+} Sensor. (a) Construction and optimization of Ca^{2+} sensor based on cpSmFP485 and cpSmFP570. We firstly generated the circularly permuted variants of $SNAP_f$ ($cpSNAP_f$) at different loops in $SNAP_f$ protein by linking the original N- and C-termini with a GGGSGGSGGGS flexible linker. We found that $cpSNAP_f$ variants at loop (106-111), loop (136-138) and loop (151-155) were difficult to be expressed. Hence, we chose $cpSNAP_f$ variants at loop (34-50), loop (86-93) and loop (122-126) to construct Ca^{2+} sensor. Different truncated forms of the $cpSNAP_f$ variants were inserted between the M13 peptide and calmodulin (CaM) and cloned into bacterial expression vector. The constructs were transformed into BL21 (DE3) cells and recombinant protein expression were induced at 18 °C for 24 hrs in the presence of 1 mM IPTG. Cells were lysed by sonication

and labeled with 1 μM BG-485 or BG-570, and fluorescence was recorded in the presence of 1 mM $CaCl_2$ or EGTA. M13-cpSNAP$_f$-CaM (P47/L34) variant based on cpSmFP485 and M13-cpSNAP$_f$-CaM (A121/L120) showed the maximal fluorescence enhancement upon addition of Ca^{2+}. For M13-cpSNAP$_f$-CaM (P47/L34), F33 saturation mutagenesis in SNAP$_f$ was carried out to generate the M13-cpSNAP$_f$-CaM (P47/L34, F33W) variant showing the improved fluorescence enhancement upon addition of Ca^{2+}. A W6F mutation in the M13 domain showed a more suitable K_d for measuring the dynamics of intracellular Ca^{2+}. For M13-cpSNAP$_f$-CaM (A121/L120), M13 was replaced with ckkap to generate ckkap-cpSNAP$_f$-CaM (A121/L120). Then, truncations in cpSNAP$_f$ were performed to obtain a sensor showing the maximal fluorescence enhancement upon addition of Ca^{2+}. **(b)**-**(f)** Excitation spectrum (dashed line) and emission spectrum (solid line) of the synthetic indicators of calcium in the Ca^{2+}-free and Ca^{2+}-bound states. **(g)** The kinetics of the disassociation of Ca^{2+} to SiCa485, SiCa675 and GCaMP6s. The hexahistidine tag-containing SiCa485, SiCa675 or GCaMP6s protein was immobilized onto the Ni-NTA agarose. The disassociation kinetics was measured by recording the fluorescence of the agarose immediately after a EGTA-containing buffer was added to chelate the free calcium in the solution. The curve were fitted to the formula of exponential decay ($y = y_0 + a \cdot e^{-bx}$) , where *y* represents the indicator-calcium complex over time, *x* is time, y_0 represents the nonspecific binding, *a* is the maximum binding at equilibrium, and *b* is the rate constant. Data represent the mean fluorescence of four agarose beads. **(h)** The photostability of SiCa485 and GCaMP6s in live cells. HeLa cells expressing H2B-SiCa485 or H2B-GCaMP6s were imaged using a confocal laser scanning microscope with a 470 nm laser excitation. The curves were normalized to spectra of SiCa485 and GCaMP6s, respectively. Data represent the mean fluorescence of 20 cells. **(h)** The photostability of SiCa675, R-GECO1 and NIR-GECO1 in live cells. HeLa cells expressing H2B-SiCa675, or H2B-R-GECO1 and H2B-NIR-GECO1 were imaged using a confocal laser scanning microscope with a 570 nm laser excitation. The curves were normalized to spectra of SiCa675, R-GECO1 and NIR-GECO1, respectively. Data represent the mean fluorescence of 20 cells.

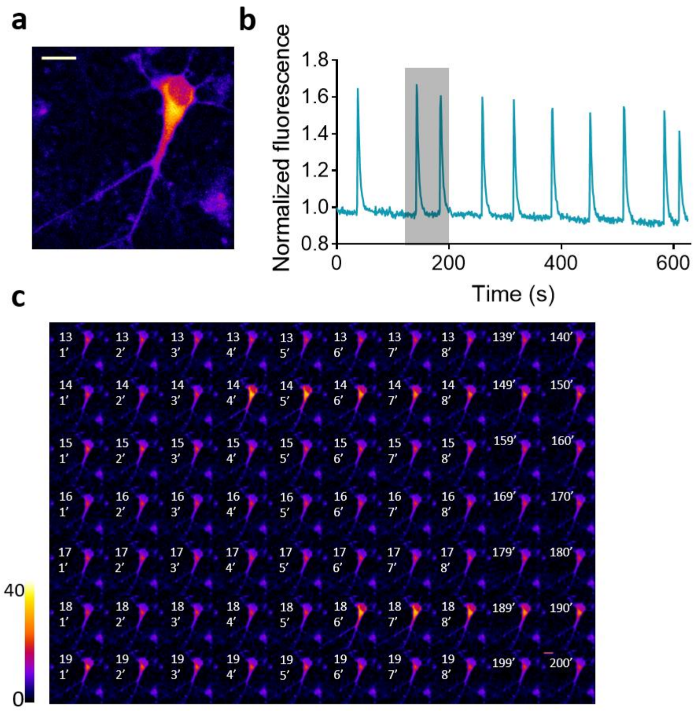

Supplementary Figure S9 Imaging of Spontaneous Calcium Oscillations in Dissociated Neurons. **(a)** Image of dissociated neurons expressing SiCa485. The dissociated neurons were transfected with pAAV-RSET-SiCa485 plasmid and labeled with 1 μM BG-F485 48 h after transfection. The neurons were then incubated in HBSS buffer (containing 10 mM HEPES) and consecutive imaging of SiCa485 fluorescence was performed. **(b)** SiCa485 fluorescence response to spontaneous calcium oscillations in the neuron shown in **(a)**. **(c)** A series of fluorescence images between 130 and 200 seconds in **(b)**. Scale bar, 10 μm.

Supplementary Table S1 Photophysical properties of SmFPs

SmFPs	λ_{ab}(nm)	λ_{Ex}(nm)	λ_{Em}(nm)	ε($M^{-1}cm^{-1}$)	ΦFl	Fluorogenicity (fold)[a]	Brightness[b]	p*K*a
SmFP485	443	443	485	44000	0.36	350	15.8	4.8
SmFP510	459	460	510	35000	0.48	300	16.8	5.8
SmFP519	452	465	519	44000	0.27	279	11.9	5.4
SmFP520	453	462	520	42000	0.35	365	14.7	5.4
SmFP555	526	525	555	68000	0.50	1174	34.0	5.1
SmFP570	497	510	570	65000	0.63	304	41.0	5.4
SmFP615	552	565	615	65000	0.76	589	49.4	5.4
SmFP624	525	535	624	64000	0.25	331	16.0	5.4
SmFP643	625	626	643	69000	0.64	935	44.2	5.2
SmFP675	570	570	675	63000	0.18	486	11.3	5.6
SmFP680	663	665	680	72000	0.51	891	36.7	5.1
SmFP700	666	680	700	78000	0.43	956	33.5	5.0

[a]Fluorescence increase relative to free fluorophore; [b]product of extinction coefficient and quantum yield.

Supplementary Table S2 Photophysical properties of SmFP- and FP-based calcium sensors

SmFPs	λ_{ab}(nm)	λ_{Ex}(nm)	λ_{Em}(nm)	ε($M^{-1}cm^{-1}$)		ΦFl		Dynamic range (F_{max}/F_{min})	Kd (nM)	K_{on}($x10^6$) ($M^{-1}s^{-1}$)	K_{off} (s^{-1})
				$+Ca^{2+}$	$-Ca^{2+}$	$+Ca^{2+}$	$-Ca^{2+}$				
SiCa485	440	446	485	31000	29000	0.27	0.09	3.24	560	2.2	1.2
SiCa519	452	465	519	48000	46000	0.51	0.26	2.08	870	N.D.	N.D.
GCaMP1 [3]	488	487	510	1400	570	0.05	0.03	4.12	235	N.D.	N.D.
GCaMP2 [4]	491	487	508	19000	5200	0.93	0.70	4.85	146	N.D.	N.D.
GCaMP6s [5]	ND	490	510	70117	2118	0.64	0.41	53.8	144	4.3	0.69
jGCaMP7s [5]	ND	495	515	53068	5554	0.65	0.58	40.4	68	21.5	2.87
SiCa570	485	491	570	56000	54000	0.29	0.18	1.67	ND	N.D.	N.D.
SiCa624	510	523	624	75000	67000	0.25	0.13	2.20	710	N.D.	N.D.
SiCa675	551	569	675	59000	48000	0.14	0.08	2.21	710	1.5	1.0
R-GECO1 [6]	ND	561	589	51000	15000	0.20	0.06	16.0	482	N.D.	N.D.
NIR-GECO1 [7]	ND	678	704	20000	62000	0.019	0.063	-9.4	885	N.D.	N.D.
NIR-GECO2 [7]	ND	678	704	18000	67000	0.014	0.059	-15.0	331	N.D.	N.D.

N.D., not determined.

Supplementary Table S3 Photophysical properties of FPs or SmFPs with emission over 640 nm.

FPs or SmFPs	λ_{Ex}(nm)	λ_{Em}(nm)	ε($M^{-1}cm^{-1}$)	Φ_{Fl} (%)	Intensity (relative to EGFP)
EGFP[8]	488	507	56,000	60	100
SmFP643	626	643	69,000	64	131
SmFP680	665	680	72,000	51	109
SmFP700	680	700	78,000	43	100
mPlum[9]	590	649	41,000	10	12
IFP1.4[10]	684	708	92,000	7	19
IFP2.0[11]	690	711	86,000	8	20
mNeptune[12]	600	650	67,000	20	40
NirFP[13]	605	670	15,700	6	3
TagRFP675[14]	598	675	46,000	8	11
TagRFP657[15]	611	657	34,000	10	10
iRFP[16]	690	713	98,000	6	18
iRFP670[16]	643	670	114,000	11	37
iRFP682[16]	663	682	90,000	11	29
iRFP702[16]	673	702	93,000	8	22
iRFP720[16]	702	720	96,000	6	17
mCardinal[17]	604	659	87,000	19	49
mIFP[18]	683	704	82,000	8	20
TDsmURFP[19]	642	670	170,000	18	91
miRFP670[20]	642	670	71,000	12	25
mMaroon1[21]	609	657	80,000	11	26
mGarnet2[22]	598	671	105,000	8.7	27

Supplementary Table S4 SmFP485 data collection and refinement statistics

X-ray data were collected for 180° at BL17U, SSRF. Data were processed by HKL20001. The SmFP485 crystal belongs to space group $P2_12_12_1$ with unit cell parameters of a = 69.98 Å, b = 90.96 Å, c = 52.89 Å, α = 90°, β = 90°, γ = 90°.

Wavelength (Å)	0.97915
Resolution range	90.96-2.09 (2.14-2.09)[a]
Space group	$P\ _{21\ 21\ 21}$
Unit cell	69.98 90.96 52.89 90 90 90
Total reflections	147156
Unique reflections	20618 (1506)
Multiplicity	7.1 (7.3)
Completeness (%)	99.8 (100.0)
Mean I/sigma(I)	18.6 (2.2)
R-merge (%)	7.6 (110.7)
Reflections used in refinement	20565
Reflections used for R-free	1051
R-work/R-free	0.2157/0.2682
Number of non-hydrogen atoms	2424
macromolecules	2258
ligands	64
Protein residues	309
RMS(bonds)	0.014
RMS(angles)	1.160
Ramachandran favored (%)	98.61
Ramachandran allowed (%)	3.05
Ramachandran outliers (%)	0.34
Average B-factor	45.69
macromolecules	44.99
ligands	68.71
solvent	46.65

[a]Statistics for the highest-resolution shell are shown in parentheses.

Supplementary Table S5 Photophysical properties of fluorogenic probes for different self-labeling tags

Dye	Protein	λ_{ab}(nm)	λ_{Ex}(nm)	λ_{Em}(nm)	ε($M^{-1}cm^{-1}$)	ΦFl	Fluorogenicity (fold)[a]	Reference
BG-MR 121	SNAP-tag	660	N.D.	675	N.D.	0.3	17.8	Ref [23]
BG-ATTO 655	SNAP-tag	663	N.D.	684	N.D.	0.3	13.5	
BG-ATTO 680	SNAP-tag	680	N.D.	700	35000	0.3	28.0	
BG-ATTO 700	SNAP-tag	700	N.D.	719	44000	0.25	31.6	
DRBG-488	SNA-Ptag	N.D.	490	525	N.D.	N.D.	300	Ref [24]
CBG-488-TQ2	SNAP-tag	N.D.	490	525	N.D.	N.D.	76.9	Ref [25]
CBG-549-QSY7	SNAP-tag	N.D.	560	575	N.D.	N.D.	62.5	
MaP555	SNAPtag	556	N.D.	576	54000	0.46	21	Ref [26]
BG-SBD	SNAP-tag	435	N.D.	516	13000	0.143	280	Ref [27]
BG-CCVJ	MGMT	N.D.	N.D.	504	N.D.	N.D.	170	Ref [28]
4c	SNAP-tag	534	530	623	24768	N.D.	90	Ref [29]
3	HaloTag	533	530	630	39049	0.22	N.D.	Ref [30]
D1-HTL	HaloTag	N.D.	N.D.	508	14850	0.23	200	Ref [31]
D2-HTL	HaloTag	N.D.	N.D.	508	11550	0.14	150	
P4	HaloTag	423	N.D.	545	4100	0.16	100	Ref [32]
P8	HaloTag	440	N.D.	535	7800	0.37	600	
P9	HaloTag	450	N.D.	530	5100	0.47	1000	
CCVJ-Halo	HaloTag	458	N.D.	498	31800	0.014	15	Ref [33]
Y-Halo	HaloTag	489	N.D.	562	34900	0.015	12	

Orange-Halo	HaloTag	487	N.D.	574	16200	0.02	48	
Red-Halo2-PEG	HaloTag	504	N.D.	588	14500	0.05	32	
Red-Halo2	HaloTag	515	N.D.	592	21000	0.17	156	
NIR-Halo1	HaloTag	526	N.D.	671	25800	0.053	130	
SiR-SNAP	SNAPtag	650	N.D.	668	~100000	0.30	N.D.	Ref [34]
SiR-CLIP	CLIPtag	652	N.D.	668	~100000	0.46	N.D.	
SiR-Halo	HaloTag	648	N.D.	668	~100000	0.39	N.D.	
520R	HaloTag	521	N.D.	546	52000	0.79	N.D.	Ref [35]
580R	HaloTag	581	N.D.	607	58000	0.95	N.D.	
610CP	Halotag	609	N.D.	634	100000	0.59	N.D.	
JF_{503}	HaloTag	503	N.D.	529	83000	0.87	N.D.	Ref [36]
JF_{519}	HaloTag	519	N.D.	546	59000	0.85	N.D.	
JF_{525}	HaloTag	525	N.D.	549	94000	0.91	N.D.	
JF_{549}	HaloTag	549	N.D.	571	101000	0.88	N.D.	
JF_{585}	HaloTag	585	N.D.	609	1500	0.78	N.D.	
JF_{608}	HaloTag	608	N.D.	631	99000	0.67	N.D.	
JF_{635}	HaloTag	635	N.D.	652	~400	0.56	N.D.	
JF_{646}	HaloTag	646	N.D.	664	5000	0.54	N.D.	
SiRcB	BL-tag	654	N.D.	671	100000	0.39	135	Ref [37]
SiRcB2	BL-tag	651	N.D.	670	100000	0.38	250	
SiRcB4	BL-tag	651	N.D.	670	100000	0.39	345	

SiRcB6	BL-tag	651	N.D.	670	100000	0.37	350	
P1	AgHalo	440	N.D.	545	N.D.	N.D.	~50 (59℃, 30 min)	Ref [38]
P2	AgHalo	440	N.D.	495	N.D.	N.D.	~112 (59℃, 30 min)	Ref [39]
MaP510	HaloTag	510	N.D.	531	61000	0.77	18	Ref [26]
MaP555	HaloTag	558	N.D.	578	87000	0.54	35	
MaP618	HaloTag	618	N.D.	635	107000	0.61	1000	
MaP700	HaloTag	700	N.D.	720	52000	0.24	650	
JF_{669}	HaloTag	669	N.D.	682	112000	0.37	N.D.	Ref [40]
JF_{690}	HaloTag	690	N.D.	707	150000	0.24	N.D.	
JF_{711}	HaloTag	711	N.D.	732	12400	0.17	N.D.	
JF_{722}	HaloTag	722	N.D.	743	87200	0.11	N.D.	
JF_{724}	HaloTag	724	N.D.	748	6600	0.05	N.D.	
JFX_{646}	HaloTag	645	N.D.	662	8600	0.73	N.D.	Ref [41]
JFX_{650}	HaloTag	650	N.D.	667	17600	0.53	N.D.	
TMP455	DHFR-Tag	N.D.	380	455	34000	0.78	650	Ref [42]
TMP465	DHFR-Tag	N.D.	403	465	35000	0.50	1125	
TMP485	DHFR-Tag	N.D.	415	485	35000	0.49	4000	
TMP525	DHFR-Tag	N.D.	512	522	60000	0.65	1600	

N.D., not determined

Supplementary Note Fluorophore synthesis

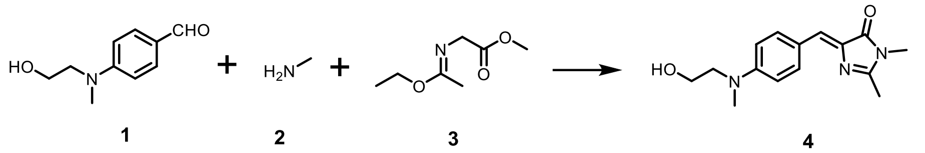

Compound 4: To a stirred solution of 4-((2-Hydroxyethyl)(methyl)amino)benzaldehyde (compound 1) (0.90 g, 5.0 mmol) and 5 mL 33% methylamine (compound 2) methanol solution in 50 mL anhydrous methanol, 10 g Na_2SO_4 was added in one portion. The obtained mixture was stirred and kept at room temperature for 24 hr, then filtered and dried with additional Na_2SO_4. The solvent was removed under reduce pressure to give the intermediate which was used directly without any further purification. After re-dissolved in 10 mL anhydrous methanol, ethyl((1-methoxy)amino)acetate (compound 3) [43] (0.95 g, 6 mmol) was added. The complex was stirred and kept at room temperature for 12 hr, the precipitated product was filtered and washed with cooled methanol to give the yellow compound 4. (1.02 g, yield 75%). ^1H NMR (400 MHz, DMSO-d_6): δ=8.03 (d, J=8.8 Hz, 2 H), 6.85 (s, 1 H), 6.74 (d, J=8.8 Hz, 2 H), 4.74(t, J=5.6 Hz, 1 H), 3.56(t, J=6.0 Hz, 2 H), 3.48(t, J=6.0 Hz, 2 H), 3.07(s, 3 H), 3.01 (s, 3 H), 2.31 (s, 3 H). ^{13}H NMR (100 MHz, DMSO-d_6): δ= 169.57, 160.11, 150.33, 134.37, 133.78, 126.35, 121.16, 111.30, 58.09, 53.75, 38.64, 26.05, 15.13. MS(ESI): m/z Calcd. For $C_{15}H_{19}N_3O_2$ 273.3; found 274.3, $[M+H]^+$.

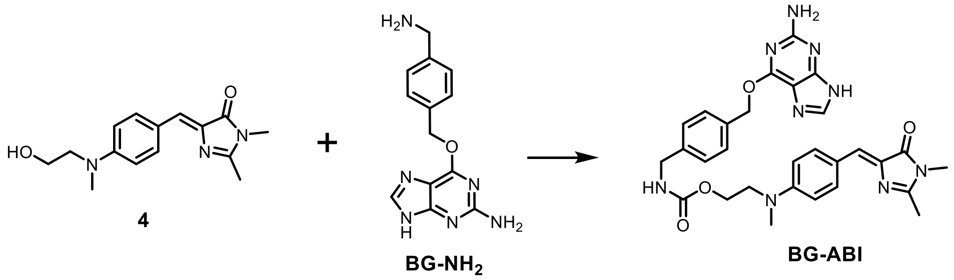

Compound BG-ABI: To a stirred solution of compound 4 (0.273 g, 1.0 mmol) and 4-dimethylaminopyridine (DMAP) (0.147 g, 1.2 mmol) in 20 mL dry CH_2Cl_2, 4-nitrophenylchloroformate (0.302 g, 1.5 mmol) in 10 mL dry CH_2Cl_2 was added dropwise. The obtained mixture was stirred and kept at room temperature for 2 hr. The solvent was removed under reduced pressure and the obtained intermediate was used directly without any further purification. After re-dissolved in dry dimethylformamide (DMF), BG-NH_2 [44] (0.324 g, 1.2 mmol) and 0.2 mL TEA were added. The obtained mixture was stirred at room temperature for another 1 hr. The solvent was removed under reduced pressure to give the crude product which was purified by silica gel chromophore to give the yellow BG-HBI. (0.512 g, yield 90%).

^{1}H NMR (400 MHz, DMSO-d_6): δ = 8.05 (d, J = 8.8 Hz, 2H), 7.95 (s, 1H), 7.82 (s, 1H), 7.74 (t, J = 6.0 Hz, 1H), 7.43 (d, J = 8.0 Hz, 2H), 7.22 (d, J = 8.0 Hz, 2H), 6.86 (s, 1H), 6.78 (d, J = 8.8 Hz, 2H), 6.29 (s, 2H), 5.42 (s, 3H), 4.19 – 4.12 (m, 4H), 3.64 (t, J = 5.6 Hz, 2H), 2.88 (s, 3H), 2.72 (s, 3H), 2.30 (s, 3H). ^{13}H NMR (100 MHz, DMSO-d_6): δ= 169.65, 162.29, 160.41, 159.59, 156.31, 156.21, 150.58, 150.15, 139.54, 136.61, 135.28, 135.13, 134.65, 133.86, 128.53, 126.95, 126.31, 121.64, 121.31, 111.46, 111.06, 66.53, 61.02, 51.95, 50.38, 45.42, 43.50, 40.39, 38.33, 35.74, 30.72, 26.07, 15.13, 14.69, 8.86, 7.13. HR-MS (ESI): m/z Calcd. For $C_{29}H_{31}N_9O_4$ 569.2499; found 589.2497, $[M+Na]^+$.

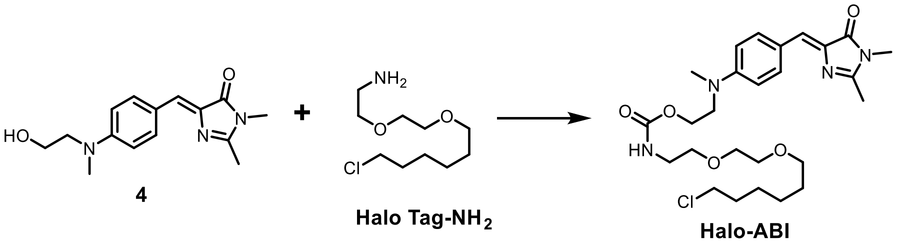

Compound Halo-ABI: To a stirred solution of compound 4 (0.273 g, 1.0 mmol) and DMAP (0.147 g, 1.2 mmol) in 20 mL dry CH_2Cl_2, 4-nitrophenylchloroformate (0.302 g, 1.5 mmol) in 10 mL dry CH_2Cl_2 was added dropwise. The obtained mixture was stirred and kept at room temperature for 2 hr, then Halo Tag-NH_2 [45] (0.368 g, 1.2 mmol) in 5 mL dry CH_2Cl_2 was added. The mixture was stirred at room temperature for another 30 min. The solvent was removed under reduce pressure to give the crude product which was purified with gel silica gel column chromatography to afford the yellow Halo-HBI. (0.450 g, yield 86%). ^{1}H NMR (400 MHz, DMSO-d_6): δ= 8.05 (d, J=8.8 Hz, 2 H), 7.18(t, J=5.6 Hz, 1 H), 6.86(s, 1 H), 6.77 (d, J=8.8 Hz, 2 H), 4.11 (t, J=5.6 Hz, 2 H), 3.59-3.63(m, 4 H), 3.40-3.47 (m, 4 H), 3.30-3.38 (m, 2 H), 3.07-3.11 (m, 5 H), 3.00 (s, 3 H), 2.31 (s, 3 H), 1.65-1.72 (m, 2 H), 1.44-1.50 (m, 2 H), 1.19-1.40 (m, 6 H). ^{13}H NMR (100 MHz, DMSO-d_6): δ= 169.38, 160.16, 155.86, 149.85, 134.43, 133.60, 126.01, 121.41, 111.21, 69.89, 69.26, 69.13, 68.80, 60.64, 50.14, 45.89, 38.17, 31.73, 28.77, 25.84, 24.63, 14.93. HR-MS (ESI):m/z Calcd. For $C_{26}H_{39}ClN_4O_5$ 522.2609; found 523.2605, $[M+H]^+$.

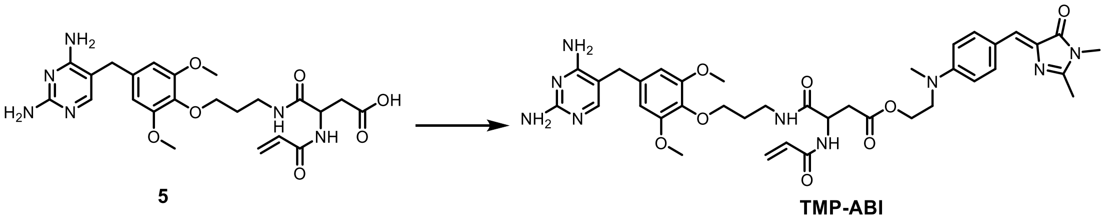

Compound TMP-ABI: To a stirred solution of compound 4 (0.328 g, 1.2 mmol), DMAP (73.2 mg, 0.6 mmol) and 1-ethyl-3-(3-dimethylaminopropyl) carbodiimide (EDCI) (0.115 g, 0.6 mmol) in 5 mL anhydrous DMF, compound 5[46] (0.256 g, 5 mmol) was added and kept at room

temperature. The obtained mixture was stirred for 1 hr. The solvent was removed under reduce pressure to give the crude product which was purified by silica gel flash chromatography to give the TMP-ABI. (0.31 g, 81%). ^{1}H NMR (400 MHz, DMSO-d_6): δ= 8.03 (d, J=8.8 Hz, 2 H), 7.25 (s, 2 H), 6.85 (s, 1 H), 6.74 (d, J=8.8 Hz, 2 H), 6.57 (s, 2 H), 6.24 (dd, J=16.8 Hz, 9.0 Hz, 1 H), 6.16 (dd, J=16.8 Hz, 9.0 Hz, 1 H), 5.61 (dd, J=8.8 Hz, 3.0 Hz, 1 H), 4.8 (m, 1 H), 4.74 (t, J=5.6 Hz, 1 H), 3.98 (m, 2 H), 3.82 (s, 6 H), 3.56(t, J=6.0 Hz, 2 H), 3.48(t, J=6.0 Hz, 2 H), 3.46 (t, J=6.0 Hz, 2 H), 3.07 (s, 3 H), 3.01 (s, 3 H), 2.31 (s, 3 H), 2.87 (dd, J=16.8 Hz, 6 Hz, 1 H), 2.72 (dd, J=16.8, 7.2 Hz, 1 H), 1.88 (m, 2 H). HR-MS (ESI): m/z Calcd. For $C_{38}H_{47}N_9O_8$ 757.3548; found 780.3550, $[M+Na]^+$.

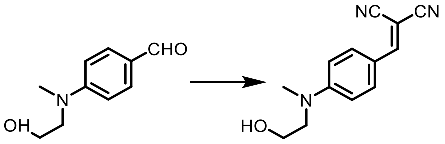

6

Compound 6: To a stirred solution of 4-((2-Hydroxyethyl) (methyl) amino) benzaldehyde (0.18 g, 1.0 mmol) and malononitrile (0.079 g, 1.2 mmol) in 10 mL anhydrous methanol, a catalytic amount (2 drops) of piperidine was added. The obtained mixture was stirred and kept at 80 °C for 15 min under protection of N_2. After cooling to room temperature, the solvent was removed under reduce pressure to give the crude product which was purified by silica gel column chromatography to afford the yellow compound 6. (0.22 g, yield 98%). ^{1}H NMR (400 MHz, DMSO-d_6): δ= 8.08 (s, 1H), 7.96 (s, 1H), 7.84 (d, J = 8.8 Hz, 2H), 7.76 (t, J = 6.2 Hz, 3H), 7.44 (d, J = 8.0 Hz, 2H), 7.21 (d, J = 8.0 Hz, 2H), 6.91 (d, J = 9.2 Hz, 2H), 5.46 (s, 1H), 4.22-4.11 (m, 2H), 3.74 (t, J = 5.4 Hz, 2H), 3.09 (s, 3H). ^{13}H NMR (100 MHz, DMSO-d_6): δ= 158.87, 156.12, 153.73, 139.52, 134.87, 133.55, 118.97, 116.10, 115.36, 68.92, 66.86, 61.01, 50.55, 43.52, 40.35, 38.69. MS(ESI): m/z Calcd. For $C_{13}H_{13}N_3O$ 227.1; found 228.1, $[M+H]^+$.

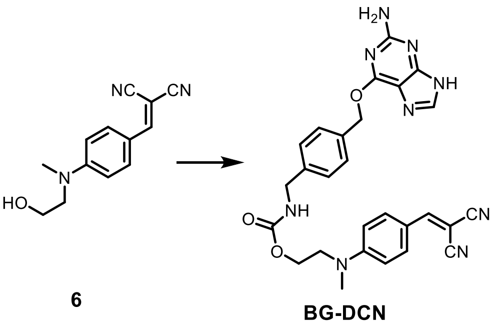

6 BG-DCN

Compound BG-DCN: 4-nitrophenylchloroformate (0.302 g, 1.5 mmol) in 10 mL dry CH_2Cl_2 was added dropwise to a solution of compound 6 (0.227 g, 1.0 mmol) and 4-dimethylaminopyridine (0.147 g, 1.2 mmol) in 20 mL dry CH_2Cl_2. The obtained mixture was

stirred and kept at room temperature for 2 hr under the protection of N_2. The solvent was removed under reduce pressure to give the intermediate which was used directly without any further purification. After re-dissolved in dry DMF, BG-NH_2 (0.324 g, 1.2 mmol) was added in the presence of 0.2 mL TEA. The obtained mixture was stirred and kept at room temperature for 1 hr under the protection of N_2. The solvents were removed under reduce pressure to give the crude product which was purified with gel silica gel column chromatography to give the yellow BG-DCN. (0.465 g, yield 89%). ^{1}H NMR (400 MHz, DMSO-d_6): δ= 8.08 (s, 1 H), 7.85 (m, 3 H), 7.82(t, J=6.0 Hz, 1 H), 7.43 (d, J=8.0 Hz, 2 H), 7.21(d, J=8.0 Hz, 2 H), 6.91 (d, J=8.8 Hz, 2 H), 6.31 (s, 2 H), 5.45 (s, 2 H), 4.12 (t, J=5.6 Hz, 2 H), 3.74 (t, J=5.6 H, 2 H), 3.08 (s, 3 H). ^{13}C NMR (100 MHz, DMSO-d_6): δ= 159.54, 158.90, 156.19, 153.76, 139.50, 135.16, 133.65, 128.50, 127.00, 126.16, 119.01, 116.17, 115.77, 115.44, 111.90, 68.99, 66.56, 60.97, 55.99, 50.55, 45.54, 43.52, 38.68, 18.52. HR-MS(ESI):m/z Calcd. For $C_{27}H_{25}N_9O_3$ 523.2080; found 546.2083, [M+Na]+.

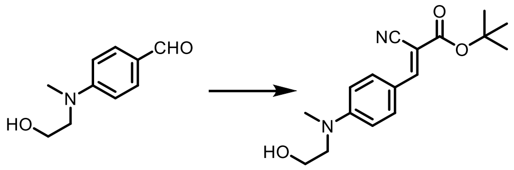

7

Compound 7: To a stirred solution of 4-((2-Hydroxyethyl) (methyl) amino)benzaldehyde (0.18 g, 1.0 mmol) and *tert*-butyl cyanoacetate (0.169 g, 1.2 mmol) in 10 mL anhydrous methanol, a catalytic amount (2 drops) of piperidine was added. The obtained mixture was stirred and kept at 80 °C for 15 min under the protection of N_2. After cooling to room temperature, the solvent was removed under reduce pressure to give the crude product which was purified with silica gel column chromatography to afford the yellow compound 7. (0.293 g, yield 97%).^{1}H NMR (400 MHz, DMSO-d_6): δ =8.01 (s, 1 H), 7.92 (d, J =9.0 Hz,2 H), 6.85 (d, J =9.0 Hz,2 H), 4.81 (t, J =5.1Hz, 1H), 3.51-3.63(m, 4 H), 3.08 (s, 3 H), 1.51 (s, 9 H). ^{13}H NMR (100 MHz, DMSO-d_6): δ=162.47, 153.45, 153.02, 133.59, 118.07, 117.67, 111.60, 93.34, 81.79, 58.18, 55.97, 53.77, 27.63. MS(ESI):m/z Calcd. For $C_{17}H_{22}N_2O_3$ 302.2; found 303.2, $[M+H]^+$.

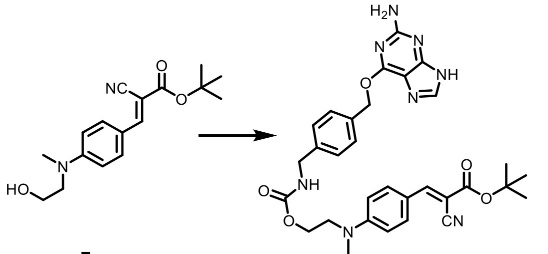

7 **BG-F485**

Compound BG-F485: To a stirred solution of compound 7 (0.302 g, 1.0 mmol) and 4-

dimethylaminopyridine (0.147 g, 1.2 mmol) in 20 mL dry CH_2Cl_2, 4-nitrophenylchloroformate (0.302 g, 1.5 mmol) in 10 mL dry CH_2Cl_2 was added dropwise. The obtained mixture was stirred and kept at room temperature for 2 hr under the protection of N_2. The solvent was removed under reduce pressure to give the intermediate which was used directly without any further purification. After re-dissolved in dry DMF, BG-NH_2 (0.324 g, 1.2 mmol) was added in the presence of 0.2 mL TEA. The obtained mixture was stirred at room temperature for another 1 hr. The solvent was removed under reduce pressure to give the crude production which was purified by gel silica gel column chromatography to afford the yellow BG-F485. (0.556 g, yield 93%).^{1}H NMR (400 MHz, DMSO-d_6): δ = 12.46 (br, 1 H), 10.25 (br, 1 H), 8.01 (s, 1 H), 7.92 (d, J=9.2 Hz, 2 H), 7.81 (s, 1 H), 7.45 (d, J=8.0 Hz, 2 H), 7.27 (d, J=8.0 Hz, 2 H), 6.84 (d, J=9.2 Hz, 2 H), 6.28 (br, 2 H), 5.44 (s, 2 H), 4.27 (d, J=6.0 Hz, 2 H), 3.42-3.47 (m, 4 H), 3.03 (s, 3 H), 1.49 (s, 9 H). ^{13}H NMR (100 MHz, DMSO-d_6): δ=171.48, 162.44, 159.53, 158.28, 153.47, 152.67, 139.42, 135.15, 133.68, 128.49, 127.27, 118.10, 117.64, 111.50, 93.39, 81.81, 86.45, 55.94, 54.38, 50.93, 45.26, 41.91, 41.82, 27.62, 8.32. HR-MS(ESI): m/z Calcd. For $C_{31}H_{34}N_8O_5$ 598.2652; found 621.2620, $[M+Na]^+$.

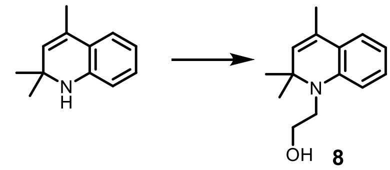

8

Compound 8: To a stirred solution of 2,2,4-trimethyl-1,2-dihydrdro quinolone (0.866 g, 5.0 mmol), 2-bromoethanol (0.750 g, 6.0 mmol) and in 100 mL anhydrous CH_3CN, K_2CO_3 (1.380 g, 10.0 mmol) was added in one portion. The obtained mixture was refluxed for 12 hr under the protection of N_2. After cooling to room temperature, the mixture was filtered and washed with CH_3CN. The solvent was removed under reduce pressure to give the crude product which was purified by silica gel column chromatography to afford the compound 8. (0.857 g, yield 79%). ^{1}H NMR (400 MHz, DMSO-d_6): δ= 7.02 (td, J =7.0Hz, 1 H), 6.97 (dd, J =7.5, 1.5Hz,1 H), 6.48-6.59(m, 2 H), 5.28 (d, J =1.2Hz, 1 H), 4.76 (t, J =5.6Hz, 1 H), 3.46 (dd, J =12.6, 7.0Hz, 2 H), 3.29 (t, J =7.2Hz, 2 H), 1.89 (d, J =1.2Hz, 3 H), 1.24 (s, 6 H). ^{13}H NMR (100 MHz, DMSO-d_6): δ=143.66, 129.30, 128.60, 126.93, 123.38, 122.08, 115.34, 110.10, 58.53, 55.90, 45.59, 28.09, 18.26. MS(ESI):m/z Calcd. For $C_{14}H_{19}NO$ 217.1; found 218.1, $[M+H]^+$.

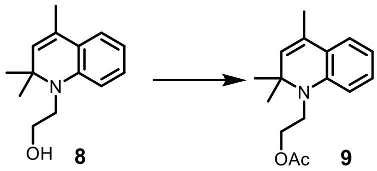

8 9

Compound 9: Acetic anhydride (0.11 g, 1.0 mmol) was added to the solution of compound 8 (0.217 g, 1.0 mmol) and 4-dimethylaminopyidine (0.144 g, 1.2 mmol) in dry CH_2Cl_2 at 0 °C. After addition, the mixture was warmed to room temperature and stirred for a further 1 hr. The reaction mixture was quenched with 1 mL water and extracted with CH_2Cl_2, then dried with Na_2SO_4. The solvent was removed under reduced pressure to give the crude product which was purified by silica gel column to afford the yellow compound 9. (0.254 g, yield 98%). ^{1}H NMR (400 MHz, DMSO-d_6): δ= 6.95-7.09 (m, 2 H), 6.54-6.63 (m, 2 H), 5.31 (d, J =1.2 Hz, 1 H), 4.07 (t, J =6.8 Hz, 2 H), 3.45 (t, J =6.8 Hz, 2 H), 2.03 (s, 3 H), 1.90 (d, J =1.2 Hz, 3 H), 1.24 (s, 6 H). ^{13}H NMR (100 MHz, DMSO-d_6): δ=170.28, 143.18, 129.41, 128.59, 126.89, 123.44, 122.41, 115.93, 110.10, 60.97, 56.01, 41.49, 27.72, 20.55, 18.15. MS(ESI): m/z Calcd. For $C_{16}H_{21}NO_2$ 259.2; found 260.2, $[M+H]^+$.

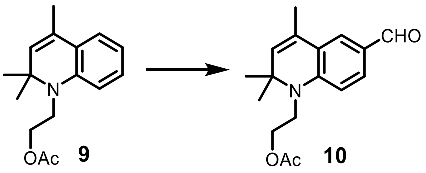

Compound 10: Phosphorous oxychloride (0.22 mL, 2.32 mmol) was added dropwise to a stirred 0 °C solution of compound 9 (0.50 g, 1.93 mmol) in 20 mL dry CH_2Cl_2 and 2 mL DMF under the protection of N_2. The obtained mixture was warmed to room temperature and stirred for a further 5 hr. The mixture was quenched with a saturated solution of sodium carbonate and extracted with CH_2Cl_2. The organic layer was dried over Na_2SO_4, then the solvent was removed under reduced pressure to give the crude produce which was purified by silica gel column chromatography to afford compound 10. (0.477 g, yield 86%). ^{1}H NMR (400 MHz, DMSO-d_6): δ= 9.67 (s, 1 H),7.59 (dd, J =8.4, 2.0 Hz, 1 H), 7.47 (d, J =2.0 Hz, 1 H), 6.77 (d, J =8.8 Hz, 1 H), 5.45 (d, J =1.2 Hz, 1 H), 4.13 (t, J =6.8 Hz, 2 H), 3.62 (t, J =6.8 Hz, 2H), 2.04 (s, 3 H), 1.96 (d, J =1.0 Hz, 3 H), 1.33(s, 6 H). ^{13}H NMR (100 MHz, DMSO-d_6): δ=189.45, 170.30, 148.53, 130.03, 125.92, 124.92, 121.21, 109.94, 60.55, 57.49, 41.83, 28.64, 20.59, 18.03. MS(ESI):m/z Calcd. For $C_{17}H_{21}NO_3$ 287.2; found 288.2, $[M+H]^+$.

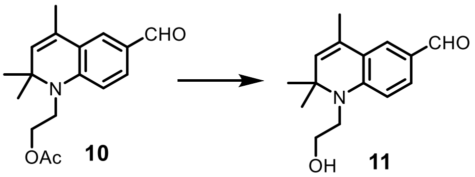

Compound 11: 2 mL saturated solution of sodium carbonate was added to a stirred solution of compound 10 (0.4 g, 1.39 mmol) in 20 mL methanol. The obtained mixture was stirred and kept room temperature for 2 hr under the protection of N_2. The solvents were

removed under reduce pressure, then the residue was re-dissolved in 50 mL CH_2Cl_2 and washed with a saturated solution of NaCl; the organic layer was dried over Na_2SO_4, filtered and removed the solvent under reduce pressure to give the crude product which was purified by silica gel column chromatography to afford the compound 11. (0.341 g, yield 100%). ^{1}H NMR (400 MHz, DMSO-d_6): δ= 9.64 (s, 1 H), 7.57 (dd, J =8.8, 2.0 Hz, 1 H), 7.44 (d, J =2.0 Hz, 1 H), 6.69 (d, J =8.8 Hz, 1 H), 5.42 (d, J =1.2 Hz, 1 H), 4.88 (bt, 1 H), 3.46-3.56 (t, 2H), 3.39-3.47 (t, 2 H), 1.94 (d, J =1.2 Hz, 3 H), 1.33(s, 6H). ^{13}H NMR (100 MHz, DMSO-d_6): δ=189.42, 148.60, 132.04, 129.62, 125.70, 124.17, 123.99, 120.96, 109.59, 57.89, 57.13, 55.74, 45.59, 28.72, 17.84. MS(ESI): m/z Calcd. For $C_{15}H_{19}NO_2$ 245.1; found 246.1, [M+H]$^+$.

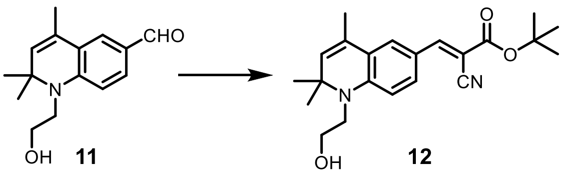

Compound 12: This compound was obtained by following the general procedure for compound 7. (0.215 g, yield 93%).^{1}H NMR (400 MHz, DMSO-d_6): δ= 8.00 (s, 1 H), 7.81 (d, J =2.0 Hz, 1 H), 7.77 (dd, J =9.0, 2.0 Hz, 1 H), 6.72 (d, J =5.2 Hz, 1 H), 5.45 (1H, d, J =1.2 Hz), 3.42-3.54 (m, 4H), 1.92 (d, J =1.2 Hz, 3 H), 1.50 (s, 9H), 1.35 (s, 6H). ^{13}H NMR (100 MHz, DMSO-d_6): δ=162.22, 153.20, 148.29, 134.20, 129.84, 126.20, 125.49, 121.11, 118.11, 117.48, 110.28, 93.35, 81.59, 57.99, 57.59, 45.63, 28.56, 27.41, 17.70. MS(ESI): m/z Calcd. For $C_{22}H_{28}N_2O_3$ 368.2; found 369.2, [M+H]$^+$.

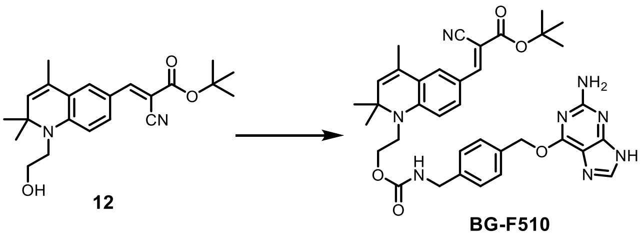

Compound BG-F510: This compound was obtained by following the general procedure for BG-F485. (0.245 g, yield 85%). ^{1}H NMR (400 MHz, DMSO-d_6): δ= 12.42 (br, 1 H), 8.02 (s, 1 H), 7.82 (s, 2 H), 7.77 (d, J=8.8 Hz, 1 H), 7.45 (d, J=8.0 Hz, 2 H), 7.27 (d, J=8.0 Hz, 2 H), 6.83 (d, J=7.2 Hz, 1 H), 6.27 (br, 2 H), 5.45 (s, 2 H), 4.21 (d, J=6.0 Hz, 2 H), 4.09 (t, J=5.6 Hz, 2 H), 3.62 (t, 2 H, J=5.6 Hz), 1.92 (s, 3 H), 1.49 (s, 9 H), 1.32 (s, 6 H). ^{13}H NMR (100 MHz, DMSO-d_6): δ=162.36, 159.59, 156.29, 153.56, 148.36, 139.49, 135.27, 134.36, 130.28, 128.51, 127.10, 126.37, 125.81, 121.75, 118.85, 117.60, 110.69, 94.24, 81.94, 66.48, 62.77, 60.53, 57.96, 55.99, 45.61, 43.60, 42.54, 28.83, 27.65, 17.94. HR-MS(ESI): m/z Calcd. For $C_{36}H_{40}N_8O_5$ 664.3122; found 697.3120, [M+Na]$^+$.

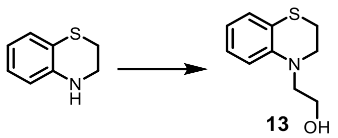

Compound 13: This compound was obtained by following the general procedure for compound 8. (0.703 g, yield 72%). ^{1}H NMR (400 MHz, $CDCl_3$): δ= 7.05 (dd, J = 7.7, 1.4 Hz, 1H), 7.01 – 6.96 (m, 1H), 6.76 (d, J = 8.2 Hz, 1H), 6.66 (t, J = 7.4 Hz, 1H), 4.45 (t, J = 6.1 Hz, 0H), 3.82 (t, J = 5.7 Hz, 2H), 3.67 – 3.63 (m, 2H), 3.46 (t, J = 5.7 Hz, 2H), 3.05 – 3.02 (m, 2H). ^{13}C NMR (100 MHz, $CDCl_3$) δ=143.48, 127.99, 125.87, 118.67, 118.18, 114.04, 59.68, 55.18, 50.58, 25.49. MS(ESI):m/z Calcd. For $C_{10}H_{13}NOS$ 195.1; found 196.1, $[M+H]^+$.

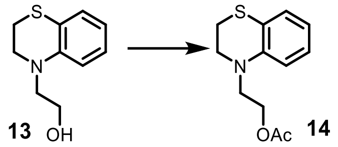

Compound 14: This compound was obtained by following the general procedure for compound 9. (0.539 g, yield 98%). ^{1}H NMR (400 MHz, $CDCl_3$): δ= 7.05 (dd, J = 7.7, 1.4 Hz, 1H), 7.01 – 6.96 (m, 1H), 6.76 (d, J = 8.2 Hz, 1H), 6.66 (t, J = 7.4 Hz, 1H), 3.82 (t, J = 5.7 Hz, 2H), 3.67 – 3.63 (m, 2H), 3.46 (t, J = 5.7 Hz, 2H), 3.05 – 3.02 (m, 2H), 2.03 (s, 3 H). ^{13}C NMR (100 MHz, $CDCl_3$): δ=143.48, 127.99, 125.87, 118.67, 118.18, 114.04, 109.32, 59.68, 55.18, 50.58, 25.49. MS(ESI): m/z Calcd. For $C_{12}H_{15}NO_2S$ 237.1; found 238.1, $[M+H]^+$.

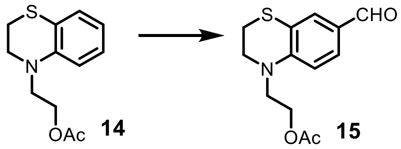

Compound 15: This compound was obtained by following the general procedure for compound 10. (0.521 g, yield 98%). ^{1}H NMR (400 MHz, $CDCl_3$): δ=9.89 (s,1 H), 7.06 (dd, J = 7.7, 1.4 Hz, 1H), 7.01 – 6.90 (m, 1H), 6.78 (d, J = 8.2 Hz, 1H), 6.69 (t, J = 7.4 Hz, 1H), 3.82 (t, J = 5.7 Hz, 2H), 3.67 – 3.60 (m, 2H), 3.45 (t, J = 5.7 Hz, 2H), 3.05 – 3.02 (m, 2H), 2.02 (s, 3 H). ^{13}C NMR (100 MHz, $CDCl_3$): δ= 181.2, 143.48, 127.99, 125.87, 118.67, 118.18, 114.04, 109.32, 59.68, 55.18, 50.58, 25.49. MS(ESI): m/z Calcd. For $C_{13}H_{15}NO_2S$ 265.1; found266.1, $[M+H]^+$.

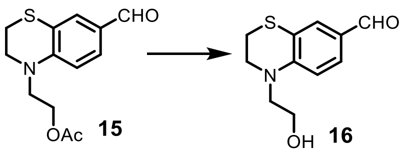

Compound 16: This compound was obtained by following the general procedure for compound 11. (0.489 g, yield 88%). ^{1}H NMR (400 MHz, $CDCl_3$): δ=9.54 (s, 1H), 7.45 (d, J = 2.0 Hz, 1H), 7.45 (d, J = 2.0 Hz, 1H), 7.37 (dd, J = 8.7, 2.0 Hz, 1H), 7.37 (dd, J = 8.7, 2.0 Hz, 1H), 6.70

(d, J = 8.7 Hz, 1H), 3.89 – 3.81 (m, 4H), 3.57 (t, J = 5.7 Hz, 2H), 3.02 – 2.97 (m, 2H). ^{13}C NMR (100 MHz, $CDCl_3$): δ=190.07, 148.05, 125.63, 117.02, 111.29, 60.47, 59.42, 54.69, 51.90, 24.42. MS(ESI):m/z Calcd. For $C_{11}H_{13}NO_2S$ 223.01; found 224.1, $[M+H]^+$.

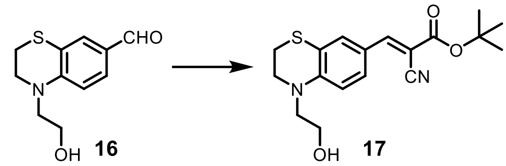

Compound 17: This compound was obtained by following the general procedure for compound 7. (0.321 g, yield 94%).^{1}H NMR (400 MHz, $CDCl_3$): δ=7.82 (s, 1H), 7.69 (dd, J = 9.0, 2.1 Hz, 2H), 7.54 (d, J = 2.2 Hz, 1H), 6.73 – 6.70 (m, 1H), 3.90 – 3.84 (m, 4H), 3.60 (q, J = 5.2 Hz, 4H), 3.03 – 2.98 (m, 4H). 13C NMR (100 MHz, $CDCl_3$): δ= 162.85, 152.88, 147.08, 132.35, 132.22, 130.23, 120.33, 120.15, 117.49, 117.35, 117.22, 111.73, 96.93, 82.80, 59.59, 54.54, 52.17, 52.09, 28.06, 24.41, 24.32. MS(ESI): m/z Calcd. For $C_{18}H_{22}N_2O_3S$ 346.1; found 347.1, $[M+H]^+$.

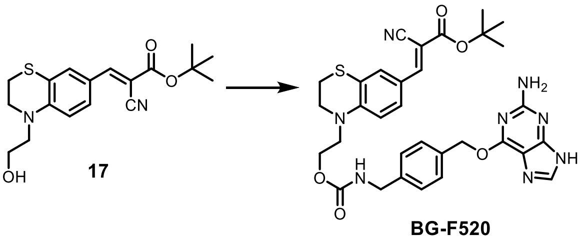

Compound BG-F520: This compound was obtained by following the general procedure for BG-F485. (0.212 g, yield 89%). ^{1}H NMR (400 MHz, DMSO-d_6): δ= 12.43 (s, 1H), 7.99 (s, 1H), 7.81 (s, 1H), 7.77 (d, J = 6.0 Hz, 1H), 7.58 (d, J = 1.8 Hz, 1H), 7.50 (dd, J = 8.7, 1.7 Hz, 1H), 7.43 (d, J = 7.9 Hz, 2H), 7.22 (d, J = 7.9 Hz, 2H), 6.91 (d, J = 8.8 Hz, 1H), 6.30 (s, 1H), 5.76 (s, 1H), 5.44 (s, 1H), 4.23 – 4.11 (m, 6H), 3.68 (t, J = 5.0 Hz, 2H), 3.56 (s, 2H), 1.49 (s, 5H). ^{13}H NMR (100 MHz, DMSO-d_6): δ=162.74, 160.10, 156.69, 153.94, 142.94, 141.11, 138.24, 129.01, 127.50, 119.62, 117.92, 117.09, 111.30, 95.03, 82.51, 63.72, 55.39, 44.03, 28.12. HR-MS(ESI): m/z Calcd. For $C_{32}H_{34}N_8O_5S$, 642.2373; found 665.2371, $[M+Na]^+$.

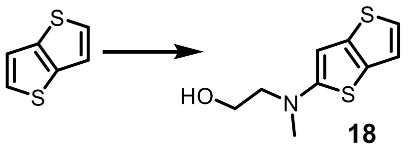

Compound 18: N-bromosuccinimide (0.64 g, 3.57 mmol) was added to a solution of thieno[3,2-b]thiophene (0.50 g, 3.57 mmol) in 20 mL DMF. The obtained mixture was stirred and kept at room temperature for 5 hr under the protection of N_2. Then, the mixture was pour into 100 mL water and extracted with CH_2Cl_2. The organic layer was dried with Na_2SO_4, filtered,

and the solvent removed under reduce pressure to obtain intermediate which was used for the next step without further purification. The intermediate was dissolved in 10 mL 2-methylaminoethanol, then, CuI (76 mg, 0.4 mmol), K_3PO_4 (0.829 g, 6.0 mmol), (L)-proline (92 mg, 0.80 mmol) were added and the obtained mixture was stirred overnight at 90 ^oC. After cooling to room temperature, 50 mL water was added to the mixture. The mixture was extracted with CH_2Cl_2, dried over Na_2SO_4. The solution was filtered and the solvent was removed under reduce pressure to give the crude product which was purified by silica gel column chromatography to afford compound 18. (0.556 g, yield 73%). ^{1}H NMR (400 MHz, DMSO-d_6): δ= 7.15 (q, J =5.2Hz, 2H), 6.16 (s, 1 H), 4.77 (bt, t, J =5.3Hz,1 H), 3.61 (2H, q, J =5.7Hz), 3.31 (2H, t, J =6.0Hz), 2.95 (3H, s). ^{13}H NMR (100 MHz, DMSO-d_6): δ=159.71, 138.91, 124.52, 120.09, 119.72, 93.28, 57.91, 57.11, 40.25. MS(ESI): m/z Calcd. For $C_9H_{11}NOS_2$ 213.0; found 214.0, $[M+H]^+$.

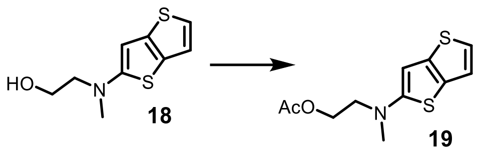

Compound 19: This compound was obtained by following the general procedure for compound 9. (0.509 g, yield 95%). ^{1}H NMR (400 MHz, DMSO-d_6): δ= 7.18 (s, 2 H), 6.24 (s, 1 H), 4.23 (t, J =5.5 Hz, 2 H), 3.50 (t, J =5.5 Hz, 2 H), 2.94 (s, 3 H), 1.97 (s, 3 H). ^{13}H NMR (100 MHz, DMSO-d_6): δ=170.03, 158.98, 138.52, 124.79, 120.39, 119.52, 94.23, 60.49, 53.13, 39.76, 20.40. MS(ESI):m/z Calcd. For $C_{11}H_{12}NO_2S_2$ 255.0; found 256.0, $[M+H]^+$.

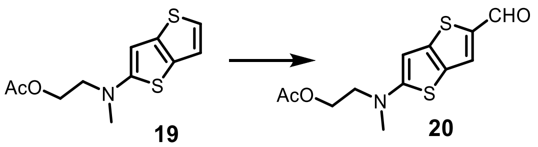

Compound 20: This compound was obtained by following the general procedure for compound 10. (0.433 g, yield 79%). ^{1}H NMR (400 MHz, DMSO-d_6): δ= 9.89 (s, 1 H), 7.99 (s, 1 H), 6.24 (s, 1H), 4.23 (t, J =5.6 Hz, 2 H), 3.50 (t, J =5.6 Hz, 2 H), 2.94 (s, 3 H), 1.97 (s, 3H). ^{13}H NMR (400 MHz, DMSO-d_6): δ=180.02, 170.03, 158.98, 138.52, 124.79, 120.39, 119.52, 94.23, 60.49, 53.13, 39.76, 20.40. MS(ESI):m/z Calcd. For $C_{12}H_{13}NO_3S_2$ 283.0; found 284.0, $[M+H]^+$.

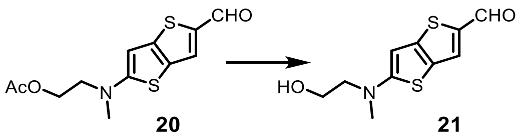

Compound 21: This compound was obtained by following the general procedure for compound 11. (0.374 g, yield 98%). ^{1}H NMR (400 MHz, DMSO-d_6): δ= 9.66 (s, 1 H), 8.05 (s, 1

H), 6.30 (s, 1 H), 4.88 (bt, 1 H), 3.64 (t, J =5.6 Hz, 2 H), 3.44 (t, J =5.6 Hz, 2 H), 3.07 (s, 3 H). ^{13}H NMR (100 MHz, DMSO-d_6): δ=181.01, 165.59, 149.11, 135.79, 131.60, 124.55, 92.80, 57.67, 56.67, 40.12. MS(ESI): m/z Calcd. For $C_{10}H_{11}NO_2S_2$ 241.0; found 242.0, $[M+H]^+$.

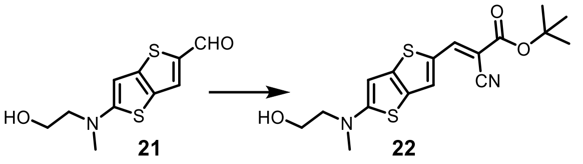

Compound 22: This compound was obtained by following the general procedure for compound 7. (0.351 g, yield 93%). ^{1}H NMR (400 MHz, DMSO-d_6): δ= 8.22 (s, 1 H), 8.02 (s, 1 H), 6.43 (s, 1 H), 4.91 (t, J =5.6 Hz, 1 H), 3.65(t, J =5.6 Hz, 2 H), 3.48 (t, J =5.2 Hz, 2 H), 3.12 (s, 3H), 1.49 (s, 9 H). ^{13}H NMR (100 MHz, DMSO-d_6): δ =167.40, 162.82, 152.58, 145.78, 128.65, 125.67, 117.75, 93.91, 88.83, 81.38, 57.88, 57.08, 40.52, 27.70, 18.48. MS(ESI): m/z Calcd. For $C_{17}H_{20}N_2O_3S_2$ 364.1; found 365.1, $[M+H]^+$.

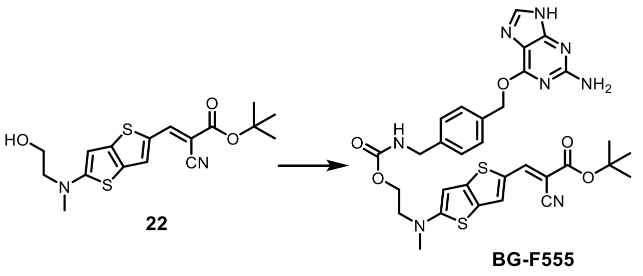

Compound BG-F555: This compound was obtained by following the general procedure for BG-F485. (0.335 g, yield 81%). ^{1}H NMR (400 MHz, DMSO-d_6): δ=12.46 (br, 1 H), 8.26 (s, 1 H), 8.04 (s, 1 H), 7.82 (s, 1 H), 7.77 (t, 1 H, J=6.0 Hz), 7.40 (d, 2 H, J=8.0 Hz), 7.21 (d, 2 H, J=8.0 Hz), 6.46 (s, 1 H), 6.28 (br, 2 H), 5.43 (s, 2 H), 4.24 (t, 2 H, J=4.8 Hz), 4.16 (d, 2 H, J=6.0 Hz), 3.65 (t, 2 H, J=4.8 Hz), 3.09 (s, 3 H), 1.51 (s, 9 H). ^{13}H NMR (100 MHz, DMSO-d_6): δ =167.01, 162.74, 159.56, 156.06, 152.28, 145.98, 139.43, 135.16, 133.73, 133.67, 129.02, 128.41, 126.94, 125.90, 117.69, 94.23, 89.41, 81.48, 66.45, 60.68, 55.96, 53.83, 45.49, 43.51, 40.23, 27.69, 18.49, 8.58. HR-MS(ESI):m/z Calcd. For $C_{31}H_{32}N_8O_5S_2$ 660.1937; found 683.1940, $[M+Na]^+$.

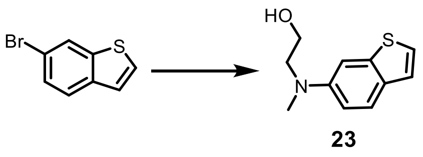

Compound 23: 6-bromobenzothiophene (0.426 g, 2.0 mmol), CuI (76 mg, 0.4 mmol), K_3PO_4 (0.829 g, 6.0 mmol), (L)-proline (92 mg, 0.80 mmol), and 10 mL 2-methylaminoethanol were stirred at 90 °C overnight under the protection of N_2. After cooling to room temperature, 50 mL water was added to the mixture. The organic compounds were extracted with CH_2Cl_2,

and dried over Na_2SO_4. The solution was filtered and the solvent removed under reduce pressure to give the crude product which was purified by silica gel column chromatography to afford compound 23. (0.323 g, yield 78%). ^{1}H NMR (400 MHz, DMSO-d_6): δ= 7.63 (d, J =8.8 Hz, 1 H), 7.28 (d, J =5.6 Hz, 1 H), 7.21 (d, J =5.4 Hz, 1 H), 7.17 (d, J =1.6 Hz, 1 H), 6.89 (dd, J =8.8, 2.4 Hz, 1 H), 4.69 (bt, 1H), 3.57 (t, J =6.0Hz, 2H), 3.44(t, J =6.4 Hz, 2H), 2.97 (s, 3H). ^{13}H NMR (100 MHz, DMSO-d_6): δ=147.05, 141.41, 129.71, 123.58, 123.34, 121.37, 111.65, 103.40, 58.10, 54.72,38.97. MS(ESI): m/z Calcd. For $C_{11}H_{13}NOS$ 207.1; found 207.1, $[M+H]^+$.

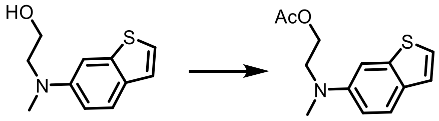

23 24

Compound 24: This compound was obtained by following the general procedure for compound 9. (0.355 g, yield 99%). ^{1}H NMR (400 MHz, DMSO-d_6): δ = 7.65 (d, J =8.8 Hz, 1 H), 7.31 (d, J =5.6 Hz, 1 H), 7.22 (d, J =5.2 Hz, 2 H), 6.93 (dd, J =8.8, 2.4 Hz, 1H), 4.19 (t, J =5.8 Hz, 2 H), 3.64 (t, J =5.6 Hz, 2H), 2.97 (s, 3H), 1.94 (s, 3H). ^{13}H NMR (100 MHz, DMSO-d_6): δ=170.33, 146.61, 141.40, 130.12, 123.66, 123.33, 121.79, 111.76, 103.82, 61.10, 50.68, 38.69, 20.61. MS(ESI): m/z Calcd. For $C_{13}H_{15}NO_2S$ 249.1; found 250.1, $[M+H]^+$.

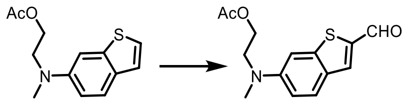

24 25

Compound 25: This compound was obtained by following the general procedure for compound 10. (0.322 g, yield 75%). ^{1}H NMR (400 MHz, DMSO-d_6): δ= 9.93 (1H, s), 8.15 (1H, s), 7.83 (1H, d, J =9.1Hz), 7.23 (1H, d, J =2.2Hz), 7.03 (1H, dd, J =9.1, 2.4Hz), 4.21 (2H, t, J =5.7Hz), 3.72 (2H, t, J =5.7Hz), 3.03 (3H, s), 1.93 (3H, s). ^{13}H NMR (100 MHz, DMSO-d_6): δ=184.48, 170.30, 149.52, 145.11, 137.43, 136.65, 128.71, 127.17, 112.78, 102.94, 61.08, 50.22, 38.69, 20.57. MS(ESI): m/z Calcd. For $C_{14}H_{15}NO_3S$ 277.1; found 278.1, $[M+H]^+$.

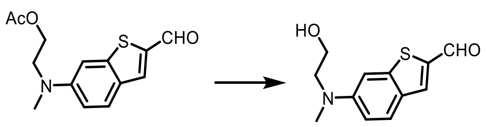

25 26

Compound 26: This compound was obtained by following the general procedure for compound 11. (0.235 g, yield 95%). ^{1}H NMR (400 MHz, DMSO-d_6): δ =9.91 (s, 1 H), 8.14(s, 1 H), 7.81 (d, J=5.2 Hz, 1 H), 7.17 (d, J=2.0 Hz, 1 H), 7.01 (dd, J=2.0, 8.8 Hz, 1 H), 4.76 (t, J=5.6 Hz, 1 H), 3.58 (t, J=4.2 Hz, 2 H), 3.52 (t, J=4.2 Hz, 2 H), 3.04 (s, 3 H). ^{13}H NMR (100 MHz, DMSO-d_6): δ=183.89, 149.47, 144.75, 136.52, 136.31, 127.85, 126.68, 112.34, 102.08, 57.73, 53.71, 38.99.

MS(ESI):m/z Calcd. For $C_{12}H_{13}NO_2S$ 235.1; found 236.1, $[M+H]^+$.

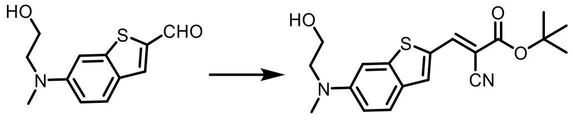

26 27

Compound 27: This compound was obtained by following the general procedure for compound 7. (0.255 g, yield 89%). ^{1}H NMR (400 MHz, DMSO-*d6*): δ = 8.41 (s, 1 H), 8.10 (s, 1 H), 7.76 (d, J =9.2 Hz, 1 H), 7.24 (d, J =2.0 Hz, 1H),7.01 (dd, J =9.2, 2.4 Hz, 1 H), 4.75 (bt, 1H), 3.61 (t, J =5.2 Hz, 2 H), 3.55 (t, J =5.2 Hz, 2 H), 3.07 (s, 3 H), 1.52 (s, 9 H). ^{13}H NMR (100 MHz, DMSO-*d6*): δ =161.79, 150.12, 147.28, 146.52, 139.12, 129.50, 127.70, 126.65, 116.57, 113.20, 102.19, 95.97, 82.35, 58.25, 54.20, 27.63. MS(ESI): m/z Calcd. For $C_{19}H_{22}N_2O_3S$ 358.1; found 358.1, $[M+H]^+$.

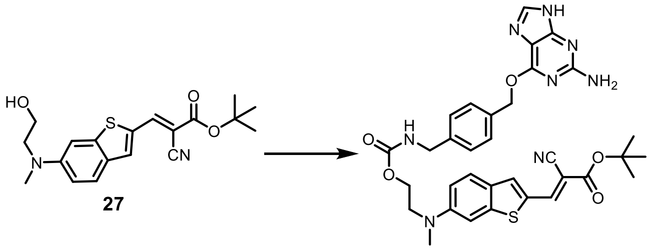

BG-F570

Compound BG-F570: This compound was obtained by following the general procedure for BG-F485. (0.315 g, yield 86%). ^{1}H NMR (400 MHz, DMSO-*d6*): δ =12.50 (br, 1 H), 8.43 (s, 1 H), 8.12 (s, 1 H), 7.85 (s, 1 H), 7.71 (m, 1 H), 7.32 (d, 2 H, J=8.0 Hz), 7.30 (s, 1 H), 7.22 (d, 2 H, J=8.0 Hz), 7.02 (dd, J=2.0, 9.2 Hz, 1 H), 6.30 (br, 2 H), 5.44 (s, 2 H), 4.17 (m, 4 H), 3.71 (t, 2 H, J=5.6 Hz), 3.06 (s, 3 H), 1.51 (s, 9 H). ^{13}H NMR (100 MHz, DMSO-*d6*): δ=161.73, 159.56, 156.29, 149.81, 147.38, 146.43, 139.52, 139.12, 135.17, 129.83, 128.45, 128.01, 127.00, 126.76, 116.53, 113.12, 102.47, 96.32, 82.42, 66.53, 61.12, 50.80, 45.51, 43.52, 30.93, 27.62, 22.04, 13.94, 8.50. HR-MS(ESI): m/z Calcd. For $C_{33}H_{34}N_8O_5S$ 654.2373; found 677.2370, $[M+Na]^+$.

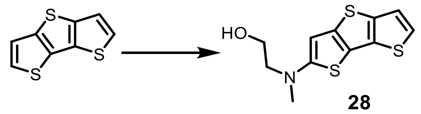

28

Compound 28: This compound was obtained by following the general procedure for compound 18. (0.530 g, yield 81%). ^{1}H NMR (400 MHz, CDCl$_3$): δ= 7.11 (q, J =5.2 Hz, 2 H), 6.16 (s, 1 H), 4.77 (bt, t, J =5.2 Hz, 1 H), 3.61 (q, J =5.6 Hz, 2 H), 3.31 (t, J =6.0 Hz, 2 H), 2.95 (s, 3H). ^{13}H NMR (100 MHz, CDCl$_3$): δ= 156.71, 155.31, 138.91, 124.52, 120.09, 119.72, 110.51, 93.28, 57.91, 57.11, 40.25. MS(ESI):m/z Calcd. For $C_{11}H_{11}NO_2S_3$ 269.0; found 270.0, $[M+H]^+$.

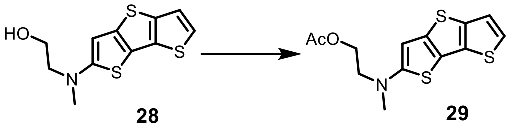

Compound 29: This compound was obtained by following the general procedure for compound 9. (0.560 g, yield 98%). ^{1}H NMR (400 MHz, DMSO-d_6): δ= 7.15 (s, 2 H), 6.24 (s, 1 H), 4.23 (t, J =5.4 Hz,2 H), 3.50 (t, J =5.4 Hz, 2 H), 2.94 (s, 3 H), 1.97 (3H, s). ^{13}H NMR (100 MHz, DMSO-d_6): δ=171.21, 156.51, 155.11, 139.21, 124.22, 120.79, 119.12,110.01, 93.28, 57.91, 57.11, 40.25, 20.41. MS(ESI): m/z Calcd. For $C_{13}H_{13}NOS_3$ 311.0; found 312.0, $[M+H]^+$.

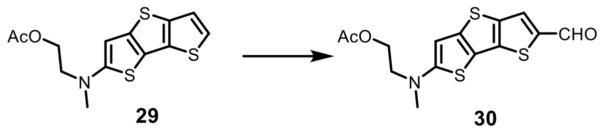

Compound 30: This compound was obtained by following the general procedure for compound 10. (0.455 g, yield 73%). ^{1}H NMR (400 MHz, DMSO-d_6): δ= 9.97 (s, 1 H), 8.41 (s, 1 H), 7.48 (s, 1 H), 4.23 (t, J =5.4 Hz, 2 H), 3.50 (t, J =5.4 Hz, 2 H), 2.94 (s, 3 H), 1.97 (s, 3H). ^{13}H NMR (100 MHz, DMSO-d_6): δ=183.21, 171.11, 156.41, 154.91, 139.71, 124.72, 120.71, 119.32,110.01, 93.48, 57.91, 57.11, 40.25, 20.41. MS(ESI): m/z Calcd. For $C_{14}H_{13}NO_3S_3$ 339.0; found 340.0, $[M+H]^+$.

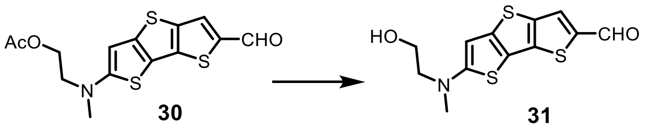

Compound 31: This compound was obtained by following the general procedure for compound 11. (0.357 g, yield 97%). ^{1}H NMR (400 MHz, $CDCl_3$): δ= 9.91 (s, 1 H), 8.38 (s, 1H), 7.44 (s, 1 H), 4.90 (t, J=5.6 Hz, 1 H), 3.65(q, J=5.6 Hz, 3 H), 4.90 (t, J=5.6 Hz, 3 H), 1.50 (s, 9 H). ^{13}H NMR (100 MHz, $CDCl_3$): δ=183.11, 156.41, 154.91, 139.71, 124.72, 120.71, 119.32, 110.01, 93.48, 57.91, 57.11, 40.25. MS(ESI): m/z Calcd. For $C_{12}H_{11}NO_2S_3$ 297.0; found 320.0, $[M+Na]^+$.

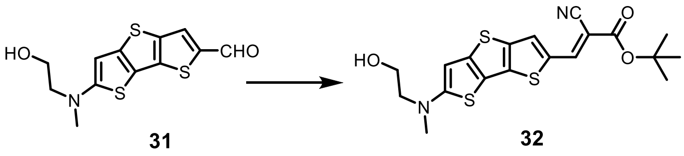

Compound 32: This compound was obtained by following the general procedure for compound 7. (0.298 g, yield 89%). ^{1}H NMR (400 MHz, $CDCl_3$): δ= 8.36 (s, 1 H), 8.21 (s, 1H), 6.40 (s, 1 H), 4.90 (t, J=5.6 Hz, 1 H), 3.65(q, J=5.6 Hz, 3 H), 4.90 (t, J=5.6 Hz, 3 H), 1.50(s, 9 H). ^{13}H NMR (100 MHz, $CDCl_3$): δ =165.31, 162.69, 151.18, 147.07, 141.74, 136.16, 134.49, 131.92, 117.68, 114.99, 95.05, 92.76, 82.55, 58.42, 57.49, 28.18, 20.41. MS(ESI): m/z Calcd. For $C_{19}H_{20}N_2O_3S_3$ 420.0; found 421.0, $[M+H]^+$.

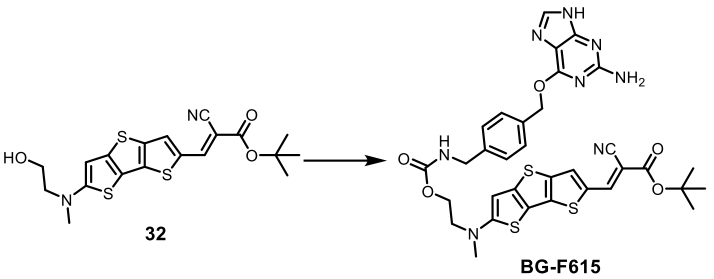

Compound BG-F615: This compound was obtained by following the general procedure for BG-F485. (0.265 g, yield 86%). ^{1}H NMR (400 MHz, DMSO-d_6): δ= 8.53(t, J=5.8 Hz, 1 H), 8.36 (s, 1 H), 8.21 (s, 1H), 7.79 (s, 1 H), 7.44 (d, J=7.8 Hz, 2 H), 7.30 (d, J=7.8 Hz, 2 H), 6.40 (s, 1 H), 6.27 (s, 2 H), 5.44 (s, 2 H), 4.90 (t, J=5.6 Hz, 1 H), 4.37 (d, J=5.8 Hz, 2 H), 3.65(q, J=5.6 Hz, 3 H), 4.90 (t, J=5.6 Hz, 3 H), 1.50(s, 9 H). ^{13}H NMR (100 MHz, DMSO-d_6): δ =165.31, 162.69, 159.62, 155.20, 151.18, 147.07, 141.74, 139.28, 137.76, 136.16, 135.30, 134.49, 131.92, 128.44, 127.40, 117.68, 114.99, 95.05, 92.76, 82.55, 58.42, 57.49, 28.18, 20.41. HR-MS(ESI): m/z Calcd. For $C_{33}H_{32}N_8O_5S_3$ 716.1568; found 739.1570, $[M+Na]^+$.

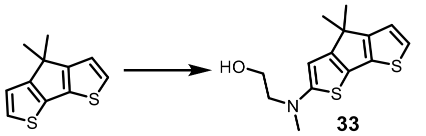

Compound 33: This compound was obtained by following the general procedure for compound 18. (0.525 g, yield 76%). ^{1}H NMR (400 MHz, DMSO-d_6): δ= 7.15 (s, 1 H), 6.76(q, J =5.2 Hz, 2 H), 4.77 (t, J =5.2 Hz, 1 H), 3.61 (q, J =5.6 Hz, 2 H), 3.31 (t, J =6.0 Hz, 2 H), 2.95 (s, 3H) ,1.49(s, 6 H). ^{13}H NMR (100 MHz, DMSO-d_6): δ=158.61, 155.22, 136.81, 125.04, 124.53,111.09, 110.49, 58.92, 58.31, 55.23, 40.29, 30.11, 29.62, 10.21. MS(ESI): m/z Calcd. For $C_{14}H_{17}NOS_2$ 279.1; found 280.1, $[M+H]^+$.

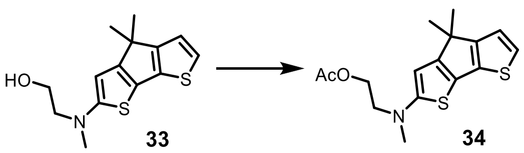

Compound 34: This compound was obtained by following the general procedure for compound 9. (0.415 g, yield 98%). ^{1}H NMR (400 MHz, DMSO-d_6): δ= 7.15 (s, 1 H), 6.76 (q, J =5.2 Hz, 2 H), 4.77 (t, J =5.2 Hz, 1 H), 3.61 (q, J =5.6 Hz, 2 H), 3.31 (t, J =6.0 Hz, 2 H), 2.95 (s, 3 H), 1.97 (s, 3 H), 1.49 (s, 6 H). ^{13}H NMR (100 MHz, DMSO-d_6): δ= 170.21, 158.61, 155.22, 136.81, 125.04, 124.53,111.09, 110.49, 58.92, 58.31, 55.23, 40.29, 30.11, 29.62, 20.41,10.21. MS(ESI): m/z Calcd. For $C_{16}H_{19}NO_2S_2$ 321.1; found 322.1, $[M+H]^+$.

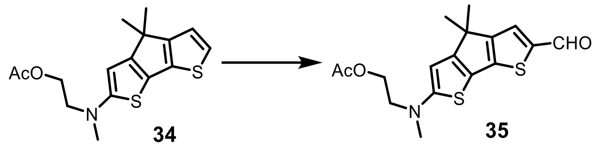

Compound 35: This compound was obtained by following the general procedure for compound 10. (0.481 g, yield 75%). ^{1}H NMR (400 MHz, DMSO-d_6): δ= 9.87 (s, 1 H), 7.15 (s, 1 H), 6.76 (q, J =5.2 Hz, 2 H), 4.77 (t, J =5.2 Hz, 1 H), 3.61 (q, J =5.6 Hz, 2 H), 3.31 (t, J =6.0 Hz, 2 H), 2.95 (s, 3 H), 19.7(s, 3 H), 1.49 (s, 6 H). ^{13}H NMR (100 MHz, DMSO-d_6): δ= 183.06, 170.21, 158.61, 155.22, 136.81, 125.04, 124.53, 111.09, 110.49, 58.92, 58.31, 55.23, 40.29, 30.11, 29.62, 20.41, 10.21. MS(ESI): m/z Calcd. For $C_{17}H_{19}NO_3S_2$ 349.1; found 372.1, [M+Na]$^+$.

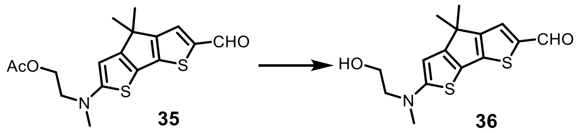

Compound 36: This compound was obtained by following the general procedure for compound 11. (0.320 g, yield 95%). ^{1}H NMR (400 MHz, DMSO-d_6): δ= 9.87 (s, 1 H), 7.15 (s, 1 H), 6.76 (q, J =5.2 Hz, 2 H), 4.77 (t, J =5.2 Hz, 1 H), 3.61 (q, J =5.6 Hz, 2 H), 3.31 (t, J =6.0 Hz, 2 H), 2.95 (s, 3 H) ,1.97(s, 3 H), 1.49(s, 6 H). ^{1}H NMR (400 MHz, DMSO-d_6): δ= 182.58, 163.22, 159.58, 145.64, 143.75, 134.73, 129.41, 124.34, 116.46, 77.22, 58.92, 58.31, 55.23, 45.97, 40.29, 24.81. MS(ESI): m/z Calcd. For $C_{15}H_{17}NO_2S_2$ 307.1; found 308.1, [M+H]$^+$.

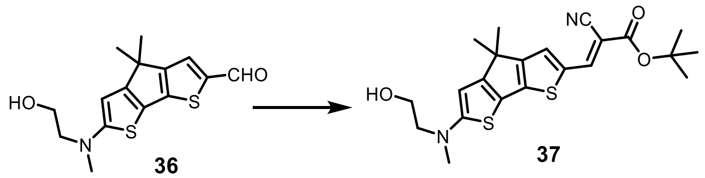

Compound 37: This compound was obtained by following the general procedure for compound 7. (0.256 g, yield 91%). ^{1}H NMR (400 MHz, DMSO-d_6): δ= 8.12 (s, 1 H), 7.75 (s, 1 H), 6.22 (s, 1 H), 4.89 (t, J =5.6 Hz, 1 H), 3.65 (t, J =5.6 Hz, 2 H), 3.48 (t, J =5.6 Hz, 2 H), 3.09 (s, 3 H), 1.49 (s, 9H), 1.39 (s, 6 H). ^{13}H NMR (100 MHz, DMSO-d_6): δ=170.62, 167.36, 163.21, 153.00, 152.14, 145.20, 130.80, 118.47, 115.36, 96.26, 86.40, 80.97, 57.79, 57.06, 44.48, 40.63, 27.76, 24.81. MS(ESI): m/z Calcd. For $C_{22}H_{26}N_2O_3S_2$ 430.1; found 431.1, [M+H]$^+$.

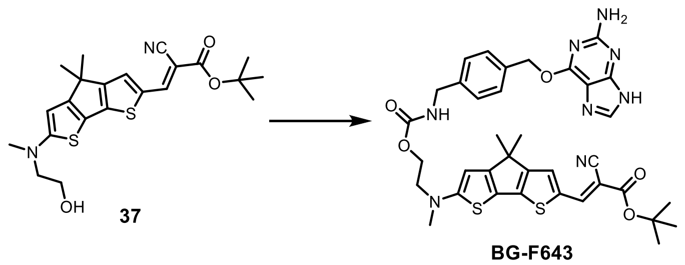

Compound BG-F643: This compound was obtained by following the general procedure

for BG-F485. (0.225 g, yield 85%). ^{1}H NMR (400 MHz, DMSO-d_6): δ=12.03 (s,1 H), 8.55(t, J=5.8 Hz, 1 H), 8.12 (s, 1 H), 7.79 (s, 1 H), 7.75 (s, 1 H), 7.44 (d, J=7.9 Hz, 2 H), 7.30 (d, J=7.9 Hz, 2 H), 6.27(s, 2 H), 6.22 (s, 1 H), 5.44(s, 2 H), 4.89 (t, J =5.6 Hz, 1 H), 4.37 (d, J=5.8 Hz, 2 H), 3.65(t, J =5.6 Hz, 2 H), 3.48 (t, J =5.6 Hz, 2 H), 3.09 (s, 3 H), 1.49 (s, 9H), 1.39 (s, 6 H). ^{13}H NMR (100 MHz, DMSO-d_6): δ=170.62, 167.36, 163.21, 159.62, 155.18, 153.00, 152.14, 145.20, 139.28, 137.76, 135.30, 130.80, 128.44, 127.41, 118.47, 115.36, 113.51, 96.26, 86.40, 80.97, 57.79, 57.06, 44.48, 40.63, 27.76, 24.81. HR-MS(ESI): m/z Calcd. For $C_{36}H_{38}N_8O_5S_2$ 726.2407; found 759.2405, $[M+Na]^+$.

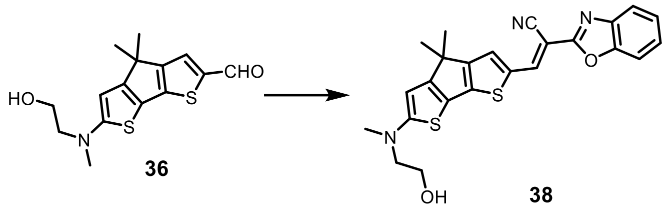

Compound 38: To a stirred solution of compound 36 (0.20 g, 0.65 mmol) and 2-(1,3-benzoxazol-2-yl)acetonitrile (0.124 g, 0.78 mmol) in 10 mL anhydrous methanol, a catalytic amount (2 drops) of piperidine was added. The obtained mixture was stirred and kept at 80 °C for 15 min. After cooling to room temperature, the solvent was removed under reduce pressure to give the crude product which was purified by silica gel column chromatography to afford the black compound 38. (0.293 g, yield 97%). ^{1}H NMR (400 MHz, DMSO-d_6): δ= 8.37 (s, 1 H), 7.81 (s, 1 H), 7.64-7.71 (m, 2 H), 7.30-7.38 (m, 2 H), 6.24 (s, 1 H), 4.90 (t, J =5.2 Hz, 1 H), 3.66 (t, J =6.0 Hz, 2 H), 3.47 (t, J =6.0 Hz, 2 H), 3.10 (s, 3H), 1.42 (s, 6 H). ^{13}H NMR (100 MHz, DMSO-d_6): δ=170.58, 167.38, 161.21, 152.36, 149.90, 141.86, 140.75, 131.87, 124.74, 124.41, 118.65, 117.69, 115.56, 110.17, 96.37, 57.81, 57.09, 44.52, 40.66, 24.87. MS(ESI): m/z Calcd. For $C_{24}H_{22}N_3O_2S_2$ 447.1; found 448.1, $[M+H]^+$.

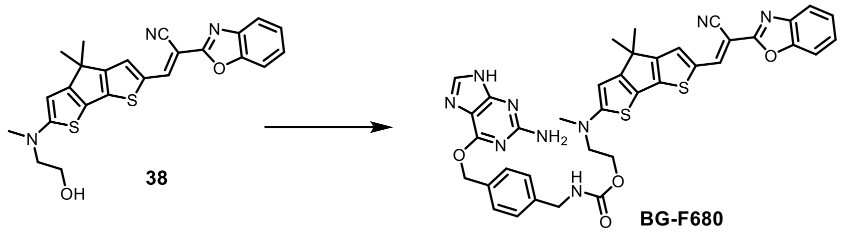

Compound BG-F680: This compound was obtained by following the general procedure for BG-F485. (0.182 g, yield 80%). ^{1}H NMR (400 MHz, DMSO-d_6): δ= 8.55(t, J=5.8 Hz, 1 H), 8.37 (s, 1 H), 7.79(s, 1 H), 7.81 (s, 1 H), 7.64-7.71 (m, 2 H), 7.44 (d, J=7.9 Hz, 2 H), 7.30-7.38 (m, 4 H), 6.27(s, 2 H), 6.24 (s, 1 H), 5.44 (s, 2 H), 4.90 (t, J =5.2 Hz, 1 H), 4,37 (d, J=5.8 Hz, 2 H), 3.66 (t, J =6.0 Hz, 2 H), 3.47 (t, J =6.0 Hz, 2 H), 3.10 (s, 3H), 1.42 (s, 6 H). ^{13}H NMR (100 MHz, DMSO-d_6): δ=170.58, 167.38, 161.21, 159.62, 155.31, 152.36, 149.90, 141.86, 140.75, 139.38, 137.55,

135.30, 131.87, 128.44, 127.21, 124.74, 124.41, 118.65, 117.69, 115.56, 110.17, 96.37, 665.50, 57.81, 57.09, 44.52, 42.85, 40.66, 24.87. HR-MS(ESI): m/z Calcd. For $C_{38}H_{33}N_9O_4S$ 743.2097; found 766.2096, $[M+Na]^+$.

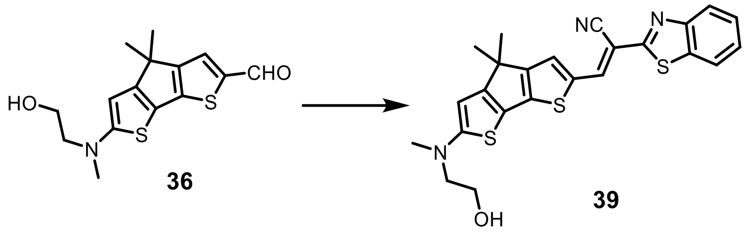

Compound 39: To a stirred solution of compound (0.25 g, 0.81 mmol) and 2-(1,3-benzothiazole-2-yl)acetonitrile (0.17 g, 0.98 mmol) in 10 mL anhydrous methanol, a catalytic amount (2 drops) of piperidine was added. The obtained mixture was stirred at 80 °C for 15 min under the protection of N_2. After cooling to room temperature, the solvent was removed under reduce pressure to give the crude production which was purified by silica gel column chromatography to afford the black compound 39. (0.336 g, yield 89%). ^{1}H NMR (400 MHz, DMSO-*d*$_6$):δ= 8.31 (s, 1H), 8.04 (d, J = 7.8 Hz, 1H), 7.89 (d, J = 8.1 Hz, 1H), 7.79 (s, 1H), 7.48 (t, J = 7.6 Hz, 1H), 7.37 (t, J = 7.5 Hz, 1H), 6.21 (s, 1H), 5.76 (s, 1H), 4.88 (d, J = 4.8 Hz, 2H), 3.65 (d, J = 4.7 Hz, 2H), 3.45 (s, 2H), 3.08 (s, 3H), 1.41 (s, 6H). ^{13}H NMR (100 MHz, DMSO-*d*$_6$):δ= 170.50, 167.43, 165.18, 154.01, 140.23, 134.15, 127.13, 125.12, 122.45, 122.16, 119.13, 96.80, 58.36, 57.61, 55.39, 45.09, 41.17, 25.40. MS(ESI): m/z Calcd. For $C_{24}H_{21}N_3OS_3$ 463.1; found: 464.1, $[M+H]^+$.

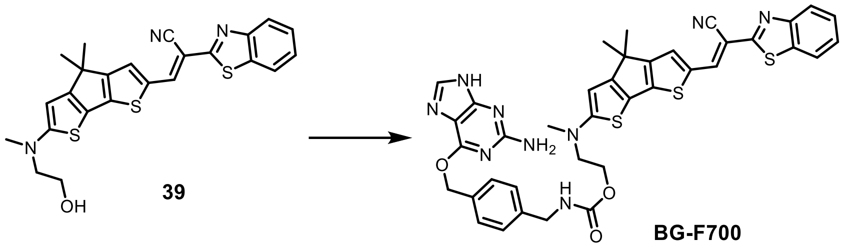

Compound BG-F700: This compound was obtained by following the general procedure for BG-F485. (0.182 g, yield 80%). ^{1}H NMR (400 MHz, DMSO-*d*$_6$):δ =12.51 (s, 1 H), 8.55 (t, J=5.8 Hz, 1 H)， 8.31 (s, 1H), 8.04 (d, J = 7.8 Hz, 1H), 7.79(s, 1 H), 7.89 (d, J = 8.1 Hz, 1H), 7.79 (s, 1H), 7.48 (t, J = 7.6 Hz, 1H), 7.44 (d, J=7.9 Hz, 2 H), 7.37 (t, J = 7.5 Hz, 1H), 7.30 (d, J=7.9 Hz, 2 H), 6.27(s, 2 H), 6.21 (s, 1H), 5.76 (s, 1H), 5.44 (s, 2 H), 4.88 (d, J = 4.8 Hz, 2H), 4,37 (d, J=5.8 Hz, 2 H), 3.65 (d, J = 4.7 Hz, 2H), 3.45 (s, 2H), 3.08 (s, 3H), 1.41 (s, 6H). ^{13}H NMR (100 MHz, DMSO-*d*$_6$): δ =170.50, 167.43, 165.18, 159.63, 155.46, 154.01, 140.23, 139.11, 137.68, 135.21, 134.15, 128.85, 127.95, 127.13, 125.12, 122.45, 122.16, 119.13, 96.80, 66.59, 58.36, 57.61, 55.39, 45.09, 42.87, 42.98, 41.17, 25.40. HR-MS(ESI): m/z Calcd. For $C_{38}H_{33}N_9O_3S_3$

759.1868; found 782.1870, [M+Na]$^+$.

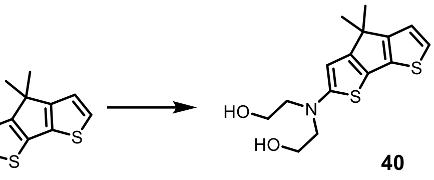

Compound 40: This compound was obtained by following the general procedure for compound 18. (0.319 g, yield 39%). ^{1}H NMR (400 MHz, DMSO-d_6): δ= 7.16 (s, 1 H), 6.76(q, J =5.2 Hz, 2 H), 4.79 (t, J =4.8 Hz, 1 H), 3.84 (t, J =4.8 Hz, 4 H), 3.48 (t, J =4.8 Hz, 4 H), 1.46(s, 6 H). ^{13}H NMR (100 MHz, DMSO-d_6): δ=158.61, 155.22, 136.81, 125.04, 124.53,111.09, 110.49, 60.40, 52.11, 50.80, 31.70. MS(ESI): m/z Calcd. For $C_{15}H_{19}NO_2S_2$ 309.1; found 310.1, [M+H]$^+$.

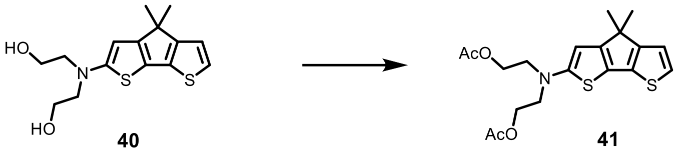

Compound 41: This compound was obtained by following the general procedure for compound 9. (0.325 g, yield 95%). ^{1}H NMR (400 MHz, DMSO-d_6): δ= 7.16 (s, 1 H), 6.77(q, J =5.2 Hz, 2 H), 4.79 (t, J =4.8 Hz, 1 H), 3.85 (t, J =4.8 Hz, 4 H), 3.40 (t, J =4.8 Hz, 4 H), 2.05 (s, 6H), 1.46(s, 6 H). ^{13}H NMR (100 MHz, DMSO-d_6): δ=171.03, 158.61, 155.22, 136.81, 125.04, 124.53, 111.09, 110.49, 60.40, 52.11, 50.80, 31.67, 20.96. MS(ESI): m/z Calcd. For $C_{19}H_{23}NO_4S_2$ 393.1; found 394.1, [M+H]$^+$.

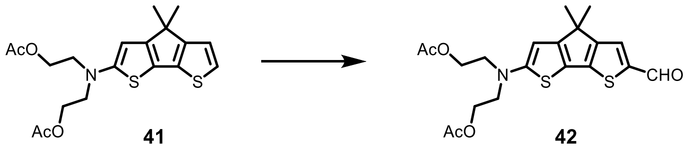

Compound 42: This compound was obtained by following the general procedure for compound 9. (0.296 g, yield 65%). ^{1}H NMR (400 MHz, DMSO-d_6): δ= 9.66, 7.46 (s, 1 H), 6.76(q, J =5.2 Hz, 2 H), 4.79 (t, J =5.2 Hz, 1 H), 3.85 (t, J =4.8 Hz, 4 H), 3.40 (t, J =4.8 Hz, 4 H), 2.05 (s, 6 H), 1.46(s, 6 H). ^{13}H NMR (100 MHz, DMSO-d_6): δ=158.61, 155.22, 136.81, 125.04, 124.53,111.09, 110.49, 60.40, 52.11, 50.80, 31.70, 20.90. MS(ESI): m/z Calcd. For $C_{20}H_{123}NO_5S_2$ 421.1; found 422.1, [M+H]$^+$.

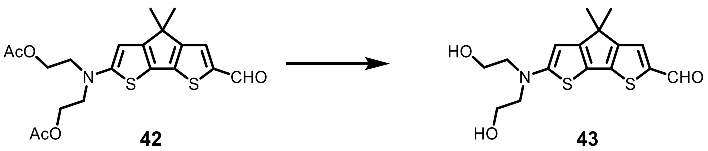

Compound 43: This compound was obtained by following the general procedure for compound 9. (0.296 g, yield 65%). ^{1}H NMR (400 MHz, DMSO-d_6): δ= 9.66 (s, 1 H), 7.16 (s, 1 H), 6.76(q, J =5.2 Hz, 2 H), 4.79 (t, J =5.2 Hz, 1 H), 3.84 (t, J =4.8 Hz, 4 H), 3.48 (t, J =4.8 Hz, 4 H),

1.46(s, 6 H). ^{13}H NMR (100 MHz, DMSO-d_6): δ=170.11, 160.61, 157.22, 136.81, 125.04, 124.53,111.09, 110.49, 60.40, 52.11, 50.80, 31.70. MS(ESI): m/z Calcd. For $C_{15}H_{19}NO_2S_2$ 309.1; found 310.1, [M+H]$^+$.

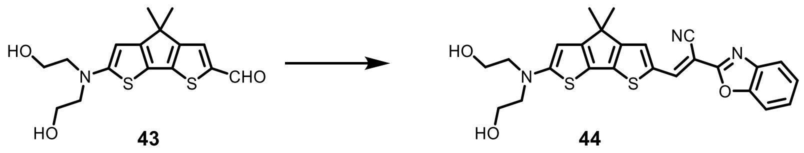

Compound 44: This compound was obtained by following the general procedure for compound 9. (0.271 g, yield 67%). ^{1}H NMR (400 MHz, DMSO-d_6): δ= 8.37 (s, 1H), 7.80 (s, 1H), 7.70 – 7.64 (m, 2H), 7.37 – 7.30 (m, 2H), 6.27 (s, 1H), 3.67 (t, *J* = 5.6 Hz, 4H), 3.52 (t, *J* = 5.6 Hz, 4H), 1.41 (s, 6H). ^{13}H NMR (100 MHz, DMSO-d_6): δ=171.15, 166.96, 161.26, 159.63, 152.30, 149.90, 141.94, 140.71, 131.92, 124.73, 124.38, 118.63, 115.24, 110.16, 96.33, 81.23, 69.71, 57.71, 56.14, 44.52, 24.87. MS(ESI): m/z Calcd. For $C_{25}H_{23}N_3O_3S_2$ 477.1; found 461.2, [M+H]$^+$.

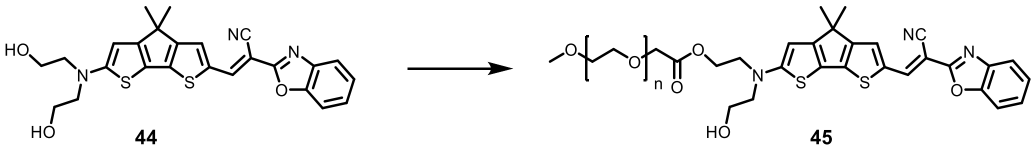

Compound 45: This compound was obtained by following the general procedure for compound 45. (0.62 g, yield 58%). ^{1}H NMR (400 MHz, DMSO-d_6): δ= 8.37 (s, 1H), 7.80 (s, 1H), 7.68 (m, 2H), 7.35 (m, 2H), 6.27 (s, 1H), 4.03 (s, 2H), 3.58-5.44 (m, 190H), 3.25 (s, 3H), 1.41 (s, 6H). ^{13}H NMR (100 MHz, DMSO-d_6): δ=171.90, 171.15, 166.96, 161.26, 159.63, 152.30, 149.90, 141.94, 140.71, 131.92, 124.73, 124.38, 118.63, 115.24, 110.16, 96.33, 81.23, 71.30, 69.91, 69.71, 67.54, 58.10, 57.71, 56.14, 44.52, 24.87. MALDI-TOF spectrum exhibited the center of the peak at m/z 2486.3, [M+H]$^+$.

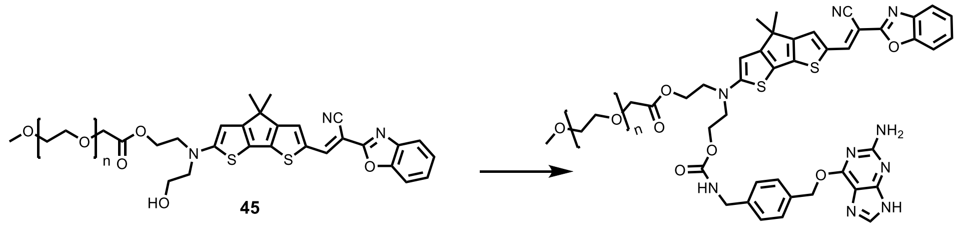

PEG-BG-F680

Compound PEG-BG-680: This compound was obtained by following the general procedure for BG-F485. (0.582 g, yield 51%). ^{1}H NMR (400 MHz, DMSO-d_6): δ= 12.40 (s, 1H), 8.37 (s, 1H), 7.91 (s, 1H), 7.80 (s, 1H), 7.68 (m, 2H), 7.43 (d, J=7.9, 2H), 7.35 (m, 2H), 7.23 (d, J=7.9, 2H), 6.27 (m, 2 H), 5.46 (s, 2H), 4.15 (d, *J* = 6.0 Hz, 2H), 4.05 (s, 2 H), 3.57-3.44 (m, 190H),

3.25 (s, 3H), 1.41 (s, 6H). ^{13}H NMR (100 MHz, DMSO-*d*$_6$): δ=171.90, 171.15, 166.96, 161.26, 159.63, 152.30, 149.90, 141.94, 140.71, 131.92, 124.73, 124.38, 118.63, 115.24, 110.16, 96.33, 81.23, 71.30, 69.91, 69.71, 67.54, 58.10, 57.71, 56.14, 44.52, 24.87. MALDI-TOF spectrum exhibited the center of the peak at m/z 2782.4, [M+H]$^+$.

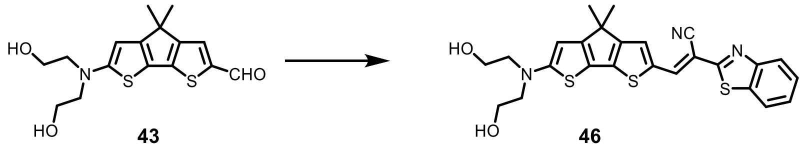

Compound 46: This compound was obtained by following the general procedure for compound 9. (0.296 g, yield 65%). ^{1}H NMR (400 MHz, DMSO-*d*$_6$): δ= 8.32 (s, 1H), 8.05 (d, *J* = 8.0 Hz, 1H), 7.90 (d, *J* = 8.0 Hz, 1H), 7.80 (s, 1H), 7.49 (t, *J* = 7.6 Hz, 1H), 7.37 (t, *J* = 7.6 Hz, 1H), 6.25 (s, 1H), 3.66 (t, *J* = 5.6 Hz, 4H), 3.52 (t, *J* = 5.6 Hz, 4H), 1.41 (s, 7H). ^{13}H NMR (100 MHz, DMSO-*d*$_6$): δ=169.99, 166.43, 164.68, 156.32, 153.49, 151.34, 139.70, 133.65, 131.92, 130.80, 126.60, 124.59, 121.92, 121.62, 118.64, 115.26, 106.37, 96.27, 89.44, 57.71, 56.12, 44.55, 40.34, 24.87. MS(ESI): m/z Calcd. For $C_{25}H_{23}N_3O_2S_3$ 493.1; found 494.1, [M+H]$^+$.

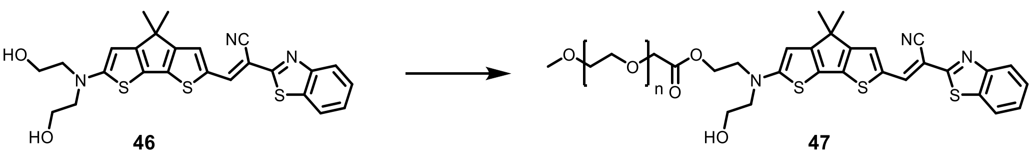

Compound 47: This compound was obtained by following the general procedure for compound 9. (0.296 g, yield 65%). ^{1}H NMR (400 MHz, DMSO-*d*$_6$): δ= 8.32 (s, 1H), 8.05 (d, *J* = 8.0 Hz, 1H), 7.90 (d, *J* = 8.0 Hz, 1H), 7.80 (s, 1H), 7.49 (t, *J* = 7.6 Hz, 1H), 7.37 (t, *J* = 7.6 Hz, 1H), 6.25 (s, 1H), 4.05 (s, 2 H), 3.57-3.44 (m, 190H), 3.25 (s, 3H), 1.41 (s, 7H). ^{13}H NMR (100 MHz, DMSO-*d*$_6$): δ=171.91, 169.99, 166.43, 164.68, 156.32, 153.49, 151.34, 139.70, 133.65, 131.92, 130.80, 126.60, 124.59, 121.92, 121.62, 118.64, 115.26, 106.37, 96.27, 89.44, 71.20, 69.70, 68.94, 67.48, 57.96, 57.71, 56.12, 44.55, 40.34, 24.87. MALDI-TOF spectrum exhibited the center of the peak at m/z 2502.3, [M+H]$^+$.

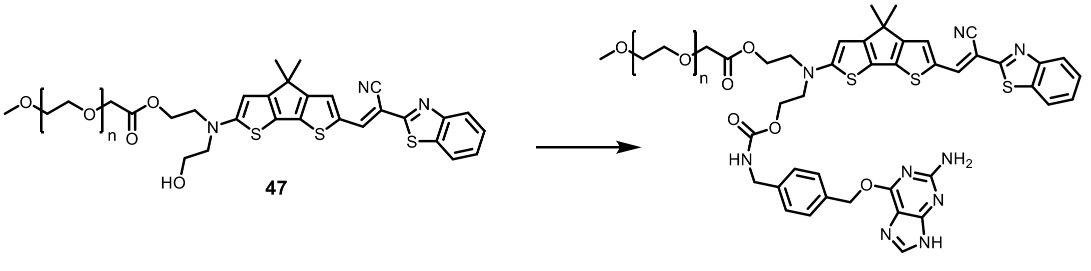

PEG-BG-F700

Compound PEG-BG-700: This compound was obtained by following the general

procedure for BG-F485. (0.610 g, yield 48%). 12.41 (s, 1 H), 8.32 (s, 1H), 8.05 (d, *J* = 8.0 Hz, 1H), 7.90 (d, *J* = 8.0 Hz, 1H), 7.80 (s, 1H), 7.49 (t, *J* = 7.6 Hz, 1H), 7.43 (s, 1 H), 7.37 (t, *J* = 7.6 Hz, 1H), 7.24 (d, J=7.2 Hz, 2 H), 6.32 (s, 2 H), 6.25 (s, 1H), 5.45 (s, 2 H), 4.15 (d, J=6.0 Hz, 2 H), 4.05 (s, 2 H), 3.57-3.44 (m, 190H), 3.25 (s, 3H), 1.41 (s, 7H). ^{13}H NMR (100 MHz, DMSO-d_6): δ=171.91, 169.99, 166.43, 164.68, 159.92, 158, 62, 156.32, 155.81, 153.49, 151.34, 142.21, 139.70, 137.81, 135.21, 133.65, 131.92, 130.80, 128.50, 127.10, 126.60, 124.59, 121.92, 121.62, 118.64, 115.26, 113.50, 106.37, 96.27, 89.44, 77.80, 71.20, 69.70, 68.94, 67.48, 57.96, 57.71, 56.12, 44.55, 40.34, 28.61, 24.87. MALDI-TOF spectrum exhibited the center of the peak at m/z 2798.4, [M+H]$^+$.

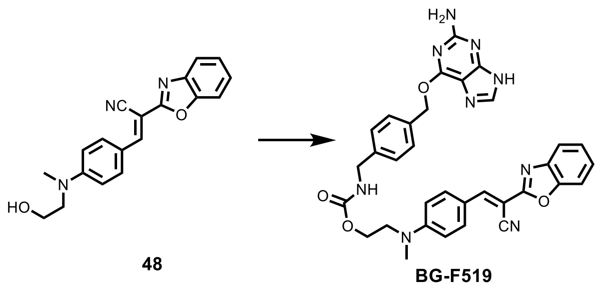

48 BG-F519

Compound BG-F519: This compound was obtained by following the general procedure for BG-F485. (0.290 g, yield 88%).^{1}H-NMR（400 MHz，DMSO-d_6）：δ=12.41 (s, 1H), 10.01 (s, 1H), 8.10 (m, 2H), 7.95 (m, 3H), 7.81 (s, 1 H), 7.70 (d, 1H, J=8.8 Hz), 7.45 (m, 1H), 7.41 (m, 4H), 6.29 (s, 2H), 6.27 (dd, J=9.2, 1.6 Hz, 1H), 6.02 (s, 1H), 5.46 (s, 2H), 4.40 (d, J=4.9 Hz, 2H), 3.88 (t, J=5.6 Hz, 2H), 3.64 (t, J=5.6 Hz, 2H), 3.15 (s, 3H). MS(ESI): m/z Calcd. For $C_{33}H_{29}N_9O_4$ 615.2343; found 638.2241, [M+Na]$^+$

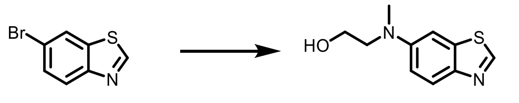

49

Compound 49: This compound was obtained by following the general procedure for compound 18. (0.510 g, yield 69%).^{1}H NMR (DMSO-d_6, 400 MHz): δ= 8.67(s，1 H), 7.95(d, J=8.8 Hz, 1 H), 7.15(d, J=2.4 Hz, 1 H), 7.00 (dd, J=8.8, 2.4 Hz，1 H), 4.69 (bt, 1H), 3.57 (t, J =6.0Hz, 2H), 3.44(t, J =6.4 Hz, 2H), 2.97 (s, 3H). 13C NMR (DMSO-d6, 100 MHz): δ=147.05, 141.41, 129.71, 123.58, 121.37, 111.65, 103.40, 58.10, 54.72, 38.97. MS(ESI):m/z calcd for C11H13NOS 208.1; found 209.1, [M+H]$^+$.

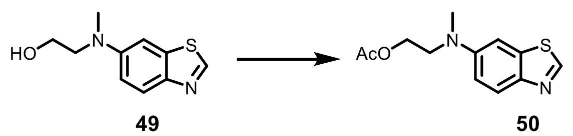

49 50

Compound 50: This compound was obtained by following the general procedure for

compound 9. (0.480 g, yield 97%).^{1}H NMR (DMSO-d6, 400 MHz): δ= 8.67(s, 1 H), 7.95(d, J=8.8 Hz, 1 H), 7.15(d, J=2.4 Hz, 1 H), 7.00(dd, J=8.8, 2.4 Hz, 1 H), 4.69 (bt, 1H), 3.97 (t, J =6.0Hz, 2H), 3.44(t, J =6.4 Hz, 2H), 2.97 (s, 3H), 2.05 (s, 3H). ^{13}C NMR (DMSO-d_6, 100 MHz): δ=171.08, 147.05, 141.41, 129.71, 123.58, 121.37, 111.65, 103.40, 58.10, 51.72, 38.97, 20.96. MS(ESI):m/z calcd for $C_{12}H_{14}N_2O_2S$ 208.1; found 251.1, $[M+H]^+$.

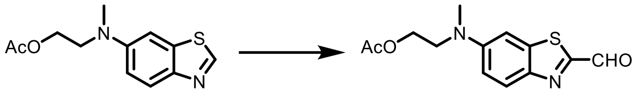

50 **51**

Compound 51: This compound was obtained by following the general procedure for compound 10. (0.362 g, yield 71%). ^{1}H NMR (DMSO-d_6, 400 MHz): δ=10.05(s, 1 H),8.02(d, J=9.8 Hz, 1 H), 7.05(dd, J=9.9 2.4 Hz, 1 H), 7.00(d, J= 2.4 Hz, 1 H), 4.69 (bt, 1H), 3.57 (t, J =6.0Hz, 2H), 3.44(t, J =6.4 Hz, 2H), 3.01 (s, 3H), 2.05 (s, 3 H). ^{13}C NMR (DMSO-d_6, 100 MHz): δ=185.21, 171.10, 160.21, 150.86, 145.65, 140.12, 126.26, 114.90, 58.10, 54.72,38.97, 28.12. MS(ESI):m/z calcd for $C_{13}H_{14}N_2O_3S$ 278.1 ; found 279.1, $[M+H]^+$.

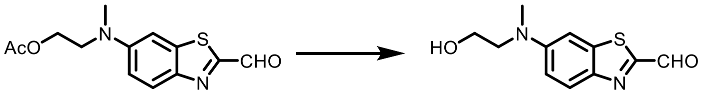

51 **52**

Compound 52: This compound was obtained by following the general procedure for compound 11. (0.273 g, yield 91%).^{1}H NMR (DMSO-d_6, 400 MHz): δ=10.05(s, 1 H),8.02(d, J=9.8 Hz, 1 H), 7.05(dd, J=9.9 2.4 Hz, 1 H), 7.00(d, J= 2.4 Hz, 1 H), 4.69 (bt, 1H), 3.57 (t, J =6.0Hz, 2H), 3.44(t, J =6.4 Hz, 2H), 3.01 (s, 3H). ^{13}C NMR (DMSO-d6, 100 MHz): δ=185.21, 160.21, 150.86, 145.65, 140.12, 126.26, 114.90, 58.10, 54.72, 38.97. MS (ESI):m/z calcd 236.1 ; found 237.1, $[M+H]^+$.

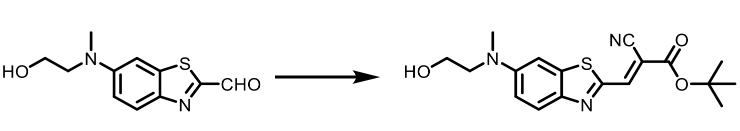

52 **53**

Compound 53: This compound was obtained by following the general procedure for compound 7. (0.215 g, yield 79%). ^{1}H NMR (DMSO-d6, 400 MHz): δ=8.00(s, 1 H), 7.65(d, J=9.2 Hz, 1 H), 7.08(d, J= 9.2 Hz, 1 H),7.02(d, J=2.4 Hz, 1 H), 4.69 (bt, 1H), 3.57 (t, J =6.0Hz, 2H), 3.44(t, J =6.4 Hz, 2H), 3.01 (s, 3H). ^{13}C NMR (DMSO-d6, 100 MHz): δ=169.84,151.32,151.22, 150.86, 145.54, 141.12, 126.26, 114.90,113.99, 111.99, 100.67, 81.65, 58.10, 54.72,38.97. MS(ESI):m/z calcd 359.1 ; found 360.1, $[M+H]^+$.

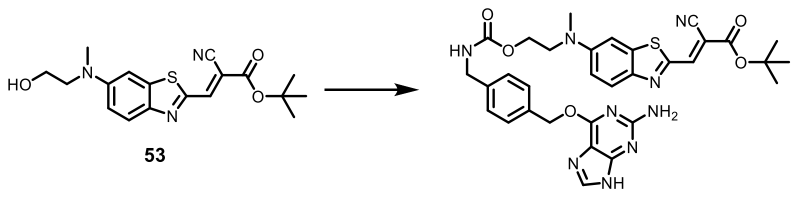

53　　BG-F624

Compound BG-F624: This compound was obtained by following the general procedure for BG-F485. (0.115 g, yield 76%). ^{1}H NMR (DMSO-d$_6$, 400 MHz): δ =12.51 (br, 1 H), 8.40 (s, 1 H), 8.02 (s, 1 H), 7.71 (m, 1 H), 7.32 (d, 2 H, J=8.0 Hz), 7.30 (s, 1 H), 7.22 (d, 2 H, J=8.0 Hz), 7.02 (dd, J=2.0, 9.2 Hz, 1 H), 6.30 (br, 2 H), 5.44 (s, 2 H), 4.17 (m, 4 H), 3.71 (t, 2 H, J=5.6 Hz), 3.06 (s, 3 H), 1.51 (s, 9 H). ^{13}C NMR (DMSO-d6, 100 MHz): δ=161.73, 159.56, 156.29, 149.81, 147.38, 146.43, 139.52, 139.12, 135.17, 129.83, 128.45, 128.01, 127.00, 126.76, 116.53, 113.12, 102.47, 96.32, 82.42, 66.53, 61.12, 50.80, 45.51, 43.52, 30.93, 27.62, 22.04, 13.94, 8.50. HRMS(ESI):m/z calcd for $C_{33}H_{33}N_9O_5S$ 655.2325; found 675.2323, $[M+Na]^+$.

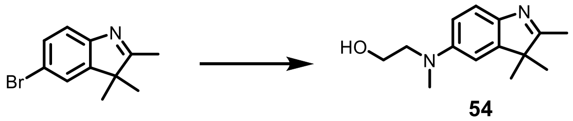

54

Compound 54: This compound was obtained by following the general procedure for compound 18. (0.530 g, yield 81%).^{1}H NMR (400 MHz, CDCl$_3$) δ 7.40 (d, *J* = 8.4 Hz, 1H), 6.78 (d, *J* = 2.4 Hz, 1H), 6.74 (dd, *J* = 8.5, 2.5 Hz, 1H), 3.83 (t, *J* = 5.6 Hz, 2H), 3.49 (t, *J* = 5.6 Hz, 2H), 2.99 (s, 3H), 2.27 (s, 3H), 1.29 (s, 6H). ^{13}C NMR (101 MHz, CDCl$_3$) δ 185.07, 149.33, 146.91, 119.88, 112.43, 107.39, 59.92, 56.50, 53.60, 39.42, 23.39. MS(ESI):m/z calcd for $C_{14}H_{20}N_2O$ 232.2 ; found 233.1, $[M+H]^+$.

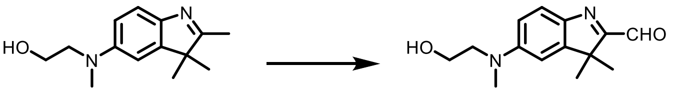

54　　55

Compound 55: To a clear solution of Compound 56 (0.46 g, 2.0 mmol) in 100 mL dioxane, SeO_2 (0.275 g, 2.5 mmol) was added under Ar atmosphere and the mixture was stired at rt for 5 hrs. Then the solvent was removed under reduce pressure to give the crude product which was purified by silica gel column chromatography to afford the purple compound 55. ^{1}H NMR (400 MHz, CDCl$_3$) δ 9.90 (s, 1H), 7.67 (d, *J* = 8.7 Hz, 1H), 6.78 (dd, *J* = 8.8, 2.6 Hz, 1H), 6.69 (d, *J* = 2.5 Hz, 1H), 3.89 (t, *J* = 5.7 Hz, 2H), 3.70 (s, 3H), 3.70 (s, 3H), 3.61 (t, *J* = 5.7 Hz, 2H), 3.11 (s, 3H), 1.45 (s, 6H). ^{13}C NMR (101 MHz, CDCl$_3$) δ 188.54, 176.25, 151.30, 143.51, 124.92, 111.99, 104.29, 72.79, 67.09, 60.14, 55.17, 52.06, 39.45, 22.95. MS(ESI):m/z calcd for $C_{14}H_{18}N_2O_2$ 246.1 ; found 247.1, $[M+H]^+$.

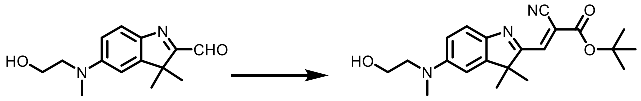

55　　56

Compound 56: This compound was obtained by following the general procedure for compound 7. (0.280 g, yield 89%). ^{1}H NMR (400 MHz, $CDCl_3$) δ 8.21 (s, 1H), 7.67 (d, *J* = 8.7 Hz, 1H), 6.78 (dd, *J* = 8.8, 2.6 Hz, 1H), 6.69 (d, *J* = 2.5 Hz, 1H), 3.89 (t, *J* = 5.7 Hz, 2H), 3.70 (s, 3H), 3.70 (s, 3H), 3.61 (t, *J* = 5.7 Hz, 2H), 3.11 (s, 3H), 1.50 (s, 9 H), 1.45 (s, 6H). ^{13}C NMR (101 MHz, $CDCl_3$) δ 171.54, 176.25, 151.30, 143.51, 124.92, 111.99, 104.29, 72.79, 67.09, 60.14, 55.17, 52.06, 39.45, 27.63, 22.95. MS(ESI):m/z calcd for $C_{21}H_{27}N_3O_3$ 369.2 ; found 370.2, $[M+H]^+$.

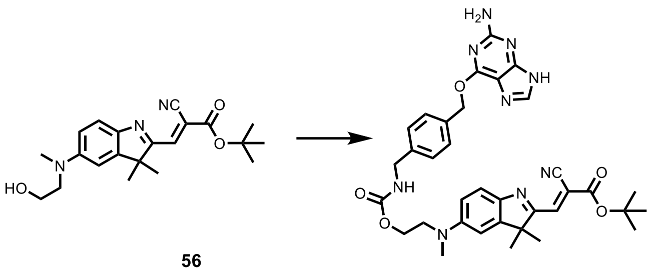

56

BG-F675

Compound BG-F675: This compound was obtained by following the general procedure for BG-F485. (0.295 g, yield 81%). ^{1}H NMR (400 MHz, $CDCl_3$) δ 12.41 (s, 1 H), 8.20 (s, 1H), 7.80 (s, 1 H), 7.67 (d, *J* = 8.7 Hz, 1H), 7.43 (d, *J* = 8.0 Hz, 2H), 7.24 (d, *J* = 8.0 Hz, 2H), 6.78 (dd, *J* = 8.8, 2.6 Hz, 1H), 6.69 (d, *J* = 2.5 Hz, 1H), 6.29 (s, 2 H), 5.45 (s, 2 H),4.13 (d, J=6.0 Hz, 2 H), 3.89 (t, *J* = 5.7 Hz, 2H), 3.70 (s, 3H), 3.70 (s, 3H), 3.61 (t, *J* = 5.7 Hz, 2H), 3.11 (s, 3H), 1.50 (s, 9 H), 1.45 (s, 6H). ^{13}C NMR (101 MHz, $CDCl_3$) δ 171.54, 176.25, 159.82, 158.61, 155.80, 155.21, 151.30, 143.51, 140.10, 137.81, 135.11, 128.51, 126.89, 124.92, 113,49, 111.99, 104.29, 77.80, 72.79, 67.09, 60.14, 55.17, 52.06, 43,21, 39.45, 28.36, 27.63, 22.95. HRMS(ESI):m/z calcd for $C_{35}H_{39}N_9O_5$ 665.3074; found 688.2972, $[M+Na]^+$.

Supplementary References

1. Filonov, G.S. & Verkhusha, V.V. A near-infrared BiFC reporter for in vivo imaging of protein-protein interactions. *Chemistry & biology* **20**, 1078-1086 (2013).
2. Greenwald, R.B., Choe, Y.H., McGuire, J. & Conover, C.D. Effective drug delivery by PEGylated drug conjugates. *Advanced drug delivery reviews* **55**, 217-250 (2003).
3. Nakai, J., Ohkura, M. & Imoto, K. A high signal-to-noise Ca(2+) probe composed of a single green fluorescent protein. *Nature biotechnology* **19**, 137-141 (2001).
4. Tallini, Y.N. et al. Imaging cellular signals in the heart in vivo: Cardiac expression of the high-signal Ca2+ indicator GCaMP2. *Proceedings of the National Academy of Sciences of the United States of America* **103**, 4753-4758 (2006).
5. Dana, H. et al. High-performance calcium sensors for imaging activity in neuronal populations and microcompartments. *Nature methods* **16**, 649-657 (2019).
6. Zhao, Y. et al. An expanded palette of genetically encoded Ca(2)(+) indicators. *Science* **333**, 1888-1891 (2011).
7. Hashizume, R. et al. A genetically encoded far-red fluorescent calcium ion biosensor derived from a biliverdin-binding protein. *Protein science : a publication of the Protein Society* **31**, e4440 (2022).
8. Shaner, N.C., Steinbach, P.A. & Tsien, R.Y. A guide to choosing fluorescent proteins. *Nature methods* **2**, 905-909 (2005).
9. Wang, L., Jackson, W.C., Steinbach, P.A. & Tsien, R.Y. Evolution of new nonantibody proteins via iterative somatic hypermutation. *Proceedings of the National Academy of Sciences of the United States of America* **101**, 16745-16749 (2004).
10. Shu, X. et al. Mammalian expression of infrared fluorescent proteins engineered from a bacterial phytochrome. *Science* **324**, 804-807 (2009).
11. Yu, D. et al. An improved monomeric infrared fluorescent protein for neuronal and tumour brain imaging. *Nature communications* **5**, 3626 (2014).
12. Lin, M.Z. et al. Autofluorescent proteins with excitation in the optical window for intravital imaging in mammals. *Chemistry & biology* **16**, 1169-1179 (2009).
13. Shcherbo, D. et al. Near-infrared fluorescent proteins. *Nature methods* **7**, 827-829 (2010).
14. Morozova, K.S. et al. Far-red fluorescent protein excitable with red lasers for flow cytometry and superresolution STED nanoscopy. *Biophysical journal* **99**, L13-15 (2010).
15. Piatkevich, K.D. et al. Extended Stokes Shift in Fluorescent Proteins: Chromophore-Protein Interactions in a Near-Infrared TagRFP675 Variant. *Scientific reports* **3** (2013).
16. Shcherbakova, D.M. & Verkhusha, V.V. Near-infrared fluorescent proteins for multicolor in vivo imaging. *Nature methods* **10**, 751-754 (2013).
17. Chu, J. et al. Non-invasive intravital imaging of cellular differentiation with a bright red-excitable fluorescent protein. *Nature methods* **11**, 572-578 (2014).
18. Yu, D. et al. A naturally monomeric infrared fluorescent protein for protein labeling in vivo. *Nature methods* **12**, 763-765 (2015).
19. Rodriguez, E.A. et al. A far-red fluorescent protein evolved from a cyanobacterial phycobiliprotein. *Nature methods* **13**, 763-769 (2016).
20. Shcherbakova, D.M. et al. Bright monomeric near-infrared fluorescent proteins as

tags and biosensors for multiscale imaging. *Nature communications* **7**, 12405 (2016).
21. Bajar, B.T. et al. Fluorescent indicators for simultaneous reporting of all four cell cycle phases. *Nature methods* **13**, 993-996 (2016).
22. Matela, G. et al. A far-red emitting fluorescent marker protein, mGarnet2, for microscopy and STED nanoscopy. *Chemical communications* **53**, 979-982 (2017).
23. Stohr, K. et al. Quenched substrates for live-cell labeling of SNAP-tagged fusion proteins with improved fluorescent background. *Analytical chemistry* **82**, 8186-8193 (2010).
24. Komatsu, T. et al. Real-time measurements of protein dynamics using fluorescence activation-coupled protein labeling method. *Journal of the American Chemical Society* **133**, 6745-6751 (2011).
25. Sun, X. et al. Development of SNAP-tag fluorogenic probes for wash-free fluorescence imaging. *Chembiochem : a European journal of chemical biology* **12**, 2217-2226 (2011).
26. Liu, X., Song, J., Kang, Y., Wang, Y. & Chen, A. Long noncoding RNA SOX21-AS1 regulates the progression of triple-negative breast cancer through regulation of miR-520a-5p/ORMDL3 axis. *Journal of cellular biochemistry* (2020).
27. Liu, T.K. et al. A rapid SNAP-tag fluorogenic probe based on an environment-sensitive fluorophore for no-wash live cell imaging. *ACS chemical biology* **9**, 2359-2365 (2014).
28. Yu, W.T., Wu, T.W., Huang, C.L., Chen, I.C. & Tan, K.T. Protein sensing in living cells by molecular rotor-based fluorescence-switchable chemical probes. *Chem Sci* **7**, 301-307 (2016).
29. Jung, K.H. et al. A SNAP-tag fluorogenic probe mimicking the chromophore of the red fluorescent protein Kaede. *Organic & biomolecular chemistry* **17**, 1906-1915 (2019).
30. Liu, Y. et al. Modulation of Fluorescent Protein Chromophores To Detect Protein Aggregation with Turn-On Fluorescence. *Journal of the American Chemical Society* **140**, 7381-7384 (2018).
31. Kang, M.G. et al. Structure-guided synthesis of a protein-based fluorescent sensor for alkyl halides. *Chemical communications* **53**, 9226-9229 (2017).
32. Liu, Y. et al. The Cation-pi Interaction Enables a Halo-Tag Fluorogenic Probe for Fast No-Wash Live Cell Imaging and Gel-Free Protein Quantification. *Biochemistry* **56**, 1585-1595 (2017).
33. Bachollet, S. et al. An expanded palette of fluorogenic HaloTag probes with enhanced contrast for targeted cellular imaging. *Organic & biomolecular chemistry* **20**, 3619-3628 (2022).
34. Lukinavicius, G. et al. A near-infrared fluorophore for live-cell super-resolution microscopy of cellular proteins. *Nature chemistry* **5**, 132-139 (2013).
35. Butkevich, A.N. et al. Fluorescent Rhodamines and Fluorogenic Carbopyronines for Super-Resolution STED Microscopy in Living Cells. *Angewandte Chemie* **55**, 3290-3294 (2016).
36. Grimm, J.B. et al. A general method to fine-tune fluorophores for live-cell and in vivo imaging. *Nature methods* (2017).
37. Sato, R. et al. Intracellular Protein-Labeling Probes for Multicolor Single-Molecule Imaging of Immune Receptor-Adaptor Molecular Dynamics. *Journal of the American*

Chemical Society **139**, 17397-17404 (2017).
38. Liu, Y. et al. AgHalo: A Facile Fluorogenic Sensor to Detect Drug-Induced Proteome Stress. *Angewandte Chemie* **56**, 8672-8676 (2017).
39. Fares, M. et al. A Molecular Rotor-Based Halo-Tag Ligand Enables a Fluorogenic Proteome Stress Sensor to Detect Protein Misfolding in Mildly Stressed Proteome. *Bioconjugate chemistry* **29**, 215-224 (2018).
40. Grimm, J.B. et al. A general method to optimize and functionalize red-shifted rhodamine dyes. *Nature methods* **17**, 815-821 (2020).
41. Grimm, J.B. et al. A General Method to Improve Fluorophores Using Deuterated Auxochromes. *JACS Au* **1**, 690-696 (2021).
42. Zhang, D. et al. Development of Acrylamide-Based Rapid and Multicolor Fluorogenic Probes for High Signal-to-Noise Live Cell Imaging. *Bioconjugate chemistry* **30**, 184-191 (2019).
43. Samanta, S.R., Da Silva, J.P., Baldridge, A., Tolbert, L.M. & Ramamurthy, V. A Latent Reaction in a Model GFP Chromophore Revealed upon Confinement: Photohydroxylation of ortho-Halo Benzylidene-3-methylimidazolidiones via an Electrocylization Process. *Organic Letters* **16**, 3304-3307 (2014).
44. Keppler, A. et al. A general method for the covalent labeling of fusion proteins with small molecules in vivo. *Nat Biotech* **21**, 86-89 (2003).
45. Los, G.V. et al. HaloTag: a novel protein labeling technology for cell imaging and protein analysis. *ACS chemical biology* **3**, 373-382 (2008).
46. Chen, Z., Jing, C., Gallagher, S.S., Sheetz, M.P. & Cornish, V.W. Second-Generation Covalent TMP-Tag for Live Cell Imaging. *J. Am. Chem. Soc.* **134**, 13692-13699 (2012).
